# Supplementary material for: circARHGAP10 as a candidate biomarker and therapeutic target in myotonic dystrophy type 1
Source: Mol Ther Nucleic Acids. 2025 Jul 30;36(3):102646. doi: 10.1016/j.omtn.2025.102646 (PMC12395532; doi:10.1016/j.omtn.2025.102646)
Supplement: Document S1. Figures S1–S14 [file mmc1.pdf]

OMTN, Volume *36*

Supplemental information

circARHGAP10 as a candidate biomarker and therapeutic target in myotonic dystrophy type 1

Denisa Baci, Spyros Tastsoglou, Claudia Provenzano, Alessandra Perfetti, Mariapaola Izzo, Mario Lisanti, Svetlana Frolova, Christine Voellenkle, Anna Sofia Tascini, Rosanna Cardani, Beatrice Cardinali, Giovanni Meola, Germana Falcone, and Fabio Martelli

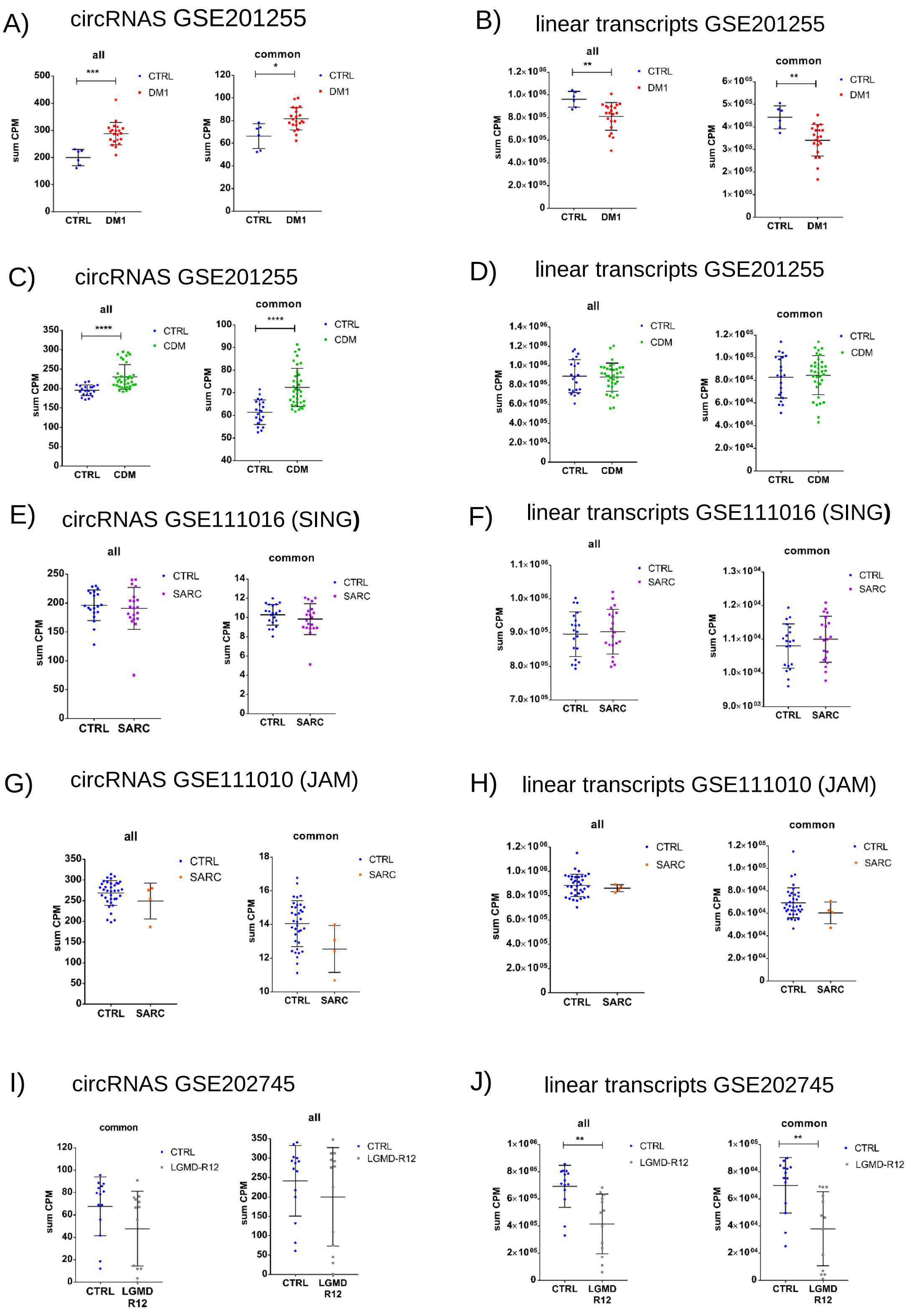
A) circRNAS GSE201255
all
common
CTRL
DM1
sum CPM
B) linear transcripts GSE201255
all
common
CTRL
DM1
sum CPM
C) circRNAS GSE201255
all
common
CTRL
CDM
sum CPM
D) linear transcripts GSE201255
all
common
CTRL
CDM
sum CPM
E) circRNAS GSE111016 (SING)
all
common
CTRL
SARC
sum CPM
F) linear transcripts GSE111016 (SING)
all
common
CTRL
SARC
sum CPM
G) circRNAS GSE111010 (JAM)
all
common
CTRL
SARC
sum CPM
H) linear transcripts GSE111010 (JAM)
all
common
CTRL
SARC
sum CPM
I) circRNAS GSE202745
common
all
CTRL
LGMD-R12
LGMD R12
sum CPM
J) linear transcripts GSE202745
all
common
CTRL
LGMD-R12
LGMD R12
sum CPM

Figure S1. Assessment of global circRNA levels in muscle tissues across various myopathies.

Barplots showing global circRNA levels (A, C, E, G, I) and their corresponding linear transcripts (B, D, F, H, J). (A-B) Contrast between adult DM1 (n=22) and control (CTRL, n=7) samples from the GSE201255 dataset. (C-D) Contrast between congenital DM1 (CDM, n=36) and pediatric controls (n=21) from the GSE201255 dataset. (E-F) Contrast between sarcopenia (SARC, n=20) and control samples (n=20) from the Singapore cohort ("SING") in the GSE111016 dataset. (G-H) Contrast between sarcopenia (n=9) and control samples (n=30) from the Jamaica cohort ("JAM") in the GSE111010 dataset. (I-J) Contrast between Limb Girdle Muscular Dystrophy R12 (LGMD-R12, n=13) and control samples (n=14) in the GSE202745 dataset. Each point represents the sum of counts-per-million (CPM) reads mapped to the transcriptome, normalized for library depth. Two contrasts are shown for each panel: on the left ("all"), values of all expressed circRNAs (or their linear transcripts) are summed for each sample and compared between disease and control; on the right ("common"), only circRNAs expressed in at least all-but-one sample in each cohort are included. For LGMD-R12 (I-J), a less stringent criterion was used due to library size limitations. DM1 vs. CTRL comparisons were performed using a two-sided Welch's t-test (*$p < 0.05$, **$p < 0.01$, ***$p < 0.001$, ****$p < 0.0001$).

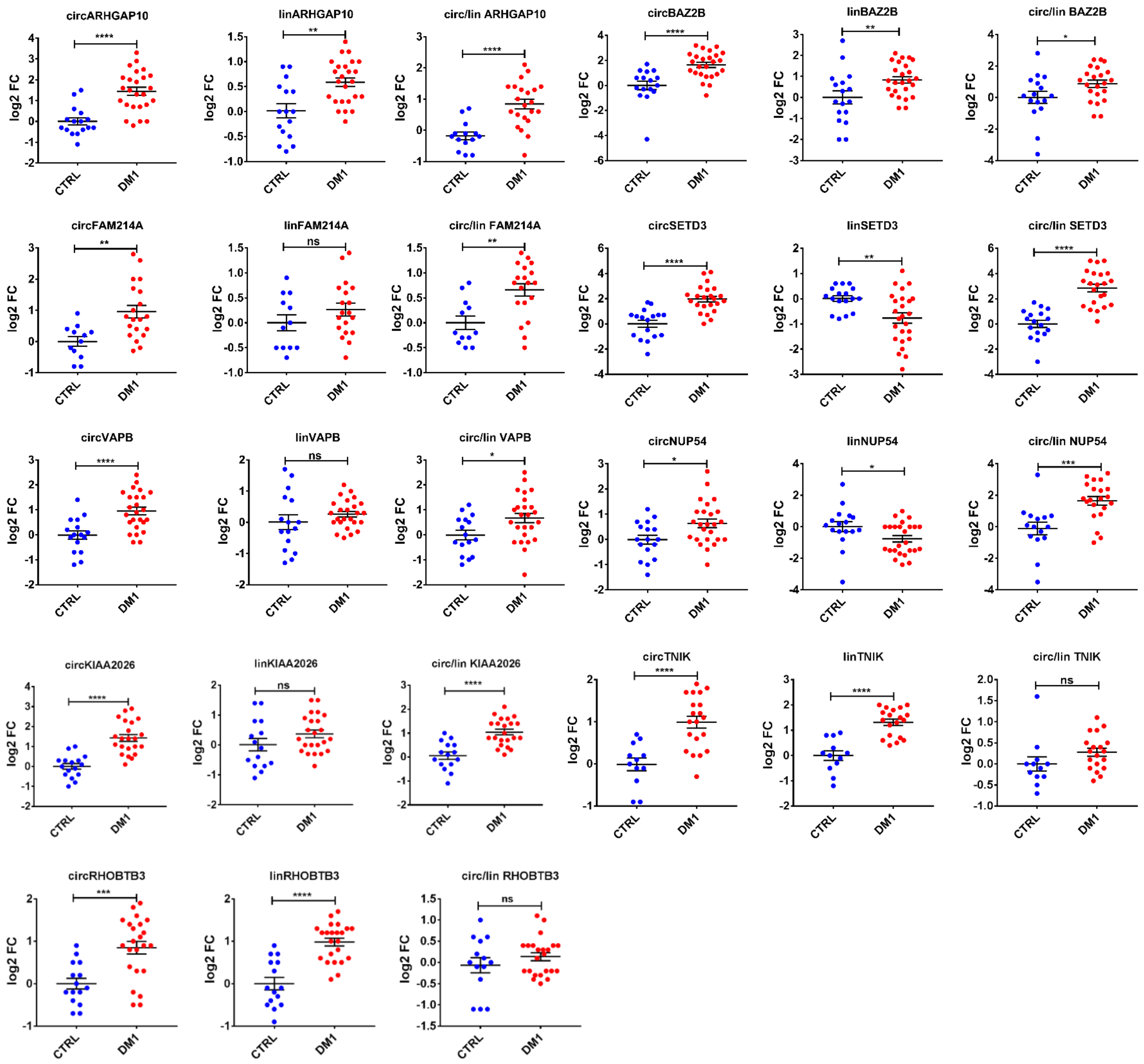

Figure S2. qPCR validation of DM1-circRNA candidates.

qPCR validation of DM1-circRNA expression levels and circ/lin ratios in biceps brachii samples from DM1 and control (CTRL) groups. Barplots show increased expression of nine DM1-circRNAs in DM1 samples compared to controls, along with significantly altered circ/lin ratios, except for RHOBTB3 and TNIK. Data are shown on a $\log_2$ scale. Differences between groups were evaluated using the Mann–Whitney U test. CTRL (blue dots, n = 16); DM1 (red dots, n = 24) (*$p < 0.05$, **$p < 0.01$, ***$p < 0.001$, ****$p < 0.0001$).

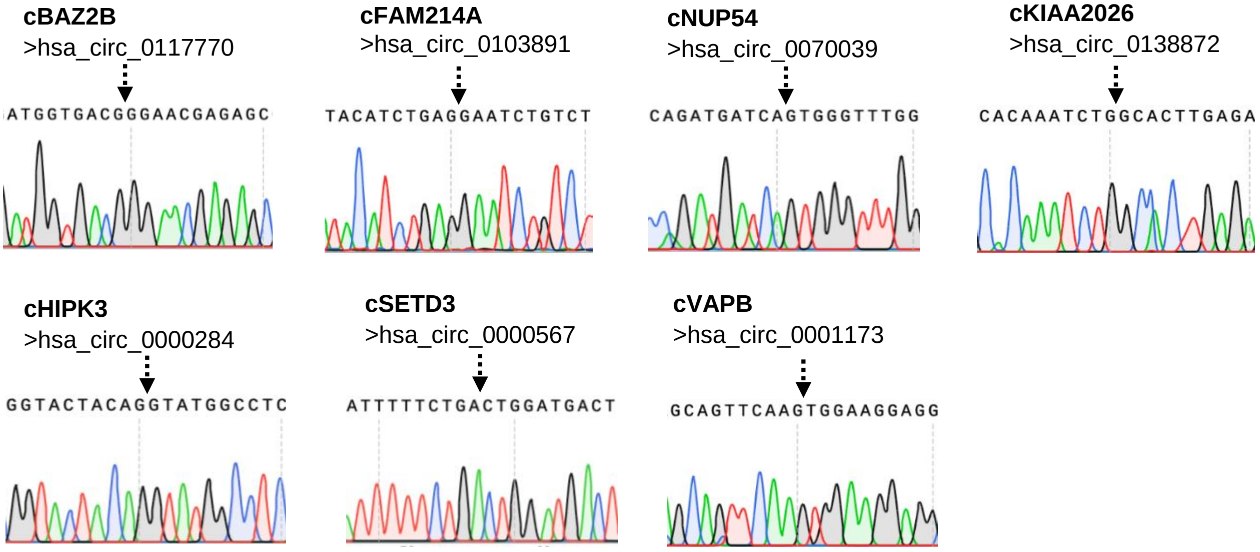

Figure S3. Sanger sequencing of DM1-circRNA candidates expressed in biceps brachii samples from DM1 patients.
For each circRNA, the expected back-splice junction sequence was confirmed. The arrow and chromatograms show the position of the back-splice junction, validating the circular structure of the transcripts.

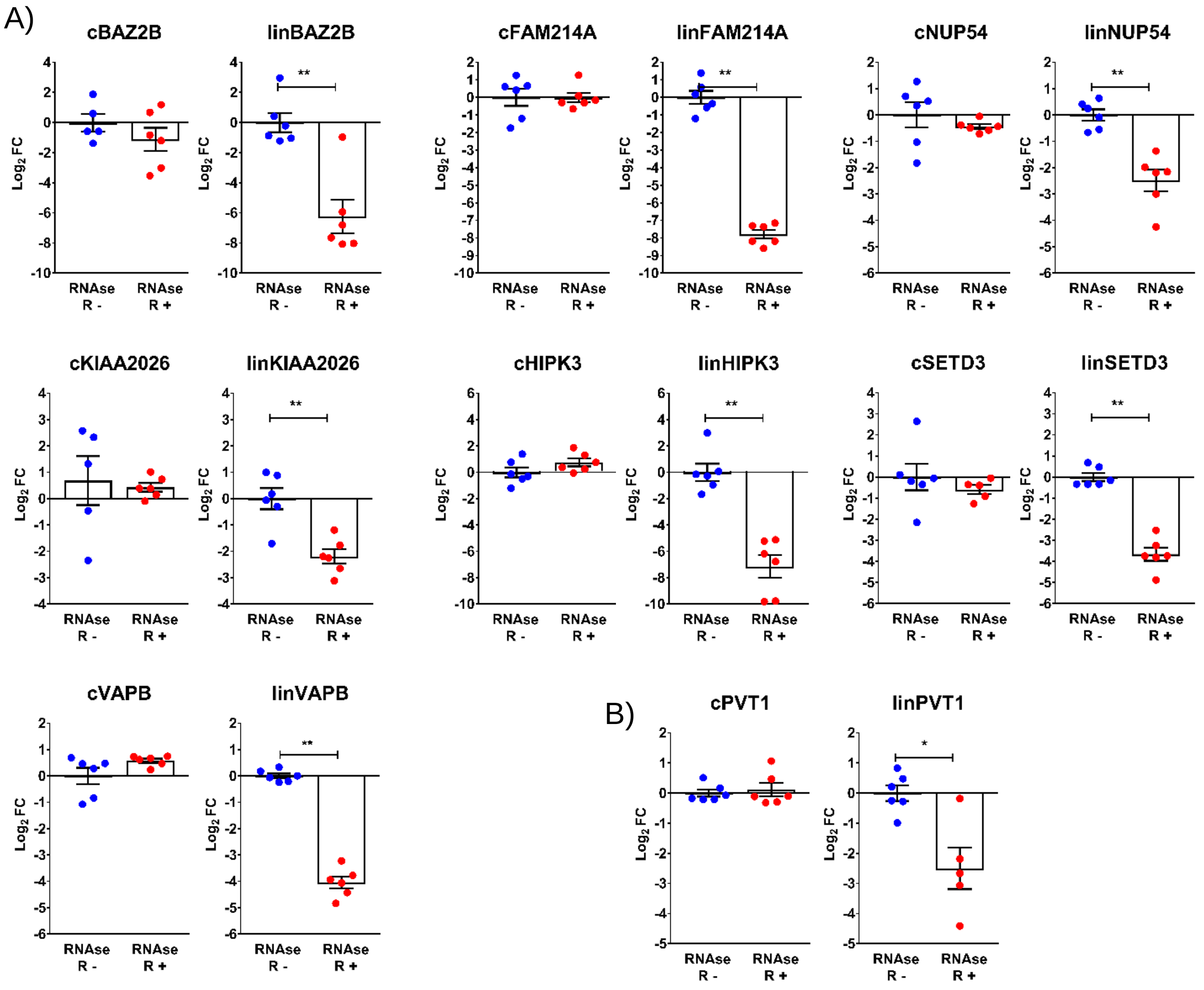

Figure S4. DM1-circRNA candidates are resistant to RNase R digestion. (A) qPCR analysis of selected DM1-associated circRNA candidates and their corresponding linear transcripts was performed on total RNA treated with (+RNase R) or without (−RNase R) RNase R exonuclease. CircRNAs exhibited resistance to RNase R treatment, while the corresponding linear transcripts were significantly degraded. (B) RNase R resistance of circPVT1 (cPVT1) and linear PVT1 (linPVT1) was assessed as a control. As expected, cPVT1 was resistant to RNase R digestion, whereas linPVT1 was significantly degraded, confirming effective exonuclease treatment. Data are presented as mean ± SEM on a $\log_2$ scale ($n = 5–6$). Statistical significance was determined using a two-tailed Mann–Whitney U test ($*p < 0.05$, $**p < 0.01$).

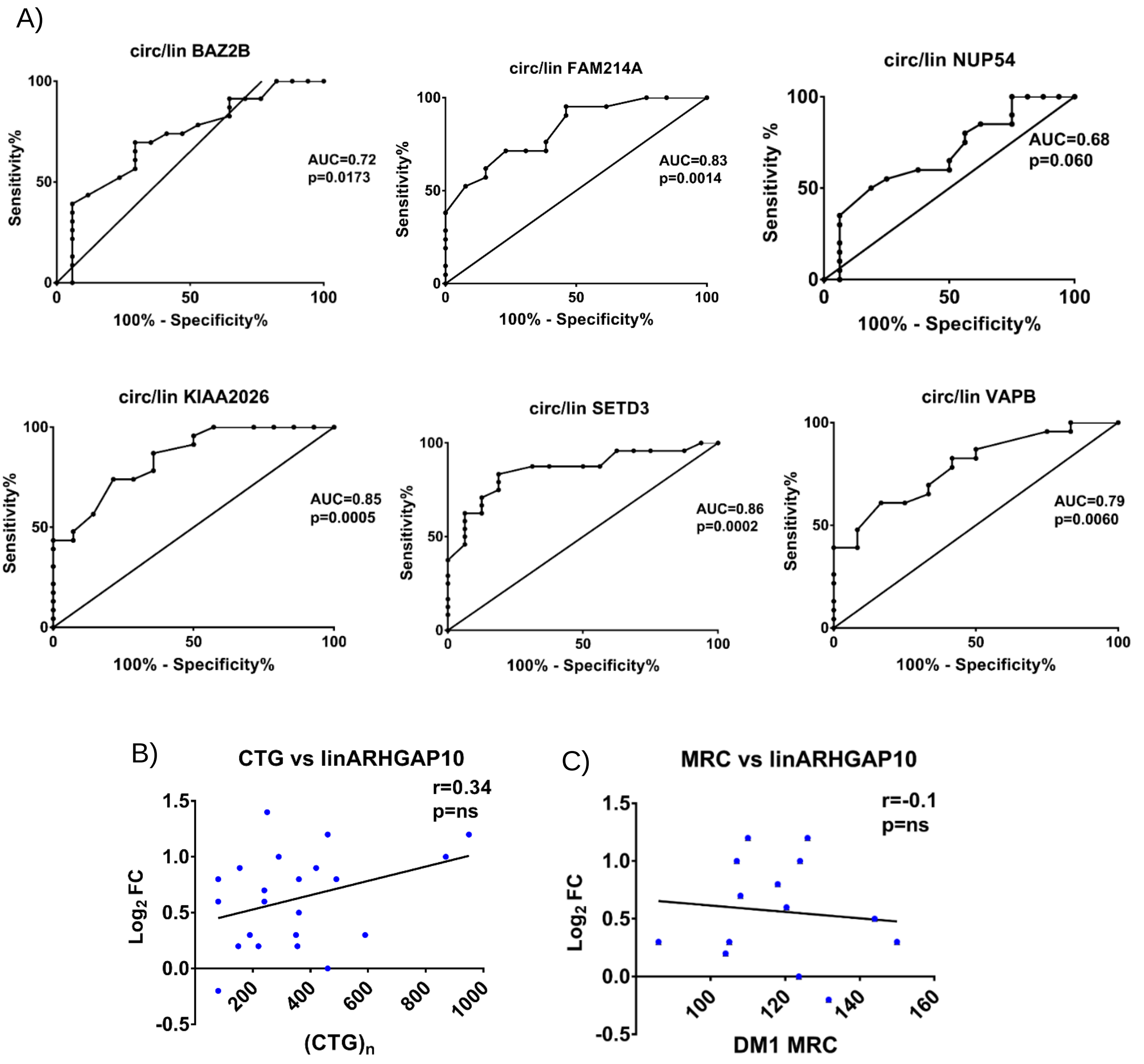

Figure S5. Discrimination of DM1 patients from healthy controls using circ/lin RNA expression ratios of DM1-circRNA candidates.

(A) Receiver operating characteristic (ROC) curve showing the sensitivity and specificity of the circRNA/linear RNA (circ/lin) expression ratios of selected DM1-circRNA candidates in distinguishing DM1 muscle biopsies from healthy controls.(B-C) Pearson correlation analysis between linARHGAP10 expression levels in biceps brachii muscle biopsies and (B) CTG repeat size, and (C) skeletal muscle strength measured by the Medical Research Council (MRC) megascore. Each data point represents an individual DM1 patient sample (n = 24, shown in blue).

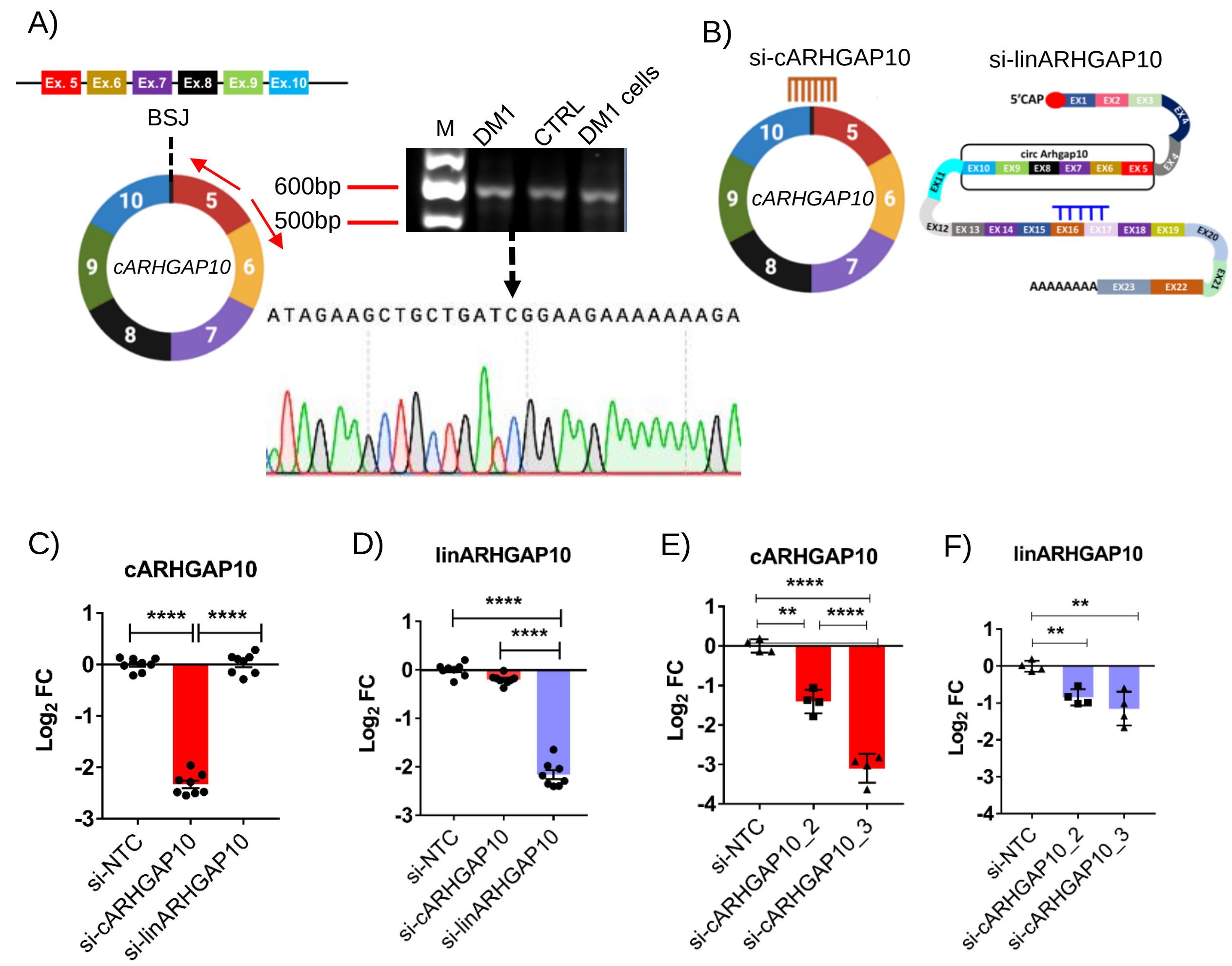

Figure S6. circARHGAP10 sequence validation and specific silencing.
(A) Diagram illustrating circARHGAP10 structure, showing the junction site between exon 10 and exon 5. The complete sequence was confirmed by Sanger sequencing of the amplicon generated using the divergent primers shown in red. (B) Design of siRNAs targeting the back-splice junction (si-cARHGAP10) to knock down circARHGAP10, and siRNAs targeting exons not involved in circularization (si-linARHGAP10) to knock down the linear isoform. (C-D) Barplots showing qPCR–measured expression levels of circARHGAP10 (C) and linARHGAP10 (D) in DM1 myogenic cells transfected with isoform-specific siRNAs or a non-targeting control (si-NTC). (E–F) Expression levels of circARHGAP10 (E) and linARHGAP10 (F) after transfection with additional siRNAs (si-cARHGAP10_2 and si-cARHGAP10_3) targeting the back-splice junction. These siRNAs showed strong knockdown efficiency but reduced isoform specificity compared to those in C–D. Data are presented as mean ± SEM on a $\log_2$ scale ($n = 8$ for C–D; $n = 4$ for E–F;), analyzed by one-way ANOVA with Tukey's post hoc test ($^{**}p < 0.01$, $^{****}p < 0.0001$).

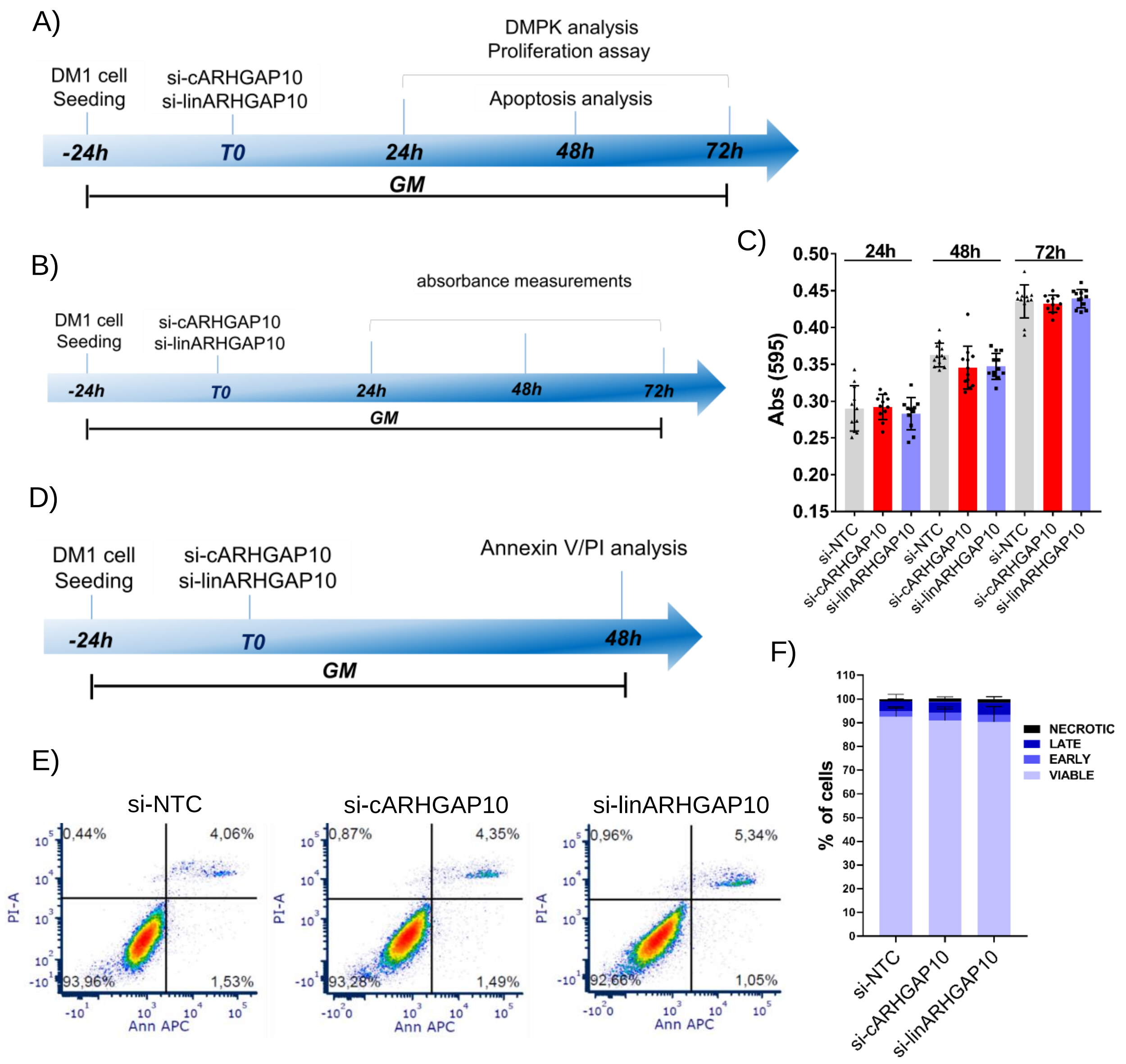

Figure S7. Silencing of circARHGAP10 and linARHGAP10 does not affect DM1 cell proliferation and apoptosis.

(A) Schematic of the siRNA transfection protocol in DM1 myogenic cells cultured in growth medium (GM). (B) Proliferation timeline using crystal violet assay. Cells were transfected with siRNAs targeting circARHGAP10 (si-cARHGAP10), linARHGAP10 (si-linARHGAP10), or a non-targeting control (si-NTC), and cultured in GM. (C) Cell proliferation was assessed at 24, 48, and 72 hours post-transfection by measuring absorbance at 595 nm. No statistically significant differences were observed. Data are presented as mean ± SEM (n = 11), analyzed by one-way ANOVA with Tukey's post hoc test, comparing each group to si-NTC at each time point. (D-E) Apoptosis timeline assessed 48 hours post-transfection using annexin V-APC/PI staining and representative FACS plots. (F) Bar graphs showing the percentages of viable, early apoptotic, late apoptotic, and necrotic cells. No significant differences were observed between groups. Data are presented as mean ± SEM (n = 6), analyzed by two-way ANOVA with Dunnett's post hoc test, comparing each group to si-NTC.

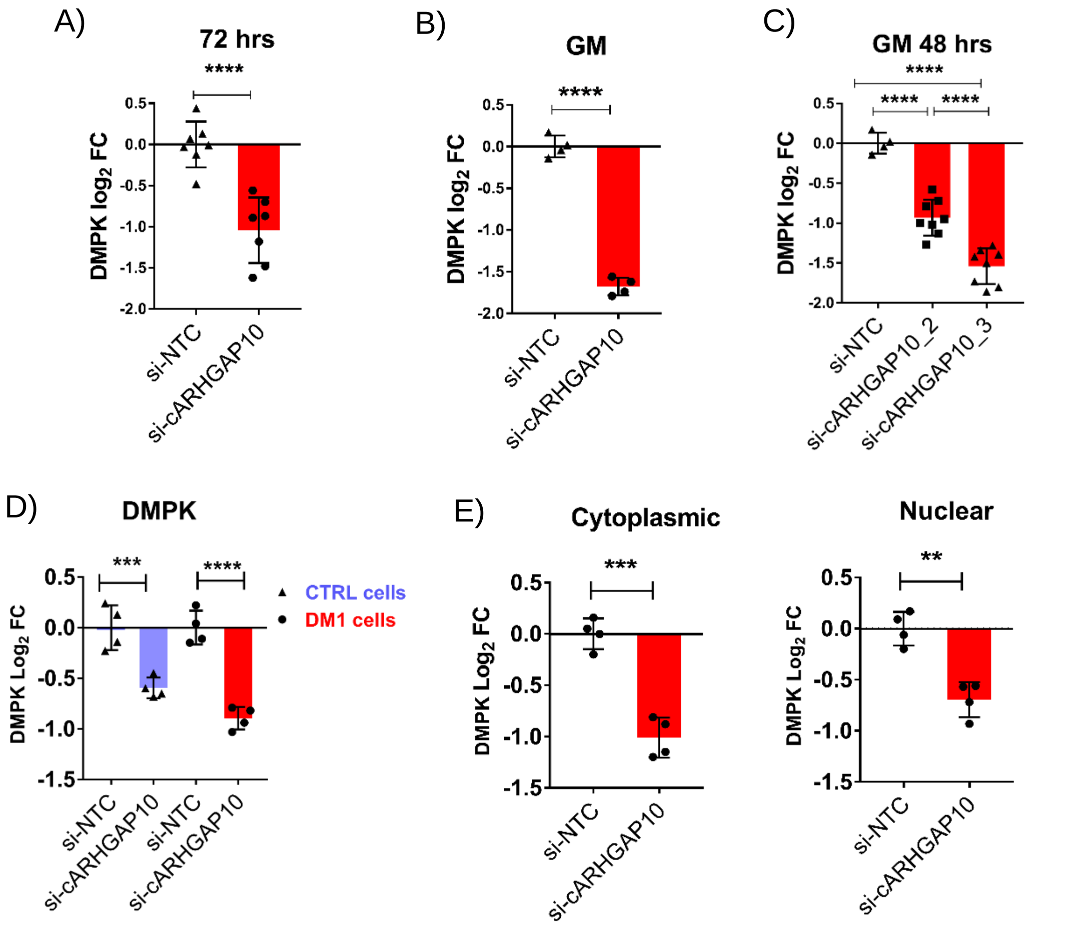

Figure S8. circARHGAP10 silencing decreases DMPK expression.

(A–E) Barplots showing DMPK expression levels measured by qPCR in DM1 myogenic cells transfected with siRNAs targeting circARHGAP10 (si-cARHGAP10) or non-targeting control siRNAs (si-NTC). (A) DMPK levels measured after 72 h in differentiation medium (n = 7; two-tailed unpaired t-test). (B) DMPK levels measured in growth medium (GM) (n = 4; two-tailed unpaired t-test). (C) DMPK expression in DM1 myogenic cells transfected with two independent siRNAs targeting circARHGAP10 (si-cARHGAP10_2 and si-cARHGAP10_3; n = 8; one-way ANOVA with Tukey’s post hoc test). (D) DMPK expression in differentiated control (CTRL, blue) and DM1 (red) myogenic cells transfected with si-cARHGAP10 or si-NTC (n = 4; one-way ANOVA with Tukey’s post hoc test). (E) DMPK expression in cytoplasmic and nuclear fractions of DM1 myogenic cells transfected with si-cARHGAP10 or si-NTC (n = 4; two-tailed unpaired t-test) ,($^{**}p < 0.01$, $^{***}p < 0.001$, $^{****}p < 0.0001$).

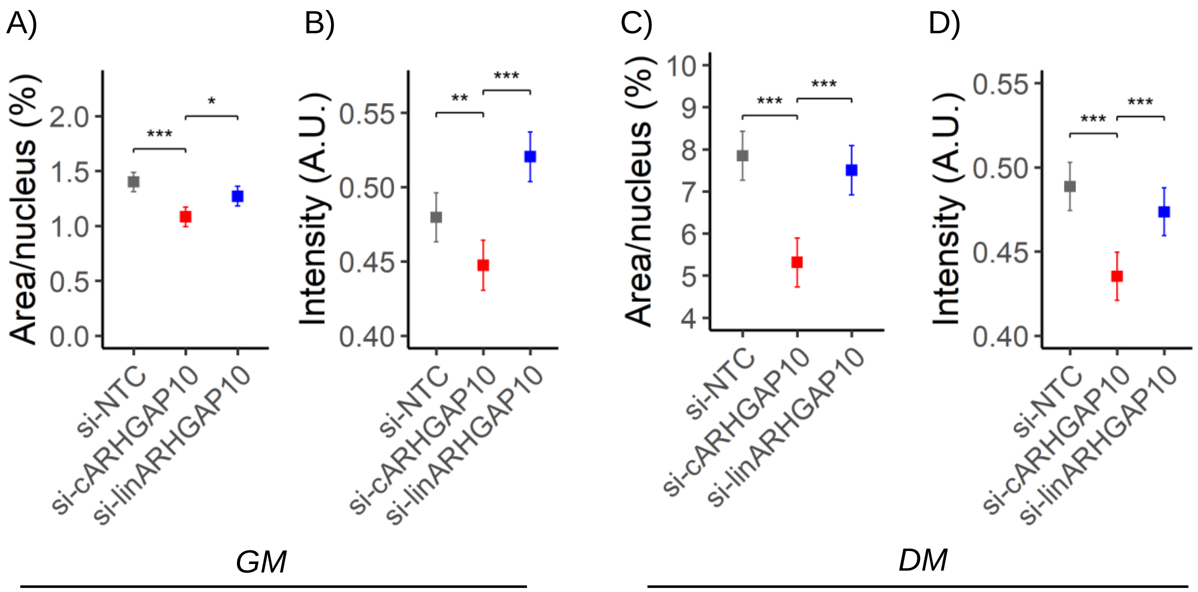

Figure S9. Silencing of circARHGAP10 reduces both the size and intensity of CUG foci. DM1 cells were transfected with siRNAs targeting circARHGAP10 (si-cARHGAP10_1), linARHGAP10 (si-linARHGAP10), or non-targeting control siRNAs (si-NTC), and cultured in either growth medium (GM) or differentiation medium (DM) for 48 h prior to RNA FISH analysis. Linear mixed-effects models were used to compare the percentage of nuclear area occupied by CUG foci (A, C) and the mean fluorescence intensity per focus (arbitrary units, A.U.) (B, D) across treatment conditions. Quantification was performed in DM1 cells cultured in GM (A–B; n = 5) and DM (C–D; n = 4) from independent experiments. Data points represent model-derived estimated marginal means. Error bars indicate SEM. Statistical significance was determined using linear mixed-effects models with Satterthwaite's method for degrees of freedom and Tukey-adjusted post hoc pairwise comparisons (*$p < 0.05$, **$p < 0.01$, ***$p < 0.001$).

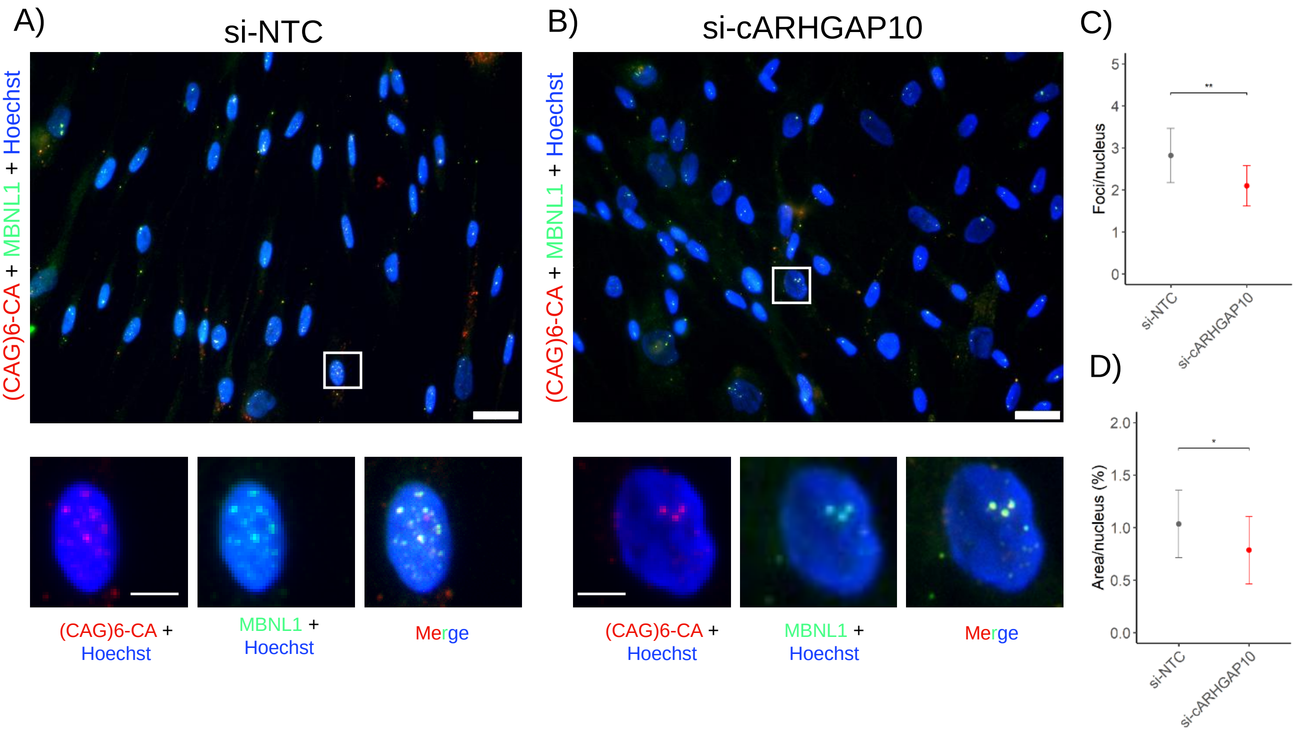

Figure S10. Silencing of circARHGAP10 reduces MBNL1 sequestration in differentiated DM1 myogenic cells.

(A–B) Representative images of RNA FISH combined with MBNL1 immunofluorescence showing nuclear CTG-CUG RNA foci and MBNL1 localization in DM1 cells cultured in differentiation medium for 48 hours and transfected with non-targeting siRNAs (si-NTC, A) or siRNAs targeting circARHGAP10 (si-cARHGAP10, B). CTG-CUG RNA foci are shown in red, MBNL1 in green, and nuclei in blue (Hoechst). Top panels show full optical fields; bottom panels display magnified views of the boxed regions highlighting individual nuclei (red-blue, green-blue, red-green-blue channels). Scale bars: 20 µm (overview), 5 µm (magnified views). (C–D) Quantification of MBNL1 sequestration into foci: (C) average number of MBNL1-positive foci per nucleus; (D) average percentage of nuclear area occupied by MBNL1-positive foci. Data are shown as mean ± SEM. Statistical significance was assessed using (C) generalized linear mixed-effects models (negative binomial) with Wald z-tests for foci per nucleus, and (D) with t-test with Satterthwaite's method for degrees of freedom estimation for area percentage (*$p < 0.05$, **$p < 0.01$).

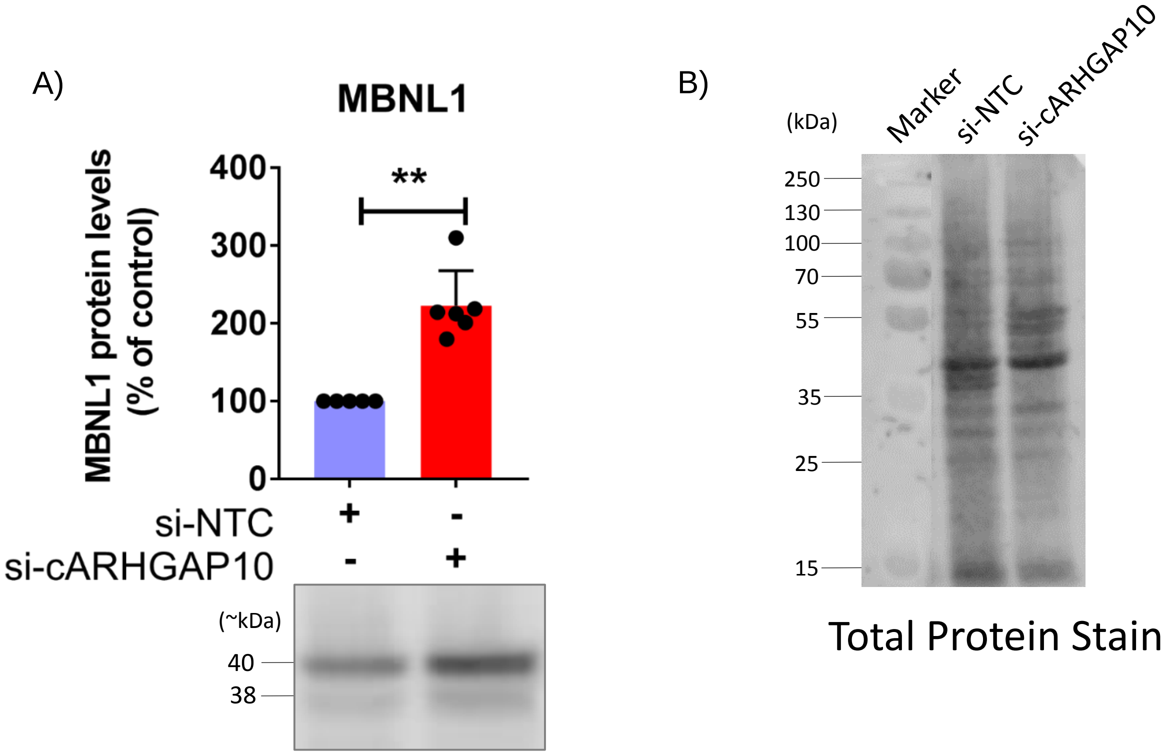

Figure S11. Increased MBNL1 protein levels in DM1 myogenic cells following circARHGAP10 silencing.
DM1 myogenic cells were transfected with siRNAs targeting circARHGAP10 (si-cARHGAP10) or non-targeting control siRNA (si-NTC) and cultured in differentiation medium (DM) for 48 hours. (A) Western blot analysis of MBNL1 protein expression. (B) Representative image of total protein staining used for normalization, performed using Revert™ Total Protein Stain (LI-COR) prior to immunodetection. Densitometric quantification of MBNL1 levels was normalized to total protein and expressed as a percentage relative to si-NTC-treated cells. Results are presented as mean ± SEM (n = 5). Statistical significance was determined using the Mann–Whitney U test (**p < 0.01)

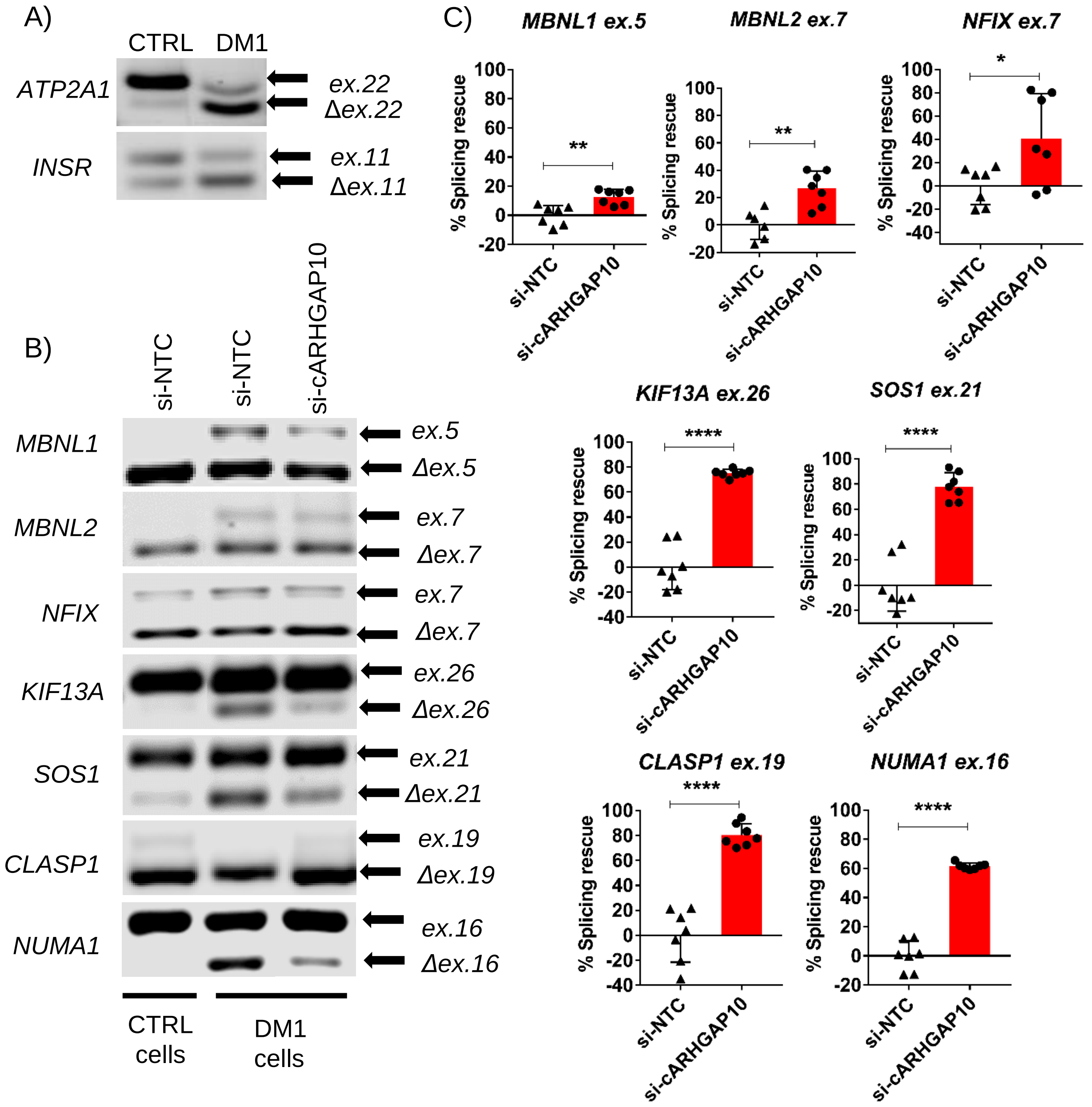

Figure S12. Partial rescue of MBNL1-regulated alternative splicing targets following circARHGAP10 silencing

(A) Representative agarose gel electrophoresis of RT-PCR products showing the alternative splicing of ATP2A1 exon 22 and INSR exon 11 in differentated control and DM1 myogenic cell cultures. (B) Agarose gel electrophoresis of RT-PCR products illustrating the splicing patterns of known MBNL1-regulated targets (MBNL1, MBNL2, NFIX, KIF13A, SOS1, CLASP1, and NUMA1) in differentiated control and DM1 myogenic cells transfected with siRNAs targeting circARHGAP10 (si-cARHGAP10) or non-targeting control siRNA (si-NTC). (C) Barplots showing quantification of splicing rescue (% Splicing Rescue) in DM1 cells treated with si-cARHGAP10 compared to si-NTC (n = 7). Percentage of splicing rescue was assessed after 36 hours of differentiation. Data are presented as mean ± SEM. Statistical significance was assessed using a two-tailed unpaired t-test (*$p < 0.05$, **$p < 0.01$, ****$p < 0.0001$).

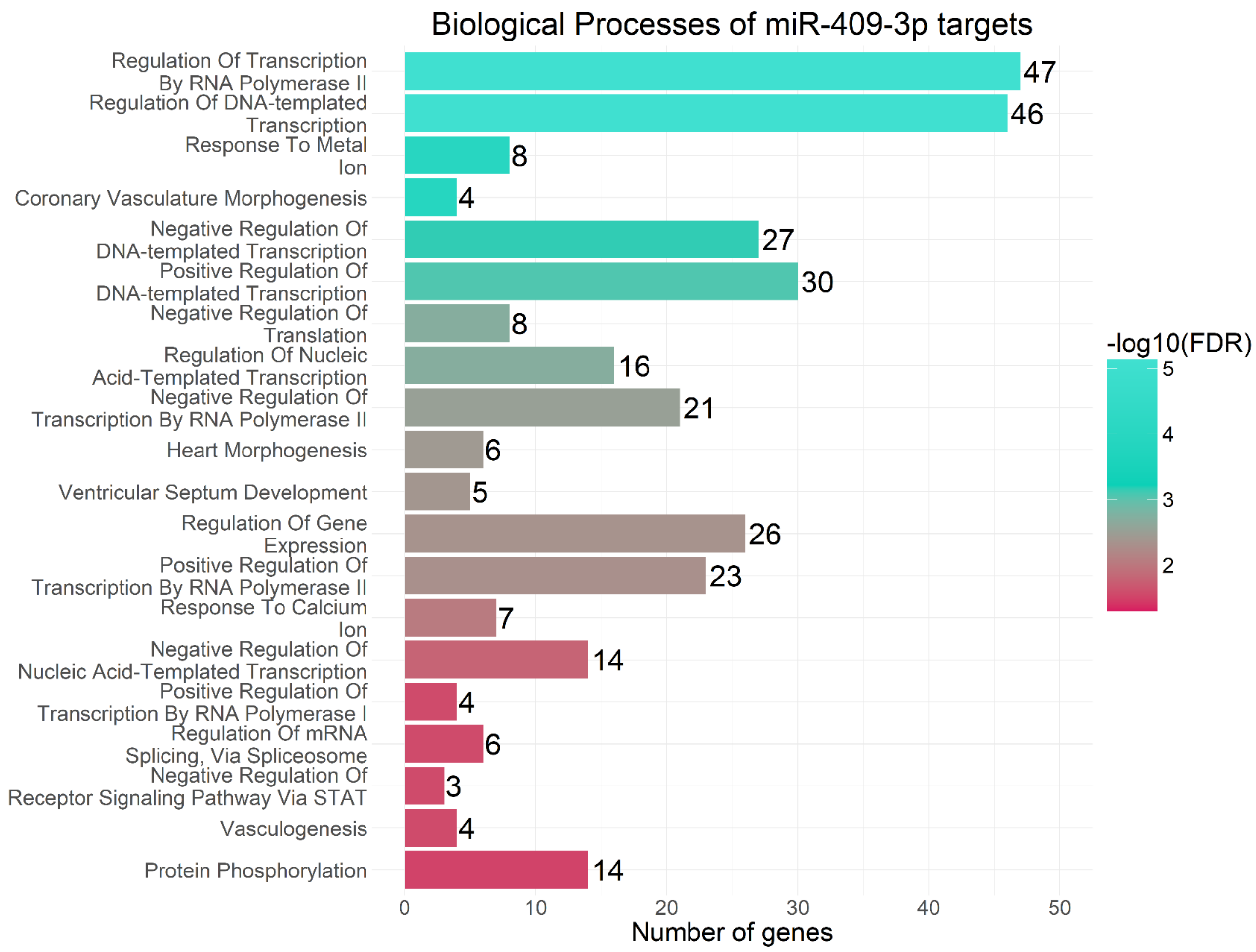

Figure S13. Analysis of Enriched Gene Ontology Biological Processes by miR-409-3p targets.

Results of enriched Gene Ontology Biological Processes analysis (top 20 significant terms) using EnrichR, based on miR-409-3p targets (TarBase v9.0, n=194). Color intensity indicates FDR values (range: [7.46×10^{-6}, 0.027]), with numbers on the bars representing the number of targets within each term.

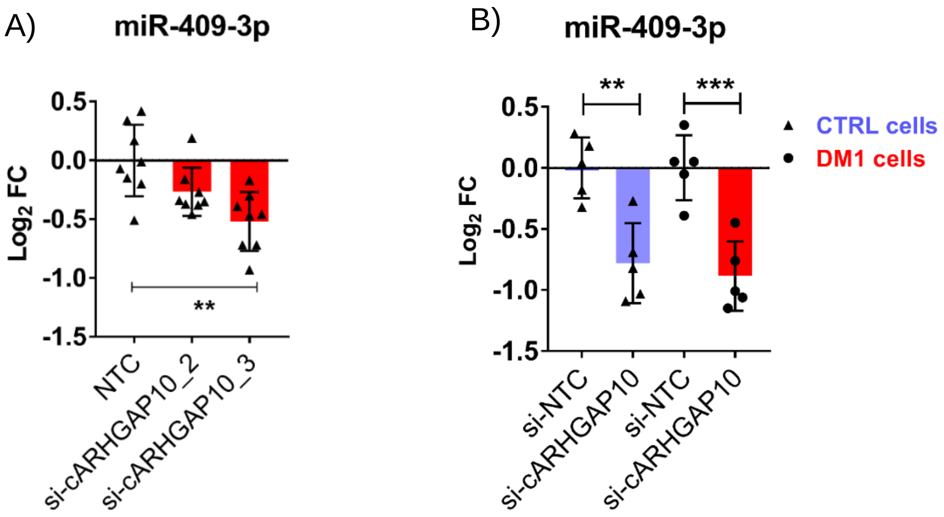

Figure S14. miR-409-3p is downregulated upon circARHGAP10 silencing.
(A) circARHGAP10 silencing downregulates miR-409-3p expression in DM1 myogenic cells. Barplots showing miR-409-3p expression levels (log_2 scale) measured by qPCR in DM1 myogenic cells transfected with two independent siRNAs targeting circARHGAP10 (si-cARHGAP10_2 and si-cARHGAP10_3). Data are presented as mean ± SEM (n = 8). (B) miR-409-3p expression in non-DM1 control (CTRL, blue) and DM1 (red) myogenic cells transfected with si-cARHGAP10 or non-targeting control (si-NTC). Data are presented in a log_2 scale as mean ± SEM (n = 4). Statistical analysis was performed using one-way ANOVA followed by Tukey's post hoc test, (**p < 0.01, ***p < 0.001).

Supplemental Tables:

Table S1. circRNA DM1 signature.

Table S2. Clinical characteristics of the patients recruited for the study.

Table S3. Sequences of qPCR primers and siRNAs.

Table S4. Annotation of TarBase targets of 409-3p regulating transcription.
