# Supplementary material for: circARHGAP10 as a candidate biomarker and therapeutic target in myotonic dystrophy type 1
Source: Mol Ther Nucleic Acids. 2025 Jul 30;36(3):102646. doi: 10.1016/j.omtn.2025.102646 (PMC12395532; doi:10.1016/j.omtn.2025.102646)
Supplement: Document S2. Article plus supplemental information [file mmc3.pdf]

Molecular Therapy
Nucleic Acids
Original Article

circARHGAP10 as a candidate biomarker and therapeutic target in myotonic dystrophy type 1

Denisa Baci,[1,2] Spyros Tastsoglou,[1] Claudia Provenzano,[3] Alessandra Perfetti,[1] Mariapaola Izzo,[3] Mario Lisanti,[1] Svetlana Frolova,[1,4] Christine Voellenkle,[1] Anna Sofia Tascini,[5] Rosanna Cardani,[6] Beatrice Cardinali,[3] Giovanni Meola,[7] Germana Falcone,[3] and Fabio Martelli[1]

[1]Molecular Cardiology Laboratory, IRCCS Policlinico San Donato, San Donato Milanese, Milan 20097, Italy; [2]Laboratory of Immunology and General Pathology, Department of Biotechnology and Life Sciences, University of Insubria, 21100 Varese, Italy; [3]Institute of Biochemistry and Cell Biology, National Research Council, Monterotondo, Rome 00015, Italy; [4]Department of Biosciences, University of Milan, 20122 Milan, Italy; [5]Center for Omics Sciences, IRCCS Ospedale San Raffaele, 20132 Milan, Italy; [6]BioCor Biobank, IRCCS Policlinico San Donato, San Donato Milanese, Milan 20097, Italy; [7]Department of Neurorehabilitation Sciences, Casa di Cura Igea, Department of Biomedical Sciences for Health, University of Milan, 20122 Milan, Italy

Myotonic dystrophy type 1 (DM1) is a multisystemic disorder caused by expanded CTG repeats in the 3′-UTR of the *DMPK* gene that lead to nuclear foci accumulation and splicing defects. Circular RNAs (circRNAs) are emerging regulators of muscular disorders, but their role in DM1 remains largely unknown. By analyzing available RNA-sequencing datasets from DM1 patients, followed by validation in patients and matching control muscle biopsies, we identified seven circRNAs that were significantly increased in DM1 muscles and displayed high circular-to-linear isoform ratios. Among them, circARHGAP10 correlated positively with CTG repeat length and inversely with muscle strength, indicating its potential as a biomarker. Silencing of circARHGAP10 in DM1 myogenic cells reduced *DMPK* expression, decreased nuclear foci, and partially rescued normal splicing. Bioinformatics prediction and pull-down of circARHGAP10 indicated that circARHGAP10 binds miR-409-3p. circARHGAP10 and miR-409-3p were both found to be upregulated in DM1 muscle biopsies and silencing of circARHGAP10 led to the downregulation of miR-409-3p, indicating their co-regulation. Interestingly, miR-409-3p overexpression blocked the beneficial effects of circARHGAP10 silencing on *DMPK* levels, foci, and splicing. Thus, circARHGAP10-dependent regulation of DM1-associated mechanisms is mediated, at least in part, via interaction with miR-409-3p. In conclusion, circARHGAP10 exhibits promising potential as a biomarker and therapeutic target for DM1.

INTRODUCTION

Myotonic dystrophy type 1 (DM1) is an autosomal dominant multisystemic disorder representing the most common dystrophy in adults, characterized by progressive muscle wasting and weakness, heart conduction defects, cataracts, insulin resistance, and cognitive defects.[1,2] Muscular and cardiac manifestations lead to reduced lifespan and quality of life; thus, development of DM1 therapies represents an important unmet medical need. In addition, the slow progression of DM1 manifestations necessitates the identification of sensitive prognostic or monitoring biomarkers, which is another relevant issue that needs to be addressed also for tracking outcomes during clinical trials.

DM1 is caused by an expansion of unstable CTG repeats within the 3′-untranslated region (UTR) of the *myotonic dystrophy protein kinase* (*DMPK*) gene.[3,4] The number of expansions correlates with the severity of symptoms and the age of onset.[5] Expression of the mutated *DMPK* gene leads to the production of toxic RNA that accumulates in distinctive nuclear foci resulting in sequestration of MBNL-family proteins and upregulation of CUGBP1.[6–8] Misregulation of these RNA-binding proteins accounts for the major splicing perturbations exhibited in DM1, which is often referred to as a "spliceopathy".[8–13] In addition to alternative splicing dysregulation, CUG repeats disrupt other cellular processes, including microRNA expression and mRNA translation, as well as alterations of RNA export and clearance mechanisms and of the nonsense-mediated mRNA decay (NMD) pathway.[11,14–17]

Circular RNAs (circRNAs) are non-coding RNAs that assume a covalently closed loop structure via a back-splicing process of maturing pre-mRNAs, in which the 5′- and 3′-termini are covalently linked to form closed RNA species.[18,19] With the rapid development of high-throughput sequencing technologies and tailored

Received 31 October 2024; accepted 25 July 2025;
https://doi.org/10.1016/j.omtn.2025.102646.

Correspondence: Germana Falcone, Via E. Ramarini 32, 00015 Monterotondo Scalo, Rome, Italy.
E-mail: germana.falcone@cnr.it
Correspondence: Fabio Martelli, Via Olgettina 58, 20132 Milan, Italy.
E-mail: fabio.martelli@grupposandonato.it

© 2025 The Authors. Published by Elsevier Inc. on behalf of The American Society of Gene and Cell Therapy. This is an open access article under the CC BY license (http://creativecommons.org/licenses/by/4.0/).

bioinformatics algorithms, thousands of circRNAs have been identified, thus creating a novel hotspot within the field of RNA research.[19–21] CircRNAs feature neither a 3′, poly-A tail nor a 5′-cap structure, yet are more stable and resistant to ribonuclease digestion than linear RNAs. Notably, they display tissue- and context-specific expression patterns, and thus can potentially represent a new class of biomarkers.[19,22,23] circRNAs can scaffold proteins, recruit other RNA species, and through sponging of miRNAs, can affect the transcriptional silencing, translation, and decay of specific mRNAs.[19,23–25]

Since DM1 is mainly a spliceopathy, circRNA dysregulation has been investigated in this context. Indeed, we have previously identified a small subset of circRNAs that are significantly increased in muscle biopsies of DM1 patients.[26] Consistent with our findings, other groups have also reported an increase in global circRNA levels in DM1, associated with muscle weakness and alternative splicing defects.[27–29]

Despite these advances, research on circRNAs and DM1 has been mostly restricted to providing evidence of circRNA dysregulation, while their implication in DM1 pathomechanisms remains largely unknown. To date, circRNAs associated with DM1 have not been functionally or mechanistically characterized.

In the present study, to identify DM1-associated circRNAs (DM1-circRNAs), we profiled the circRNA landscape of DM1 skeletal muscle by re-analyzing RNA-sequencing datasets and performing qPCR validation in muscle biopsies from independent patients. We identified and characterized new circRNAs that are dysregulated in DM1 and assessed their involvement in DM1 molecular mechanisms. Specifically, we demonstrated that targeting circARHGAP10 leads to a significant downregulation of *DMPK* expression, decreased nuclear foci, and rescue of splicing defects in DM1 myogenic cells.

RESULTS

The global increase of circRNA levels in DM1

In line with the global increase of circRNA previously reported by Czubak et al.,[29] and to further support this observation, we analyzed in more detail the DM1 and control datasets previously studied in Voellenkle et al. (GSE86356 dataset).[26] Analysis assisted by the CIRIquant circRNA quantification tool indicated a global increase of circRNAs in DM1 patients, but not of their linear counterparts, both in *tibialis anterior* muscles (Figures 1A and 1B) and in *quadriceps* muscles (Figures 1C and 1D). Similar results were obtained by analyzing both expressed circRNAs “all,” and “common” circRNAs, i.e., those expressed across all samples or in all samples but one (Figure 1).

To determine whether circRNA dysregulation is a consistent feature of DM1, we performed re-analysis and circRNA quantification of several distinct publicly available RNA-sequencing datasets. Notably, a similar pattern was also obtained when the total-RNA-sequencing dataset by Hale et al. was analyzed.[30] This independent dataset (GSE201255) features samples of a variety of muscles from adults affected by DM1 and controls, as well as from patients affected by the most severe form of DM1, early-onset congenital myotonic dystrophy (CDM), and respective pediatric controls. Despite the limitation that muscle types were not the same across conditions, the same global increase of circRNAs was observed in both adult DM1 samples (Figures S1A and S1B) and pediatric CDM samples (Figures S1C and S1D). To assess whether the upregulation of circRNAs observed in DM1 samples is a condition-specific phenomenon, rather than a common feature across myopathies, we also analyzed datasets from patients with other myopathic conditions. In contrast, the global increase of circRNAs was not observed in sarcopenia *vastus lateralis* samples compared with matched controls from two cohorts (GSE111016 in Singapore and GSE111010 in Jamaica) (Figures S1E–S1H),[31] nor in *semimembranosus* muscle samples from limb-girdle muscular dystrophy R12 (LGMD-R12) compared with respective controls (GSE202745)[32] (Figures S1I and S1J). These findings indicate that global circRNA dysregulation is a frequent occurrence in skeletal muscles of DM1 patients but is not a general feature of all myopathies or muscle-wasting syndromes.

Identification of novel DM1-associated circRNAs

For the identification of DM1-circRNAs, stringent filtering criteria were applied to single out circRNAs exhibiting significantly modulated levels in DM1-affected skeletal muscles and in independent studies. To this end, we took advantage of previous analyses performed in *tibialis anterior* muscles by our group in Voellenkle et al.[26] and by Czubak and colleagues.[29] Of note, both studies re-analyzed published DM1 RNA-sequencing datasets,[33] but did not investigate the same samples and adopted different analysis criteria. We identified 21 candidate circRNAs that displayed a high circular-to-linear ratio (circ/lin ratio) and were significantly modulated in DM1 samples compared with controls (Table S1).

To further validate this signature, muscle tissue biopsies were harvested from *biceps brachii* of 24 DM1 and 16 sex- and age-matched control individuals with no signs of neuromuscular disorders (Table S2). Most DM1 patients were at stage 3–4[34] and the pathological expansions of the CTG triplets ranged from 90 to 900. Total RNAs were isolated and the expression of circRNAs and their linear counterparts was measured by qPCR. Out of the 21 circRNA candidates, 20 primer pairs (identifying circular and linear counterparts) of 10 candidates passed all technical checks of specificity and efficiency (Table S3). The primers designed for circRNAs generated an amplicon spanning the back-splice junction, while the primers for the linear transcripts resulted in amplicons crossing the linear junction to a neighboring exon. This allowed us to measure not only the levels of the circRNA species but also modulation of the ratios between the circular and linear isoforms (circ/lin ratio). Out of 10 circRNAs tested, nine displayed significantly increased levels in DM1 *biceps brachii* samples compared with controls (Figures 2A and S2 for individual values).

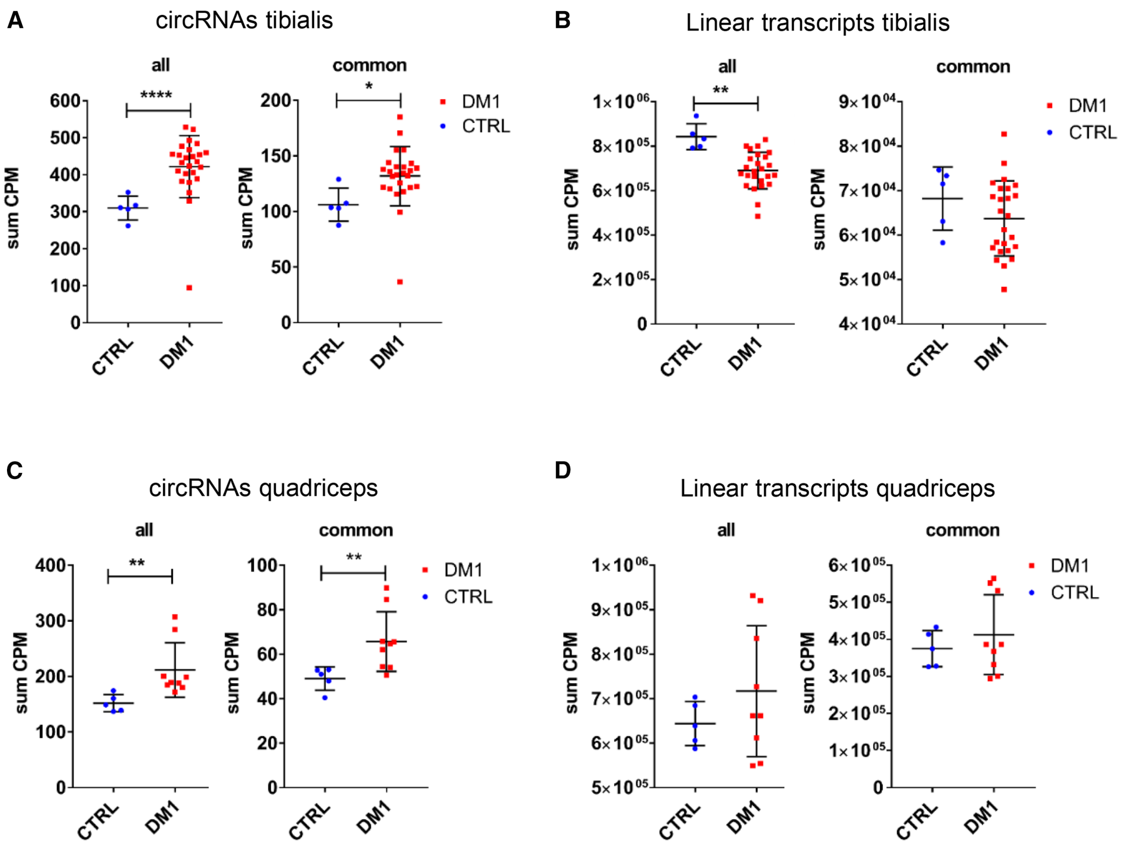

Figure 1. Global circRNA abundance is increased in DM1

Swarm plots show the sum of normalized expression values for all expressed circRNAs ("all") and circRNAs found expressed in all or all-but-one samples ("common") in tibialis anterior (A) and quadriceps (C) muscle samples of DM1 and control subjects. The corresponding linear RNA levels in tibialis anterior (B) and quadriceps (D) are also shown. Data were analyzed from the GSE86356 dataset. Each point corresponds to the sum of counts-per-million (CPM) reads mapped to the transcriptome in each sample, normalized for library depth. DM1 vs. CTRL comparisons were performed using a two-sided Welch's t test (n_tibialis_DM1 = 25, n_quadriceps_DM1 = 9, n_tibialis_CTRL = 5, n_quadriceps_CTRL = 5); **p* < 0.05, ***p* < 0.01, *****p* < 0.0001.

To assess whether the observed induction of the circRNAs was merely a consequence of a general increase in transcription of the relevant genomic regions in DM1 patients, regulation of the circ/lin ratio was tested. We identified seven new circRNAs with a significantly increased circ/lin ratio in DM1 muscles (Figures 2B and S2) indicating that upregulation of the circular transcript occurs independently from its linear counterpart. Moreover, we previously showed that both HIPK3 circRNA abundance and its circular-to-linear ratio were increased in DM1 *biceps brachii* samples compared with controls.[26] The expected back-splice junction sequences and the circularity of seven validated DM1-circRNA candidates were confirmed by Sanger sequencing and resistance to RNase R digestion (Figures S3 and S4).

Overall, this highly stringent selection pipeline allowed the identification of a bona fide DM1-circRNA signature. All candidate circRNAs that were dysregulated in *tibialis anterior* muscles of DM1 patients also displayed a positive regulation in DM1 *biceps brachii* patients (Table S1).

circARHGAP10 correlates with clinical parameters and displays a diagnostic potential in DM1 patients

Next, to evaluate if the identified DM1-circRNAs displayed a discriminatory capacity between DM1 patients and controls, receiver operating characteristic (ROC) curve analysis was performed. Specifically, among the circRNAs with significantly increased circ/lin ratios in DM1, circARHGAP10 and circSETD3 exhibited the highest ability to discriminate DM1 patients from healthy controls, each with an area under the curve (AUC) of 0.86 (Figures 3A and S5A). The AUC values of the circ/lin ratios for other candidates (circBAZ2B, circKIAA2026, circVAPB, circFAM214A, and circNUP54) ranged from 0.68 to 0.85 (Figure S5A).

To evaluate a potential relationship between the deregulation of DM1-circRNAs and clinical conditions, correlation analyses with relevant clinical parameters for DM1 patients were performed. Interestingly, circARHGAP10 expression levels in DM1 patients showed a statistically significant direct correlation with the number of CTG repeat sizes (r = 0.53) and an inverse

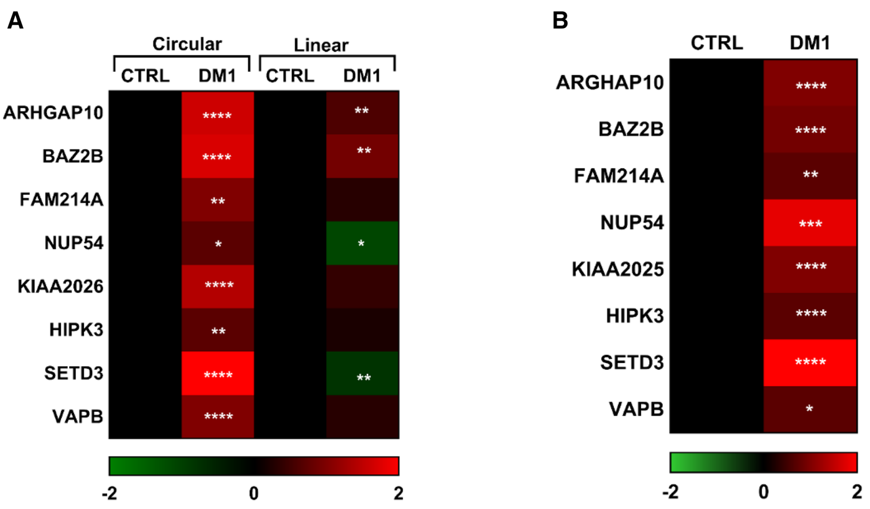

Figure 2. Validation of DM1-circRNA candidates

qPCR validation of DM1-circRNA levels and circ/lin ratios in *biceps brachii* samples from DM1 patients. Heatmaps display increased circRNA levels (A) and significantly higher circ/lin ratios (B) in DM1 compared with CTRLs. Average values are expressed on a $\log_2$ scale and where green indicates downregulation and red indicates upregulation. Statistical differences between the DM1 (n = 24) and control (n = 16) groups were assessed using the Mann-Whitney U test (*$p < 0.05$, **$p < 0.01$, ***$p < 0.001$, ****$p < 0.0001$).

correlation with skeletal muscle strength as measured by the Medical Research Council (MRC) megascore (r = −0.60) (Figures 3B and 3C). In contrast, no significant correlation was observed between linARHGAP10 expression levels and those clinical parameters, suggesting a distinct regulatory mechanism for circARHGAP10 independent of its linear counterpart (Figures S5B and S5C).

Among the other circRNA candidates displaying differential expression in DM1 compared with controls, circHIPK3 also demonstrated an inverse correlation with skeletal muscle strength, as described in our previous study (AUC = 0.83; r = −0.54). No statistically significant correlations were found for the other candidates. Thus, we focused on circARHGAP10 since it displayed promising differentiation medium (DM)-discriminating potential and correlated with important clinical parameters.

circARHGAP10 characterization in skeletal muscles

To characterize circARHGAP10 in skeletal muscles, the joining site between exon 5 and exon 10 was validated by Sanger sequencing of qPCR fragments encompassing the back-splice junction (Figure 3D) and the whole structure of circARHGAP10 was determined by using primers covering all exon junctions, providing further confirmation of its circular structure (Figure S6A). Back-splice junctions of the other DM1-associated circRNAs were also confirmed by Sanger sequencing, validating their circular structures. In addition, the circular nature of circARHGAP10 was confirmed by its resistance to RNase R digestion. While linARHGAP10 was efficiently degraded, circARHGAP10 levels remained unaffected, consistent with the expected stability of circRNAs (Figure 3E). The effectiveness of RNase R treatment was confirmed by analyzing DM1-associated circRNAs, all of which were resistant to digestion, while their linear counterparts were substantially degraded. As additional controls, circPVT1[35] and circHIPK3[36] also showed strong resistance, whereas linPVT1 and linHIPK3 were efficiently degraded, consistent with previous studies (Figures S4A and S4B). These results confirm both RNase R specificity and the circular nature of the analyzed RNAs.

Most exonic circRNAs are transported to the cytoplasm where they often act as microRNA (miRNA) sponges.[19,23,25] Nuclear/cytoplasmic fractionation experiments in DM1 myogenic cells showed that circARHGAP10 was mainly localized in the cytoplasm, while the linear form was distributed at similar levels in the cytoplasmic and nuclear fractions (Figure 3F). To validate the nucleo-cytoplasmic fractionation, the long non-coding RNA (lncRNA) *MALAT1*[37] and *RPL23* mRNA were assessed as nuclear and cytoplasmic markers, respectively. As expected, *MALAT1* was enriched in the nuclear fraction, while *RPL23* was primarily detected in the cytoplasm, confirming the fractionation protocol's efficiency (Figure 3F).

Since circARHGAP10 is upregulated in DM1 muscles, loss-of-function experiments were performed to explore its potential role in disease-related pathomechanisms. To this end, siRNAs targeting either the back-splice junction or exons present in most isoforms, but not involved in the circularization process, were used to knock down the circular and linear isoforms of ARHGAP10, respectively (Figure S6B). After transfection, DM1-myogenic cells were induced to differentiate, and the expression of both circular and linear isoforms was assessed after 72 h of differentiation. Two siRNAs specific for the circular or the linear ARHGAP10 were identified: si-circARHGAP10 decreased the levels of circARHGAP10 and did not affect its linear counterpart (Figure S6C). Conversely, si-linARHGAP10 downregulated linear ARHGAP10, but not the circular isoform (Figure S6D). Two additional siRNAs targeting circARHGAP10 were also generated, named si-cARHGAP10_2 and si-cARHGAP10_3 (Figure S6E). However, although with lower efficiency, they also inhibited linear ARHGAP10. Thus, loss-of-function experiments were performed using si-cARHGAP10 and si-linARHGAP10, while si-cARHGAP10_2 and si-cARHGAP10_3 were used only for confirmation purposes.

Silencing of circARHGAP10 decreases *DMPK* expression

To obtain evidence of circARHGAP10 involvement in relevant disease mechanisms, DM1-myogenic cells were transfected with siRNAs targeting circARHGAP10 or the linear transcript and

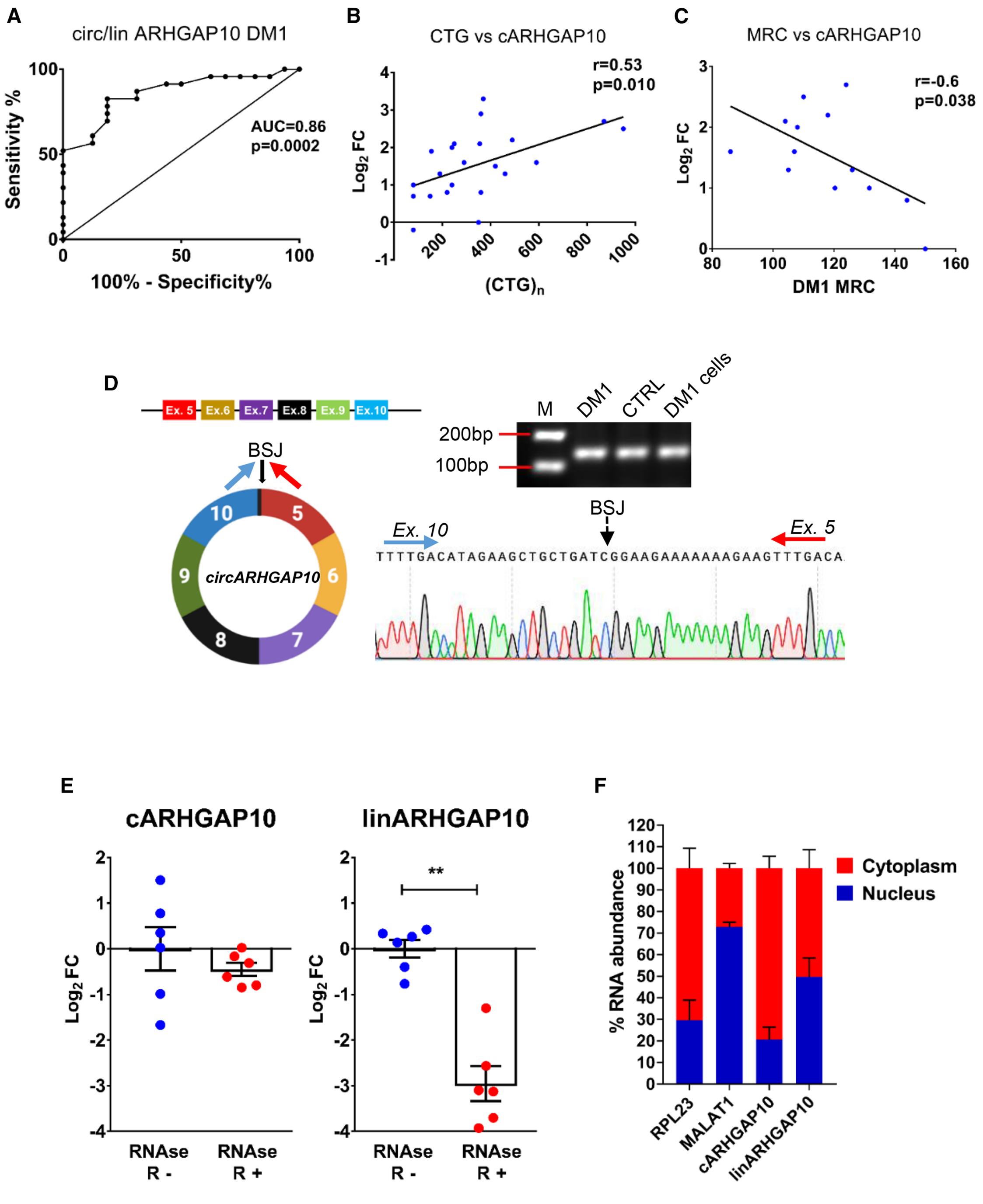

Figure 3. Correlation with clinical parameters and molecular characterization of circARHGAP10
(A) ROC curve showing the sensitivity and specificity of the circ/lin fraction of ARHGAP10 in distinguishing DM1 from healthy controls. Pearson correlations of circARHGAP10 expression with (B) CTG expansion size and (C) skeletal muscle strength (MRC) in DM1 patients. (D) Diagram of circARHGAP10 structure and junction site between exon 10 and exon 5. Sanger sequencing of the qPCR product from divergent primers covering the back-splice junction (BSJ) in control and DM1 *biceps brachii* samples. (E) qPCR analysis of circARHGAP10 (cARHGAP10) and linear ARHGAP10 transcripts (linARHGAP10) in total RNA extracted from DM1 (n = 3) and CTRL (n = 3) muscle samples, treated with (+RNase R) or without (−RNase R) RNase R exonuclease. Data are shown on a log_2 scale. Statistical significance was determined using a two-tailed Mann-Whitney U test (**p < 0.01). (F) Subcellular localization of circARHGAP10 and linARHGAP10 in cytoplasmic and nuclear RNA fractions of DM1 myogenic cells (n = 6). RPL23 and MALAT1 were used as cytoplasmic and nuclear RNA markers, respectively. Bar graphs represent relative transcript enrichment in each compartment.

analyzed at different time points after differentiation (DM) (Figure 4A) or in growth medium (GM) (Figure S7A), depending on the experimental setting and the DM1-related features analyzed.

First, the potential role of circARHGAP10 silencing on cell proliferation and apoptosis was assessed. No significant differences in cell proliferation rates of DM1 myogenic cells grown in GM were observed following silencing of ARHGAP10 circular or linear isoforms compared with transfection of non-targeting control (NTC) oligos (Figures S7B and S7C). We also observed that neither silencing of circARHGAP10 nor of linARHGAP10 triggered early or late apoptosis after 48 h, as detected by double staining with APC-conjugated Annexin V and PI using flow cytometry (Figures S7D–S7F).

DM1 is caused by an abnormal expansion of a $(CTG)_n$ trinucleotide repeat in the *DMPK* gene, and downregulation of *DMPK* mRNA expression represents a potential DM1 therapeutic avenue.[38,39] To assess the potential impact of circARHGAP10 on *DMPK* transcriptional regulation, DM1-myogenic cells were transfected with siRNAs targeting circARHGAP10 or the linear transcript, and *DMPK* expression was evaluated at different time points in proliferating as well as differentiated myogenic cells (Figures 4A and S7A). Silencing of circARHGAP10 led to a significant downregulation of *DMPK* gene expression compared with controls after 48 h, while silencing of the linear counterpart did not (Figure 4B). *DMPK* downregulation was also observed after 72 h of circARHGAP10 silencing, indicating a sustained inhibition (Figure S8A). In addition, a significant reduction of *DMPK* levels was also obtained in GM conditions, indicating that this modulation was not related to differentiation (Figure S8B). Importantly, *DMPK* reduction was detected upon transfection of two other independent siRNAs targeting the back-splice junction, confirming a specific *on*-target *effect* on *DMPK* downregulation (Figure S8C).

To determine whether circARHGAP10 knockdown preferentially affects mutant *DMPK* transcripts, we analyzed *DMPK* mRNA levels in both control and DM1 myogenic cells (Figure S8D). Following circARHGAP10 silencing, a reduction in total *DMPK* mRNA levels was observed in both DM1 and control cells, indicating that the effect is not exclusive to the mutant transcript. Additionally, nuclear and cytoplasmic RNA fractions were examined in DM1 cells (Figure S8E). This downregulation was evident in both the cytoplasmic and nuclear fractions, suggesting a global effect on *DMPK* transcript levels across cellular compartments and that *DMPK* modulation induced by circARHGAP10 silencing is unlikely to be allele-specific.

Silencing of circARHGAP10 reduces nuclear foci and increases MBNL1 availability

Transcription of $(CTG)_n$ trinucleotide repeats in the *DMPK* gene leads to the formation of ribonuclear foci, another characteristic feature of DM1 cells.[1,7] Since circARHGAP10 silencing decreased *DMPK* transcript levels, we evaluated whether this event translated into changes in CUG-foci numbers, area, and brightness. siRNAs targeting circARHGAP10 and linARHGAP10 were transfected in DM1 myogenic cells, under both proliferating (GM) and differentiated (DM) conditions and were analyzed by fluorescence *in situ* hybridization (FISH) of ribonuclear inclusions containing CUG-repeats (Figure 4C). Due to increased transcription and stability of *DMPK* mRNA upon induction of myogenic differentiation,[40] the number of foci per nucleus is usually much higher in differentiated than in proliferating cells. In both GM (Figure 4D) and DM (Figure 4E), downregulation of circARHGAP10 (but not of linARHGAP10) yielded similar effects: foci-negative nuclei were significantly increased, and the number of foci per nucleus (in foci-positive nuclei) was decreased. In addition, the nuclear area occupied by foci (measured per nucleus as total area of foci over total nuclear area, in pixels) decreased significantly upon circARHGAP10, but not linARHGAP10, downregulation in both GM and DM (Figures S9A and S9C), as did the mean brightness of foci (Figures S9B and S9D).

MBNL1 is known to be sequestered in nuclear RNA foci in DM1, leading to widespread splicing defects and contributing to disease pathogenesis.[6–8] To assess whether circARHGAP10 knockdown affects MBNL1 sequestration, we performed RNA FISH combined with MBNL1 immunofluorescence in DM1 myogenic cells cultured in DM for 48 h. Signal intensity was moderately reduced due to sequential CUG and MBNL1 staining, compared with single stainings. Nevertheless, as expected, DM1 cells transfected with control siRNAs (si-NTC) exhibited abundant MBNL1 colocalizing with nuclear CUG RNA foci, confirming its sequestration (Figure S10A). Upon silencing of circARHGAP10, the number and intensity of MBNL1-positive nuclear foci were visibly reduced (Figure S10B), suggesting decreased sequestration. Quantitative analysis using mixed-effects models revealed a significant reduction in both the average number of MBNL1-containing foci per nucleus (Figure S10C) and the percentage of nuclear area occupied by foci (Figure S10D) in si-cARHGAP10–treated cells compared with si-NTC.

To determine whether the reduced sequestration of MBNL1 following circARHGAP10 silencing was accompanied by changes in its overall cellular abundance, total MBNL1 protein levels were evaluated. This analysis was crucial to establish whether the observed increase in bioavailable MBNL1 resulted solely from reduced nuclear sequestration or may also involve broader modulation of MBNL1 expression at the post-transcriptional level.

Western blot analysis of differentiated DM1 myogenic cells transfected with si-circARHGAP10 revealed a statistically significant increase in total MBNL1 protein levels compared with si-NTC (Figure S11). These findings suggest that circARHGAP10 silencing diminishes the pathological sequestration of MBNL1 and promotes an increase in its total cellular abundance, thereby enhancing its availability.

Downregulation of circARHGAP10 partially rescues normal splicing

In DM1, expanded CTG repeats accumulating as distinctive nuclear foci dysregulate the activity of RNA splicing factors such as MBNL1

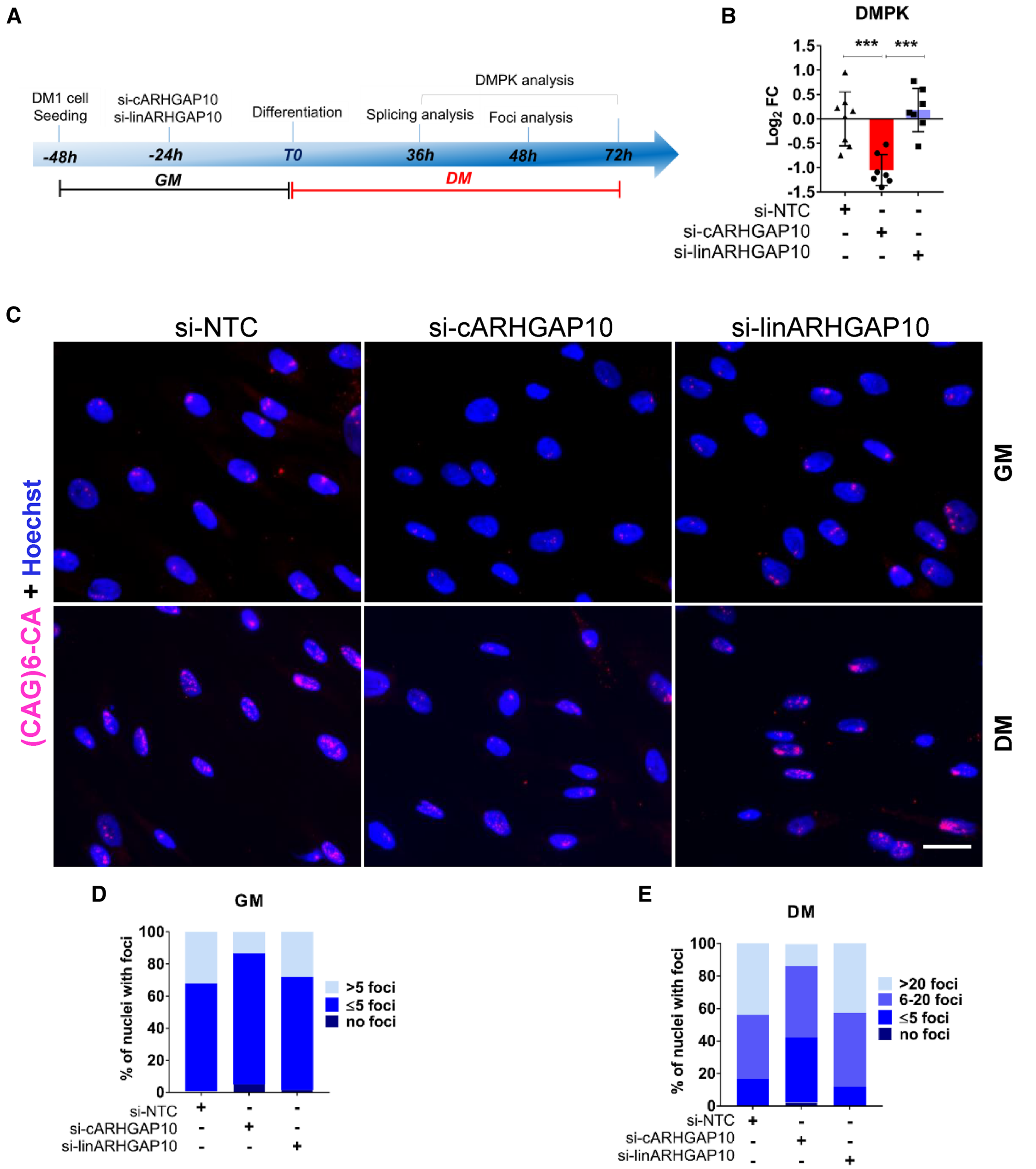

	no foci	≤5 foci	>5 foci
si-NTC	0.68 ± 0.4	67.1 ± 5.5	32.23 ± 5.5
si-cARHGAP10	5.05 ± 1.2**	81.69 ± 2.4*	13.25 ± 4.0*
si-linARHGAP10	1.64 ± 0.4	70.39 ± 4.8	27.97 ± 4.7

	no foci	≤5 foci	6-20 foci	>20 foci
si-NTC	0.1 ± 0.1	16.6 ± 2.3	39.3 ± 2.6	43.9 ± 4.8
si-cARHGAP10	2.2 ± 0.7*	40.0 ± 3.9***	43.8 ± 1.4	13.5 ± 1.6***
si-linARHGAP10	0.0 ± 0.0	11.9 ± 1.7	45.5 ± 1.2	42.6 ± 1.4

Figure 4. Silencing of circARHGAP10 reduces DMPK expression and nuclear foci in DM1 cells

(A) Experimental design for loss-of-function studies in DM1 myogenic cells cultured in differentiation medium (DM). (B) Bar plots showing DMPK levels measured by qPCR in DM1 cells transfected with siRNAs targeting circARHGAP10 (red) or linARHGAP10 (blue), or with a non-targeting control siRNA (si-NTC) in differentiation medium for 48 h. Data are presented on a $\log_2$ scale (n = 7), one-way ANOVA followed by Tukey's post hoc test (***$p < 0.001$). (C) Representative images of RNA FISH analysis showing CUG foci in DM1 cells transfected with siRNAs targeting circARHGAP10 (si-cARHGAP10), linARHGAP10, or control siRNAs (NTC). Cells were cultured in GM or DM for 48 h before analysis. Nuclear foci are shown in pink and Hoechst in blue (scale bar, 10 μm). (D and E) Bar graphs depicting the percentage of nuclei with no foci or with varying numbers of foci in in GM (n = 5) and DM (n = 4) (*$p < 0.05$, **$p < 0.01$, ***$p < 0.001$).

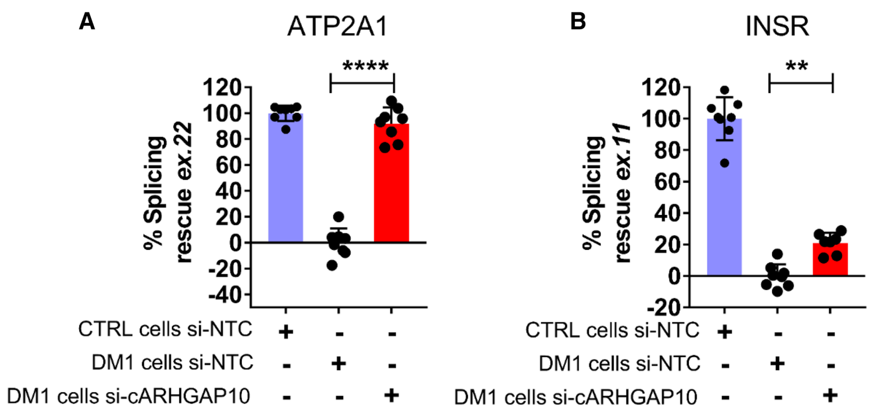

Figure 5. Silencing of circARHGAP10 partially rescues normal splicing

DM1-myogenic cells were transfected with siRNAs targeting circARHGAP10 (si-cARHGAP10) or a non-targeting control siRNA (si-NTC) and cultured in differentiation medium for 36 h. (A and B) Bar plots show the quantification of alternative splicing restoration, expressed as percent splicing rescue relative to control cells (CTRL, si-NTC). (A) Inclusion of ATP2A1 exon 22; (B) inclusion of INSR exon 11. Comparisons were made between si-cARHGAP10– and si-NTC-treated DM1 cells. Data are presented as mean ± SEM (n = 8), one-way ANOVA followed by Tukey's post hoc test (**p < 0.01, ****p < 0.0001).

and CUGBP1.[7,10] This, in turn, leads to the alteration of exon inclusion and exclusion, which constitutes a DM1 hallmark.[9,10,41] Thus, we assessed whether missplicing was affected by circARHGAP10 modulation.

First, we investigated whether sarcoplasmic/endoplasmic reticulum calcium ATPase 1 (*ATP2A1,* also known as *SERCA1*) exon 22 and insulin receptor (*INSR*) exon 11 transcripts, known to be altered in DM1, were misregulated in DM1 myogenic cells. The assay was conducted at 36 h of differentiation (Figure 4A) to maximize the difference between DM1 and wild-type control (CTRL) cells. qPCR analysis confirmed that DM1 myogenic cells displayed reduced inclusion of both exons compared with control myogenic cells (Figure S12A).

The alternative splicing patterns of *ATP2A1* and *INSR* transcripts were then assessed by qPCR analysis following circARHGAP10 silencing. Both canonical transcript forms containing exon 22 in *ATP2A1* and exon 11 in *INSR* were increased following silencing of circARHGAP10. Specifically, a significant rescue of the missplicing of both *ATP2A1* and *INSR* was obtained, with an average rescue of ∼80% for *ATP2A1* exon 22 and ∼20% for *INSR* exon 11 (Figure 5). The observed differences in rescue are likely due to the varying extent of missplicing between these transcripts. As shown in Figure S12A, exon exclusion in DM1 cells is more pronounced for the *ATP2A1* transcript compared with *INSR*.

To expand the panel of splicing events linked to MBNL1 activity and affected in DM1, we performed semi-quantitative reverse-transcription PCR (RT-PCR) using primer pairs previously validated in the literature. We analyzed splicing changes in a panel of well-characterized MBNL1-dependent targets known to be misspliced in DM1 skeletal muscle: *MBNL1* exon 5,[13] *MBNL2* exon 7,[13] *NFIX* exon 7,[13] *KIF13A* exon 26,[13] *SOS1* exon 21, and *CLASP1* exon 19[42] (Figure S12B). *NUMA1* exon 16 inclusion was also tested, as this splicing event has been linked to the bioavailability and expression of MBNL1.[43–45] Figure S12B shows distinct splicing patterns and significant differences between differentiated wild-type and DM1 myogenic cells treated with siRNAs targeting circARHGAP10 or si-NTC.

Upon circARHGAP10 silencing, we observed a statistically significant rescue for the analyzed alternative splicing events. This rescue was particularly prominent for *KIF13A* exon 26, *CLASP1* exon 19, and *SOS1* exon 21 (Figure S12C).

CircARHGAP10 interacts with miR-409-3p

One of the most prominent mechanisms of action of circRNAs is mediated by their ability to function as a sponge of specific miRNAs, in turn, regulating the expression of their target mRNAs.[23,25] By intersecting the list of miRNAs with experimentally supported binding sites on the ARHGAP10 coding sequence (DIANA-TarBase v9.0)[46] with miRNAs computationally predicted to interact with circARHGAP10 (CircInteractome),[47] we identified miR-409-3p as a high-likelihood interacting partner of circARHGAP10 (Figure 6A). Notably, the TarBase-assisted analysis identified an miR-409-3p binding site on exon 7 (Figure 6B).

By performing an over-representation analysis of all miR-409-3p targets that are supported by direct experimental methodologies in TarBase, we observed that 12 of the top 20 significant biological processes (60%) were related to regulation of transcription, splicing, and translation, while four more (20%) were related to cardiovascular morphogenesis (Figure S13). The remaining four were related to signaling pathways such as response to metal/calcium ions, protein phosphorylation, and negative regulation of receptor signaling by STAT (Figure S13). Many terms that appeared to be potentially regulated by miR-409-3p targets are relevant to the DM1 pathogenesis, prompting us to further investigate miR-409-3p in the context of DM1.

When the expression of miR-409-3p was measured in DM1 muscle biopsies, miR-409-3p expression was found to be increased in DM1 tissues (Figure 6C), suggesting a possible co-regulatory relationship between miR-409-3p and circARHGAP10. Accordingly, circARHGAP10 circ/lin ratio directly correlated with miR-409-3p levels in skeletal muscle biopsies of DM1 patients and controls

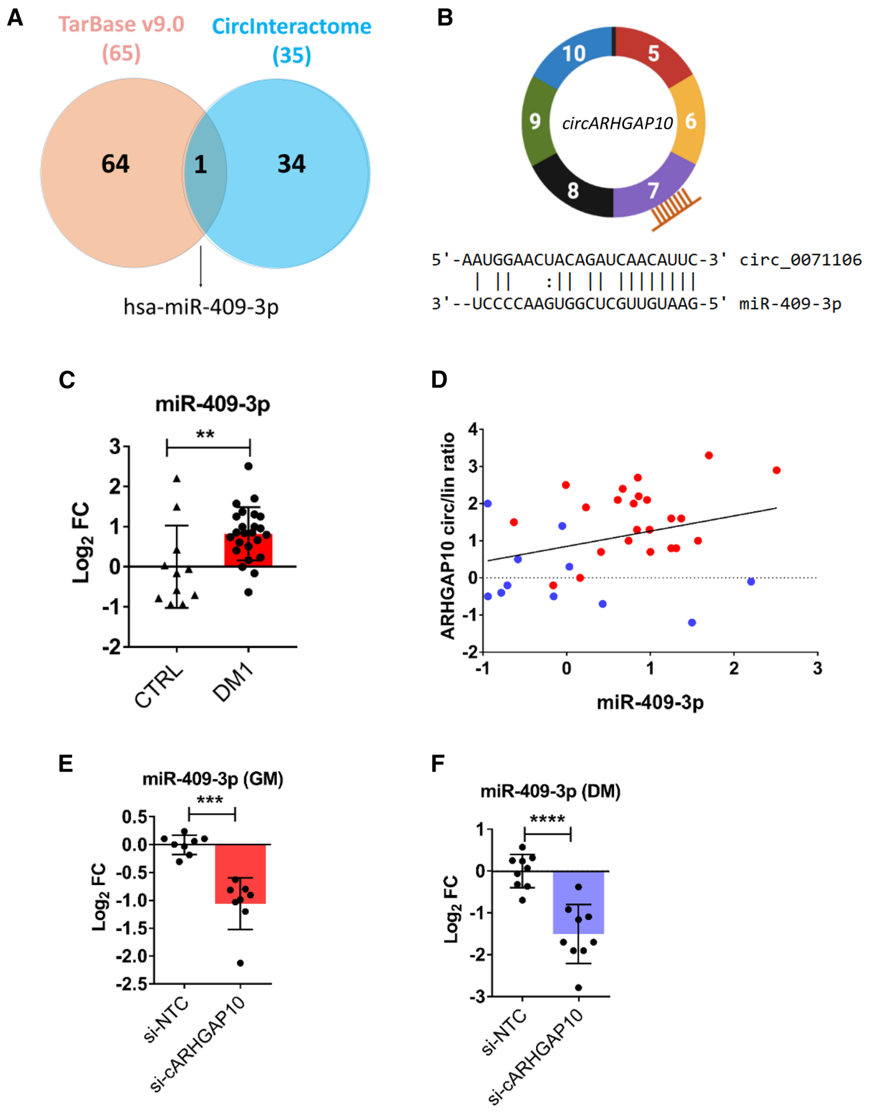

Figure 6. Functional interaction between circARHGAP10 and miR-409-3p

(A) Venn diagram showing the overlap between miRNAs targeting ARHGAP10 exons 5–10 (DIANA-TarBase v9.0) and those predicted to interact with circARHGAP10 by CircInteractome. The only shared miRNA is miR-409-3p. (B) Schematic representation of circARHGAP10 structure and the predicted binding site of miR-409-3p within exon 7. (C) Relative expression of miR-409-3p in *biceps brachii* biopsies from DM1 (n = 24) and CTRL (n = 11) shown in $\log_2$ scale, Mann-Whitney U test. (D) Pearson correlation between the circARHGAP10/linear ARHGAP10 ratio and miR-409-3p expression in DM1 and CTRL samples. (E and F) Relative expression of miR-409-3p in DM1 myogenic cells transfected with siRNAs targeting circARHGAP10 or control siRNAs, cultured in growth medium (E, GM; unpaired t test) or differentiation medium (F, DM; unpaired t test). Data are presented as mean ± SEM (n = 8) (**p < 0.01, ***p < 0.001, ****p < 0.0001).

(Figure 6D). Therefore, we next investigated the functional interaction between miR-409-3p and circARHGAP10 *in vitro*. To this end, we tested whether the expression of miR-409-3p was modulated following siRNA-mediated knockdown of circARHGAP10. miR-409-3p levels decreased following circARHGAP10 silencing in DM1 cells under both proliferating and differentiated conditions, supporting their functional interaction and co-regulation (Figure 6E). A downregulation was also observed when DM1 myogenic cells were transfected with independent siRNAs targeting the back-splice junction of circARHGAP10, confirming the co-regulation of miR-409-3p and circARHGAP10 (Figure S14A).

In addition, we assessed miR-409-3p expression following circARHGAP10 silencing in both DM1 and control myogenic cells. In both cellular contexts, miR-409-3p levels were significantly downregulated upon circARHGAP10 knockdown (Figure S14B), suggesting a shared functional regulatory interaction between circARHGAP10 and miR-409-3p in the two cell types.

To validate the predicted interaction between circARHGAP10 and miR-409-3p, an RNA pull-down assay was performed in differentiated DM1 cells using biotin-labeled antisense oligonucleotides targeting the circARHGAP10 back-splice junction sequence (bio-cARHGAP10). Both circARHGAP10 and miR-409-3p were enriched compared with the non-targeting oligonucleotide control pull-down (bio-NC), while no enrichment was found for miR-16, which was not predicted to interact with circARHGAP10 by CircInteractome (negative control, Figures 7A and 7B).

These results were confirmed by a reciprocal approach, as circARHGAP10 was enriched in pull-downs using biotinylated miR-409-3p (Figure 7C). Importantly, no enrichment in the miR-409-3p captured fraction was detected for circHIPK3, a circRNA not predicted to interact with miR-409-3p by CircInteractome (Figure 7D).

To further validate the circARHGAP10/miR-409-3p interaction, we also tested whether both RNAs interacted with the RNA-induced silencing complex (RISC), i.e., the miRNA effector complex.[48] Following immunoprecipitation of AGO2, an obligatory component of the RISC,[48] from whole-cell lysates of DM1 cells, circARHGAP10 and miR-409-3p were both enriched in the captured fraction compared with the negative control immunoprecipitate (Figure 7E).

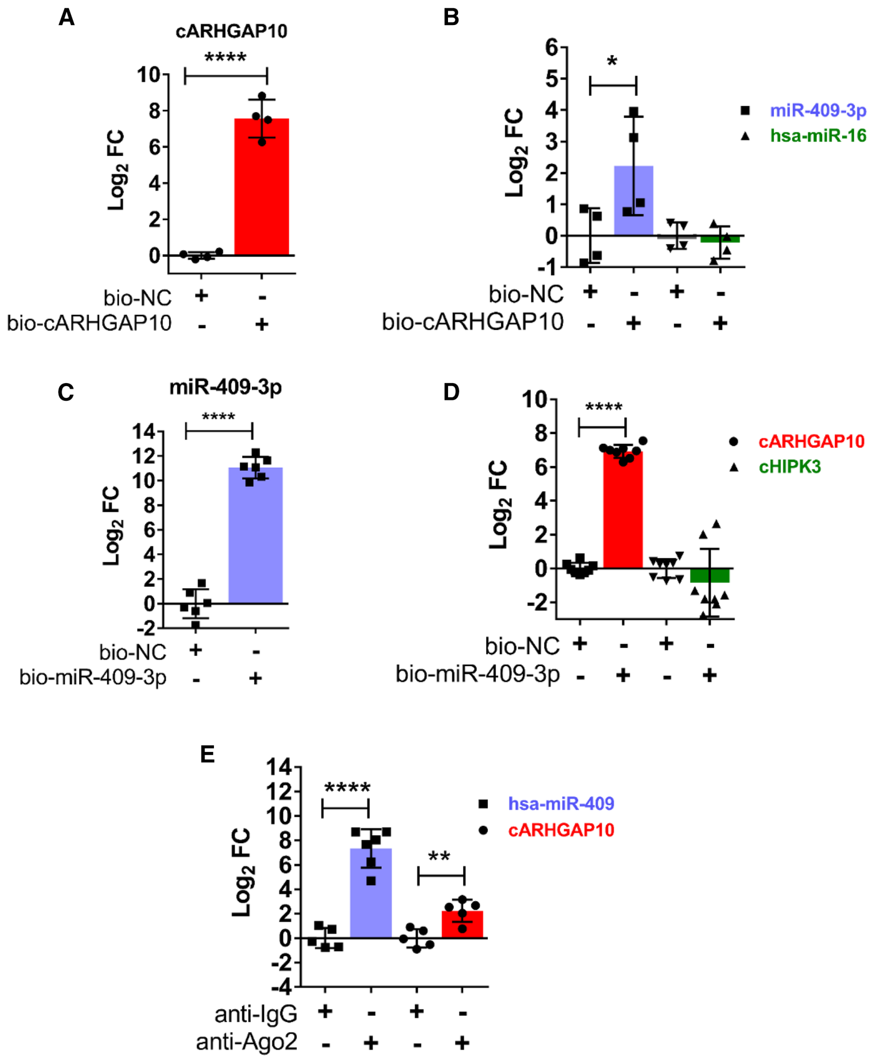

Figure 7. Physical interaction between circARHGAP10 and miR-409-3p

(A and B) circARHGAP10 pull-down analysis in DM1 myogenic cells using biotin-labeled antisense oligonucleotides targeting the circARHGAP10 back-splice junction (bio-cARHGAP10) or non-targeting control oligonucleotides (bio-NC). qPCR analysis shows enrichment of circARHGAP10 (A) and miR-409-3p (B), but not miR-16 in the bio-cARHGAP10 condition. Data are presented on a $\log_2$ scale (n = 4) unpaired two-tailed t tests vs. bio-NC (*$p < 0.05$, ****$p < 0.0001$). (C and D) Pull-down assay using biotin-labeled miR-409-3p mimic or non-targeting control oligonucleotides in DM1 cells. qPCR confirmed enrichment of miR-409-3p (C) and circARHGAP10, but not circHIPK3 (D), in the miR-409-3p pull-down (n = 6–8) unpaired two-tailed t tests vs. bio-NC (****$p < 0.0001$). (F) qPCR analysis showing enrichment of circARHGAP10 and miR-409-3p in AGO2 immunoprecipitates relative to IgG controls in DM1 cells (n = 5–6); unpaired two-tailed t tests vs. control IgG. Data are presented as mean ± SEM (**$p < 0.01$, ****$p < 0.0001$).

circARHGAP10 silencing. These data suggest that circARHGAP10 influences critical features of DM1 pathogenesis, at least in part, by interacting with miR-409-3p.

Target prediction analysis indicated several experimentally verified targets of miR-409-3p that may regulate *DMPK* expression or contribute to key pathological features of DM1. Table S4 shows potentially relevant transcription factors, chromatin modifiers, or RNA-binding proteins, involved in genomic stability, alternative splicing, and RNA processing. The interplay between circARHGAP10 and miR-409-3p may serve as a crucial modulatory network, wherein the interaction of miR-409-3p with circARHGAP10 preserves the function of these critical regulators and prevents the exacerbation of DM1 features.

miR-409-3p mediates circARHGAP10 regulation of DM1 features

After validating the interaction of miR-409-3p with circARHGAP10, we evaluated whether the impact of circARHGAP10 downregulation on DM1-related features could be attributed, at least in part, to its repressive effect on miR-409-3p. To this aim, DM1 myogenic cells were transfected with si-circARHGAP10, miR-409-3p mimic, or a combination of both, and then differentiated by culturing them in DM (Figure 8A). We then evaluated whether the downregulation of *DMPK* mRNA, the decrease in nuclear foci, and the rescue of alternative splicing induced by circARHGAP10 silencing were prevented by miR-409-3p overexpression.

Figure 8B shows that the downregulation of *DMPK* caused by circARHGAP10 silencing was significantly prevented when DM1 cells were co-transfected with the miR-409-3p mimic. Similarly, co-transfection with si-circARHGAP10 and the miR-409-3p mimic significantly reduced the rescue of nuclear foci (Figure 8C) and of alternative splicing patterns of *ATP2A1* transcripts (Figure 8D) induced by circARHGAP10 silencing.

DISCUSSION

Dysregulation of alternative splicing is a well-recognized molecular hallmark of DM1, affecting many genes associated with functions implicated in the disease mechanisms.[8–11,13] Since circRNAs, akin to linear RNAs, are splicing products, they may represent a still under-investigated source of functional biomarkers and therapeutic targets in different disorders, including DM1.[21,22,49,50]

In keeping with previous observations,[29] a global increase of circRNAs in DM1 was identified in independent sets of samples derived from different skeletal muscles and from patients affected by adult and congenital DM1. An upregulation of circRNAs, independent of parental gene expression, was also recently observed in the frontal

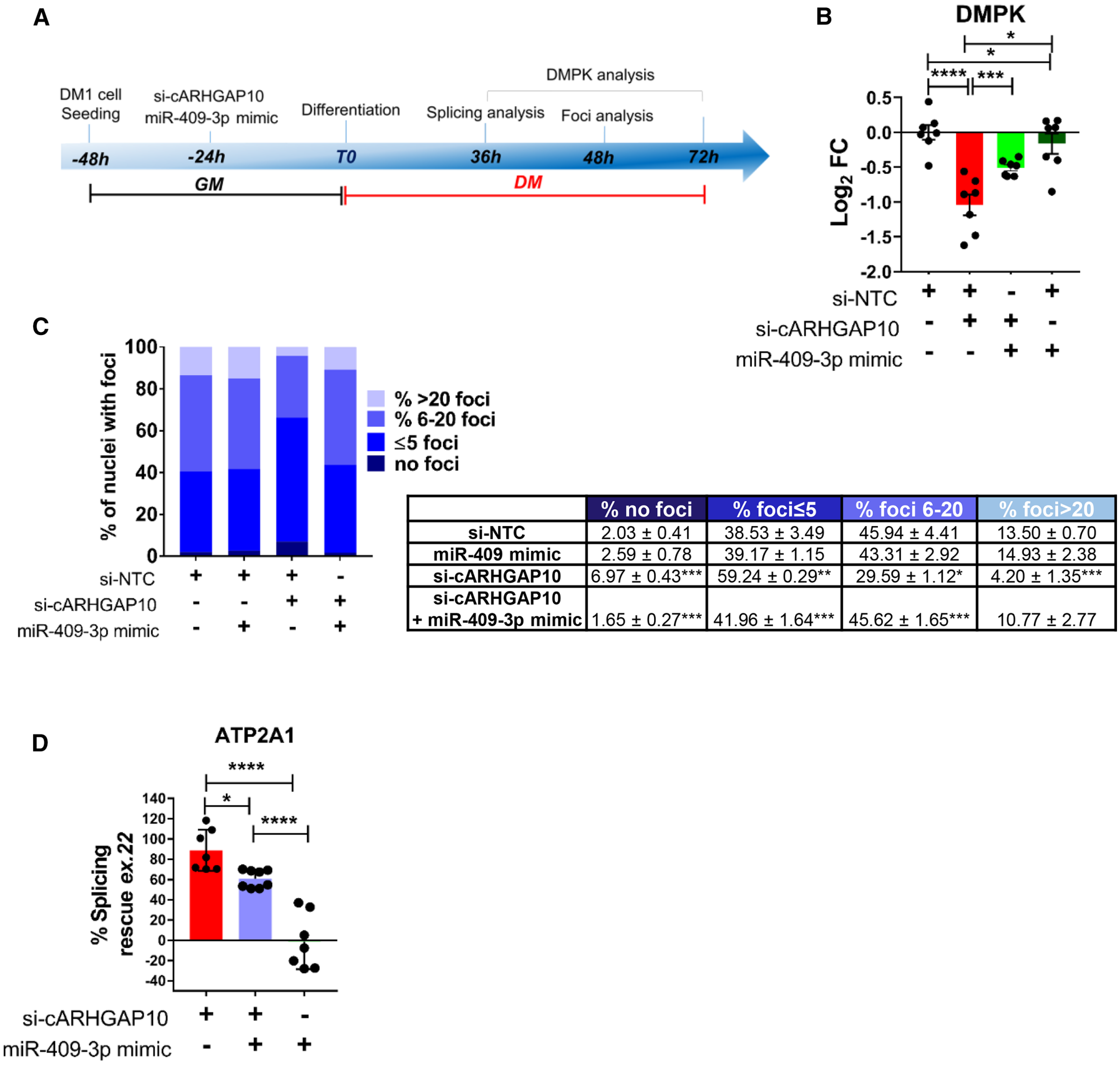

	% no foci	% foci≤5	% foci 6-20	% foci>20
si-NTC	2.03 ± 0.41	38.53 ± 3.49	45.94 ± 4.41	13.50 ± 0.70
miR-409 mimic	2.59 ± 0.78	39.17 ± 1.15	43.31 ± 2.92	14.93 ± 2.38
si-cARHGAP10	6.97 ± 0.43***	59.24 ± 0.29**	29.59 ± 1.12*	4.20 ± 1.35***
si-cARHGAP10 + miR-409-3p mimic	1.65 ± 0.27***	41.96 ± 1.64***	45.62 ± 1.65***	10.77 ± 2.77

Figure 8. miR-409-3p overexpression reduces the effects of circARHGAP10 silencing

(A) Schematic representation of the experimental design used to assess the interaction between miR-409-3p and circARHGAP10 in DM1 myogenic cells. Cells were transfected with siRNAs targeting circARHGAP10 (si-cARHGAP10), an miR-409-3p mimic, or both in combination, and cultured in differentiation medium (DM) for 36 or 48 h. (B) Relative expression of DMPK measured by qPCR in DM1 cells transfected with si-cARHGAP10 (red), co-transfected with miR-409-3p mimic (green), or miR-409-3p mimic alone. Data are shown on $\log_2$ scale ($n = 7$); one-way ANOVA followed by Tukey's post hoc test. (C) Quantification of the percentage of nuclei displaying no foci or increasing numbers of nuclear foci in transfected DM1 cells ($n = 4$). (D) Percent rescue of ATP2A1 transcript isoform splicing in DM1 cells following transfection with si-cARHGAP10, miR-409-3p mimic, or their combination ($n = 5$); one-way ANOVA with Tukey's post hoc test (*$p < 0.05$, ****$p < 0.0001$).

cortex and blood of DM1 patients, suggesting an autonomous regulatory mechanism underlying circRNA accumulation.[28]

While the reasons behind this global increase were not investigated, it may be hypothesized that interferences and lags in pre-mRNA maturation could extend the life of immature transcripts and favor circRNA biogenesis over that of their linear counterparts.[51,52] In DM1, such alterations in RNA maturation may be caused by the reduced bioavailability of MBNL-family factors and the consequent aberrations in splicing, or may be a secondary effect associated with the chronic pathological state of DM1. However, other conditions that share certain physio-pathological features of DM1, such as sarcopenia and limb-girdle muscular dystrophy, did not display a global circRNA increase. Other dystrophies were not investigated, as suitable RNA-sequencing datasets (i.e., not poly-A+ selected) are not yet publicly available. Nonetheless, we can conclude that alteration of global circRNA levels is a DM1 characteristic and is not shared by all myopathies.

Thus, we devised a highly stringent selection pipeline that took advantage of both re-analysis of publicly available RNA-sequencing datasets,[26,29,33] and validation by an orthogonal technique in

independent muscle samples. This allowed the identification of a strong DM1-circRNA signature across different studies and muscle types. These circRNAs show a significant increase in the circ/lin ratio, indicating that the regulation of their biogenesis is distinct from that of their host genes and that they may have unique roles in the molecular mechanisms underlying DM1.

Due to sample availability constraints, the validation was performed in biopsies derived from *biceps brachii*, which is more mildly affected than distal muscles in DM1 patients.[1,53,54] It is plausible to hypothesize that circRNA alterations identified in proximal muscles should be even more pronounced in distal muscles. Likewise, circRNA abundance changes that did not reach statistical significance upon validation in *biceps brachii* might indeed be significant and relevant in distal muscles.

The analytical strategy prioritized specificity over sensitivity, and the study is not intended to be saturating. Accordingly, although circHIPK3 is a component of the DM1-circRNA signature in this study (as previously reported) other circRNAs previously described as dysregulated in *biceps brachii* samples of DM1 patients, such as circCDYL, circRTN4, and circZNF609, were not identified here.[26] On the other hand, the mouse homologue of human circHIPK3 has been found to be dysregulated also in HSA^{LR} mice, a transgenic mouse model of DM1,[29] further confirming the validity of our DM1-circRNA signature.

Notably, all observed dysregulations of DM1-circRNAs were positive.

Given their resistance to exonucleases,[55] circRNAs hold great potential as disease biomarkers. Identifying potential DM1-circRNA biomarkers, combined with genetic analysis, could enhance assessment of disease severity and therapeutic response. Indeed, mRNA missplicing of individual events has been correlated with skeletal muscle performance,[13,33] but this relationship has not been investigated for dysregulated circRNAs, which may further contribute to patient clinical management.

Among DM1-circRNAs, circARHGAP10 has emerged as the most promising candidate biomarker. Indeed, circARHGAP10 expression was higher in more severely affected patients, correlating with CTG repeat length and muscle strength. Moreover, the circ/lin ratio of ARHGAP10 in DM1 patients has a promising potential in discriminating between affected individuals and controls. While these results are highly encouraging, further studies in larger patient cohorts are needed to assess the potential of circARHGAP10 as a biomarker for disease staging/severity and therapeutic monitoring.

Interestingly, silencing circARHGAP10, but not its linear isoform, markedly reduced *DMPK* gene expression, suggesting a role in regulating this key gene involved in DM1 pathogenesis. In DM1, CUG-expanded mutant mRNA accumulates in distinct nuclear aggregates, or foci.[7] Consistent with our experimental data showing reduced *DMPK* transcript levels, silencing of circARHGAP10 led to a significant decrease in RNA foci number, area, and intensity. This indicates that attenuated expression of circARHGAP10, but not its linear counterpart, is associated with decreased nuclear retention of the pathogenic CUG-expanded *DMPK* mRNA, supporting a reduction in toxic RNA foci burden.

Interestingly, circARHGAP10 silencing decreased *DMPK* RNA and the number of foci not only in differentiated myogenic cells, but also in cells grown in GM, indicating that this regulation is independent of the myogenic differentiation process.

A reduction in wild-type *DMPK* transcripts was also observed, suggesting a non–allele-specific targeting effect on *DMPK* mRNA. However, it is reasonable to assume that a considerable portion of the observed reduction involves the mutant allele, as supported by the significant decrease in the number, area, and intensity of RNA foci—hallmarks of the nuclear accumulation of the expanded CUG-containing *DMPK* mRNA. In line with this, Jauvin et al. demonstrated that targeting *DMPK* transcripts in DM1 cells and in a mouse model of DM1 with antisense oligonucleotides (ASOs) reduced the number of foci, and improved body weight and muscle strength, without any evident toxicity.[56] Accordingly, therapeutic strategies using ASOs unable to specifically discriminate between mutant and wild-type *DMPK* transcripts, have demonstrated therapeutic benefit in early-phase clinical trials.[57,58] Specifically, an ASO-based therapeutic compound, AOC 1001, designed for efficient delivery into muscle cells, has successfully passed phase 1/2 clinical trials, reducing *DMPK* mRNA, improving splicing profiles, and various functional parameters in DM1 patients.[57] These findings support the therapeutic relevance of reducing *DMPK* RNA levels, a strategy that is well tolerated in DM1 patients.[57,58]

Analysis of MBNL1 nuclear distribution revealed a significant reduction in its sequestration within nuclear foci upon circARHGAP10 knockdown, suggesting that this targeting strategy mitigates the aberrant retention of MBNL1 likely driven by expanded CUG-containing *DMPK* transcripts. The resulting release of MBNL1 may restore its functional availability in the nucleoplasm, contributing to the partial rescue of splicing defects observed in our DM1 model.

In addition to reducing MBNL1 sequestration, circARHGAP10 silencing led to an increase in MBNL1 protein levels, indicating a broader regulatory role. The observed reduction in the number, size, and intensity of RNA foci upon circARHGAP10 silencing suggests a dual mechanism: improved localization and increased availability of MBNL1. This points to a multifaceted role for circARHGAP10 in modulating MBNL1 function.

Supporting this hypothesis, we observed a significant increase in the percentage of splicing rescue across a panel of alternative splicing events known to be dysregulated in DM1 and associated with reduced MBNL1 activity. Notably, alternative exons in *ATP2A1* exon 22, *INSR* exon 11, *MBNL1* exon 5, *MBNL2* exon 7, *NFIX*

exon 7, *KIF13A* exon 26, *SOS1* exon 21, *CLASP1* exon 19, and *NUMA1* exon 16 exhibited shifts in splicing patterns toward those observed in wild-type myogenic cells following circARHGAP10 knockdown. These splicing events are directly regulated by MBNL1, and several of them have been linked to clinically relevant phenotypes in DM1, such as reduced dorsiflexion strength, impaired Ca^{2+} homeostasis, and insulin resistance in DM1 patients.[13,33,59,60] Interestingly, we observed reduced inclusion of MBNL1 exon 5, an autoregulatory splicing event whose exclusion reduces nuclear retention of the protein.[61–63] In DM1 muscle, this exon is known to be aberrantly included, contributing to the mislocalization and impaired function of MBNL1. While INSR is a known target of both MBNL1 and CUGBP1/CELF1, the observed splicing rescue effects align more closely with MBNL1-dependent splicing patterns and are therefore likely associated with restored MBNL1 availability. The potential contribution of CUGBP1/CELF1 to this regulatory mechanism remains less well defined, requiring further studies. Overall, these findings provide insights into the potential role of circARHGAP10 in DM1 pathogenesis.

The molecular mechanisms of circRNA action are varied and include protein translation, protein binding, and miRNA sponging, the most extensively documented mechanism.[24,25] Here we found that circARHGAP10 interacts with miR-409-3p and the expression levels of both RNAs are upregulated in DM1 patient biopsies compared with controls, suggesting a role in DM1 pathophysiology. Interestingly, miR-409-3p expression has been shown to be associated with mitochondrial damage in muscular dystrophies, although DM1 was not included in that analysis.[64] Regarding skeletal muscle homeostasis and degenerative disorders, a study by Mousa et al. reported a correlation between miR-409-3p upregulation and the severity of muscular weakness in a family affected by Duchenne muscular dystrophy.[65]

circRNAs may act on miRNAs by blocking their canonical targeting, affecting their stability, or a combination of both. Our results suggest that circARHGAP10 might stabilize miR-409-3p, since we found that the two RNAs interacted directly, and their levels correlated *in vitro* and in DM1 patients. In this respect, circARHGAP10 seems to act as a sink for miR-409-3p, contributing to its biological function. Indeed, miR-409-3p overexpression can prevent the amelioration of DM1 hallmarks induced by circARHGAP10 silencing, indicating that miR-409-3p is a relevant effector of circARHGAP10 function. This scenario is similar to the one involving Cdr1as (cerebellar degeneration-related protein 1 antisense RNA), which regulates miR-7 stability, as miR-7 is downregulated in Cdr1as knockout mice,[66] although Cdr1as possesses numerous binding sites for miR-7.[67] Additional studies report a similar protective phenomenon,[68] suggesting a complex regulatory relationship between circRNAs and miRNAs. This underscores the need for further studies investigating the impact of circRNAs on miRNA turnover.

Although miR-409-3p has not been specifically investigated in the context of DM1, and the *DMPK* transcript has not been experimentally confirmed as a direct target of this miRNA, its predicted regulatory network suggests significant involvement in pathways critical to DM1 pathogenesis. Analysis of experimentally supported miRNA-gene interactions indicates that several key transcription factors and chromatin modifiers or RNA-binding proteins are miR-409-3p targets, providing a potential mechanistic explanation for how the interplay between circARHGAP10 and miR-409-3p could impact key features of DM1.

Among them, *CTCF*, a direct target of miR-409-3p described as the "master weaver of the genome," plays a pivotal role in gene silencing and regulation of splicing.[69] Altered methylation at CTCF-binding sites flanking the CTG repeat expansion at the *DMPK* locus has been reported,[70–72] forming a methylation-sensitive insulator that influences genomic architecture and transcriptional regulation at the DM1 locus.[73] *SPEN,* another transcriptional co-repressor targeted by miR-409-3p, has been shown to enhance the neurodegenerative phenotype resulting from the expression of expanded CUG repeat RNAs, thus mediating CUG toxicity in DM1.[74,75] Other targets of miR-409-3p, such as *ZMYND11*,[76] *KDM2A*,[77,78] and *PHF19*,[79–81] are involved in chromatin remodeling and histone modification, which may impact the epigenetic regulation of the *DMPK* gene or alter the expression of neighboring genes, contributing to the complexity of DM1 symptoms. *KDM2A*, for instance, functions as a histone demethylase that regulates transcription by demethylating H3K36me2, thereby influencing chromatin structure and gene expression.[78] *ZMYND11*, known for its role in neurodevelopmental disorders, acts as a transcriptional co-repressor by interacting with histone modifications to regulate RNA polymerase II activity.[78] Additionally, RNA-binding proteins like *QKI*[82–87] and *hnRNP k*[88] are crucial for regulating alternative splicing. Silencing of *hnRNP H*, an *hnRNP* family member,[89] effectively restored the nuclear retention of RNA containing CUG repeat expansions suggesting that hnRNPs play a critical role in binding and potentially modulating the nuclear retention of mutant *DMPK* mRNA.[90] Overexpression of *hnRNP A1*, similar to *CUGBP1*, antagonizes *MBNL1* activity and triggers DM1 disease muscle pathology by promoting fetal splicing patterns.[91] *QKI,*[82–87] an RNA-binding protein, regulates pre-mRNA splicing, circRNA formation, mRNA export, mRNA stability, and/or translation. Given that DM1 is characterized by widespread splicing defects, miR-409-3p modulation of *QKI* could directly influence these pathogenic splicing events.

While there is no direct evidence linking *TNRC6A/C* to *DMPK* regulation, their role in mRNA degradation and miRNA-mediated silencing might influence the post-transcriptional environment affected by DM1.[92] In addition, TNRC6 proteins interact with nuclear RNAs and the nuclear-retained *DMPK* transcripts in DM1, potentially impacting nuclear RNA processing and stability.

Finally, among miR-409-3p targets are the nuclear receptors *RORA*[93] and *RORB,*[94] implicated in circadian rhythm regulation, that may contribute to the sleep-wake circadian disturbances frequently observed in DM1 patients.[95]

Overall, the regulation of these factors by miR-409-3p provides a strong basis for discussing the complex regulatory networks influenced by miR-409-3p. Rather than the overly simplistic "one miRNA - one target - one function" model, data suggest a complex and multi-faceted circARHGAP10-miR-409-3p network, affecting both transcriptional and post-transcriptional processes central to DM1 pathogenesis. Understanding these interactions, although beyond the scope of this investigation, may provide valuable insights into the molecular mechanisms underlying DM1, potentially identifying potential therapeutic targets for managing the disease.

In summary, our findings contribute to the understanding of the implications of circRNAs in DM1. We have defined a DM1-circRNA signature and identified circARHGAP10 as a promising biomarker highlighting its potential as a target aimed at restoring MBNL1 function and correcting downstream splicing abnormalities.

MATERIALS AND METHODS

Patient recruitment and sample collection

Clinical diagnosis of DM1 patients was based on the criteria set by the International Consortium for Myotonic Dystrophies guidelines.[96] DM1 diagnosis was confirmed by genetic analysis. The Muscular Impairment Rating Scale (MIRS) was used to determine the disease stage,[34] whereas the Medical Research Council Muscle Strength (MRC) scale was used to evaluate muscle strength. *Biceps brachii* muscle biopsies collected from 24 DM1 patients and 16 age and sex-matched subjects without signs of neuromuscular disorders (controls), were used for validation (Table S2). The experimental protocol was reviewed and approved by the Institutional Ethics Committee of the San Raffaele Hospital (protocol number: circDM of 11 Nov 2020, Ethics Committee of San Raffaele Hospital) and the study was conducted according to the principles expressed in the Declaration of Helsinki, institutional regulations, and Italian laws and guidelines. Written informed consent was obtained from each patient before muscle biopsy harvesting.

circRNA analysis in publicly available transcriptomic datasets

For the identification of circRNAs deregulated in DM1, we took advantage of previous analyses performed by our group in Voellenkle et al.[26] and by Czubak and colleagues.[29] Both studies re-analyzed published DM1 RNA-seq datasets derived from human *tibialis* muscles (http://dmseq.org/, GSE86356)[33] and both were assisted in their analysis by CIRI2 (version 2.0.6, Computational Genomics Lab, Beijing Institutes of Life Science, Chinese Academy of Sciences, China)[97]; however, they studied different samples and adopted different analysis criteria. Candidate circRNAs displayed a circ/lin ratio >1 in DM1 samples and were significantly ($p < 0.02$) modulated, up, at least 4-fold, in *tibialis anterior* muscle libraries analyzed in Voellenkle et al.[26] They were also significantly (false discovery rate [FDR] <0.1) increased in *tibialis anterior* muscle libraries analyzed in Czubak et al.[29]

Global circRNAs assessment

Quantification of global circRNA levels was performed in datasets GSE86356,[33] GSE201255,[30] GSE111016,[31] GSE111010,[31] and GSE202745,[32] using CIRIquant[98] with default settings, on the GRCh38 genome assembly and the GENCODE v31 basic annotation. Raw count matrices of circRNAs and genes (derived by StringTie, used internally by CIRIquant) were imported into R and converted to normalized values using edgeR's cpm () function, after normalizing the library sizes by the sum of reads mapped to the transcriptome. Comparisons of the cumulative counts-per-million (CPM) values of circRNAs and linear transcripts between diseased and control samples were performed separately for each cohort using two-sided Welch's t test for unequal variances with a significance level of $p < 0.05$. Testing was performed including "all" detectable circRNAs, or including only those that were "commonly" expressed across all samples or in all samples but one.[29] Due to limited library depth, for the GSE202745 dataset the analysis of "common" circRNAs was performed by retaining those expressed in all samples but five.

Target prediction analysis

Experimentally supported targets of hsa-miR-409-3p ($n = 193$) were retrieved from DIANA-TarBase v9.0[46] selecting only those supported by "direct" experimental methods. Their gene symbols were used as input to the online version of Enrichr[99] (https://maayanlab.cloud/Enrichr) and a standard over-representation analysis was performed, denoting terms with FDR <0.05 considered significantly enriched. GO Biological Process results were obtained and the ggplot2 R package was employed to create horizontal bar plots of the top 20 significant terms. Additionally, the manually curated database of Transcriptional Regulatory Relationships TRRUST v2[100] was queried to identify known transcription factors that regulate the transcription of the *DMPK* gene and findings were overlapped with the target list derived from DIANA-TarBase.

Myogenic cell line culture

DM1-myogenic cells, obtained by transducing *TERT*-immortalized human DM1 fibroblasts with lentiviral vectors expressing tetracycline-inducible murine *MyoD1* cDNA, were kindly provided by Dr. D. Furling.[101] Myogenic cells were maintained in growth medium (GM), consisting of DMEM (Gibco, Thermo Fisher Scientific, Waltham, MA, USA) supplemented with 15% heat-inactivated FBS (Sigma-Aldrich, St. Louis, MO, USA) and 0.3 μg/mL puromycin (Serva Electrophoresis, Heidelberg, Germany). Differentiation to myotubes was achieved by growing cells to confluency and replacing the proliferation medium with differentiation medium (DM) consisting of DMEM supplemented with 10 μg/mL insulin, 10 μg/mL transferrin, and 2 μg/mL of doxycycline (Sigma-Aldrich, St. Louis, MO, USA). For RNAi experiments and miRNA overexpression, cells were transfected using Lipofectamine RNAiMAX transfection reagent (Thermo Fisher Scientific, Waltham, MA, USA), following the manufacturer's instructions. siRNAs and miRNA mimics were transfected at a final concentration of 50 nM. siRNAs targeting the circARHGAP10 back-splice junction (si-cARHGAP10) or the linear ARHGAP10 isoform (si-linARHGAP10) were generated by Eurofins Genomics (Ebersberg, Germany) and the oligonucleotide sequences are shown in Table S3. The ON-TARGETplus non-targeting pool

siRNA (Dharmacon, Lafayette, LA, USA) was used as a negative control (si-NTC). miR-409-3p mimic and the corresponding negative control (miR-NC) were purchased from Thermo Fisher Scientific (Waltham, MA, USA). After 6–24 h of incubation, the transfection medium was replaced with DM for 36–72 h, depending on the experimental setting.

Cell proliferation

DM1-myogenic cells (3×10^3) were seeded into 96-well plates and transfected with siRNAs targeting the circular or the linear isoform. Crystal violet colorimetric assay was used to determine cell proliferation at 24, 48, and 72 h post-transfection. After washing with PBS, cells were stained with 50 μL of crystal violet staining solution (Sigma-Aldrich) for 20 minutes at room temperature under gentle shaking. Following three washes in distilled water, plates were left to dry for 2 h at room temperature. Crystal violet was then dissolved in 100 μL of elution buffer (50% ethanol and 0.1% acetic acid) and absorbance was measured at 595 nm, using a Varioskan LUX microplate reader (Thermo Fisher Scientific, Waltham, MA, USA).

Cell apoptosis

DM1-myogenic cells (1.5×10^5) were seeded in six-well plates and transfected with siRNAs targeting the circular or linear isoform. After 48 h of silencing, cells were detached with 1 mM EDTA and resuspended in Annexin V Binding Buffer. Cells were stained with 1 μg/mL propidium iodide (Sigma-Aldrich, Milan, Italy) and Annexin V APC (1:100, ImmunoTools, Friesoythe, Germany). Apoptosis was evaluated by flow cytometry using a BD Biosciences LSR Fortessa X-20 flow cytometer (BD Biosciences, Milan, Italy), followed by data analysis with FlowJo software, version 10 (BD Biosciences, Milan, Italy).

RNA isolation and RNase R digestion

Total RNA was extracted from cells using TRIzol reagent (Thermo Fisher Scientific Inc., Waltham, MA, USA) followed by the RNA Clean & Concentrator-5 kit (Zymo Research, Irvine, CA, USA) according to the manufacturer's instructions. The quantity and purity of the RNA were assessed using a NanoDrop One spectrophotometer (Thermo Fisher Scientific Inc., Waltham, MA, USA). RNA integrity was assessed using an Agilent 2100 Bioanalyzer (Agilent Technologies, Santa Clara, CA, USA). RIN values = 8.0 ± 0.2.

RNase R digestion was performed by incubating 1 μg of total RNA with 1 U of RNase R (Epicentre Biotechnologies, Madison, WI, USA) at 37°C for 10 min, while control samples were incubated with solvent alone. The digestion was then stopped by heating at 95°C for 3 min.

Primer design and qPCR

RNA was reverse transcribed to cDNA using random hexamers and the GoScript Reverse Transcription System (Promega Corporation, Madison, WI, USA). qPCR was performed using an SYBR Green qPCR mix (GoTaq qPCR Master Mix, Promega Corporation, Madison, WI, USA) on a StepOne Plus instrument (Thermo Fisher Scientific, Waltham, MA, USA).

Primer pairs were designed using Primer-BLAST (Table S3). The primers for circRNAs spanned the back-splice junction, while the primers for the linear transcripts crossed the linear junction to a neighboring exon. Primer efficiency analysis was performed for all DM1-associated circRNAs and their corresponding linear counterparts, with efficiencies ranging from 93% to 100%. Primer specificity was assessed through melting curve analysis and confirmation of the expected amplicon size by agarose gel electrophoresis. The absence of signal in no-reverse transcription (no-RT) controls was verified to exclude artifacts. Amplicons spanning the back-splice junctions (BSJs) of circular RNAs were confirmed by Sanger sequencing (Figure S3).

Relative expression was calculated as $\log_2$ fold change, normalized to the averaged cycle threshold (Ct) values of UBC and RPL23. The circ/lin ratios were calculated by subtracting the raw Ct of the linear transcript from the raw Ct of the corresponding circular transcript, as previously described.[26]

Differentially spliced exons in sarcoplasmic/endoplasmic reticulum calcium ATPase 1 (*ATP2A1*) and insulin receptor (*INSR*) were evaluated as previously described.[101,102] Additional alternative splicing events were analyzed by semi-quantitative RT-PCR. Specific validated primers from previous studies were used to amplify the following exons in human DM1 myogenic cells: *MBNL1* exon 5, *MBNL1* exon 6, *NFIX* exon 7, *KIF13A* exon 26, *SOS1* exon 21, *CLASP1* exon 19, and *NUMA1* exon 16. PCR products were separated on 2% agarose gels and quantified by densitometry using Image Studio Lite software (LI-COR Biotechnology). Primer sequences and alternatively spliced exons are annotated according to the current human reference genome (GRCh38/hg38). The original publication from which the primers were derived is referenced (Table S3).

To determine the percent rescue of alternative splicing events, the following Eq. 1 was used, as previously described[103]:

$$\%Rescue = \frac{(\%NTC - \%\ si\_circARHGAP10)}{(\%NTC - \%NTC\ CTRL)} \times 100 \quad \text{(Equation 1)}$$

Pull-down assays

miR-409-3p pull-down assays were performed in DM1 myogenic cells (1×10^6) that were transiently transfected with a mixture containing 50 nM of 3′-biotin-labeled miR-409 mimic (bio-miR-409-3p) and 3′-biotin-labeled negative control (miR-NC) (both from Eurofins Genomics, Ebersberg, Germany), as previously described.[104] DM1 cells were collected 48 h after transfection and lysed with 700 μL of ice-cold lysis buffer (20 mM Tris-HCl, pH 7.5, 100 mM KCl, 5 mM $MgCl_2$, and 0.5% Nonidet P-40) supplemented with 40 U of RNase inhibitor (Promega Corporation, WI, USA) and 5 μL of 20x protease inhibitor (Thermo Fisher Scientific Inc., MA,

USA). The cell lysates were then incubated for 4 h with gentle rotation with 50 μL of pre-coated Dynabeads Streptavidin M-280 (Invitrogen, Waltham, MA, USA) at 4°C. To minimize nonspecific RNA binding, the beads were pretreated with yeast tRNA (Thermo Fisher Scientific Inc., MA, USA) at 4°C for 3 h before incubation with the cell lysates. After washing, RNA was isolated using TRIzol reagent (Thermo Fisher Scientific Inc., MA, USA).

circARHGAP10 pull-down was performed using a 3′-biotin-labeled oligonucleotide complementary to the back-splice junction sequence (bio-circARHGAP10) and a 3′-biotin-labeled control oligonucleotide (bio-NC) (both from Eurofins Genomics, Germany), as previously described.[105] Briefly, DM1 myogenic cells (2×10^6) were lysed in 1 mL of ice-cold polysome extraction buffer (20 mM Tris-HCl, pH 7.5, 100 mM KCl, 5 mM $MgCl_2$, and 0.5% Nonidet P-40) plus protease inhibitors and 40 U RNase inhibitor (Promega Corporation, WI, USA) and centrifuged at 12,000 × *g* for 10 min at 4°C. The supernatants were then incubated on a tube rotator with 1 μL of 100 μM bio-circARHGAP10 or bio-NC overnight at 4°C. After incubation, 50 μL of Dynabeads Streptavidin M-280 (Thermo Fisher Scientific Inc., MA, USA) were added to pull down the complexes, and the mixtures were incubated for 90 min at room temperature. Next, beads were washed four times and RNA was extracted using TRIzol reagent (Thermo Fisher Scientific Inc., MA, USA).

RISC-immunoprecipitation assay

The Magna RIP RNA-Binding Protein Immunoprecipitation Kit (Millipore, , Burlington, MA, USA) was used for RNA immunoprecipitation (RIP) analysis according to the manufacturer's instructions. DM1 cell lysates were incubated with magnetic beads conjugated with either anti-Argonaute2 (AGO2) antibody (Millipore, Billerica, MA, USA) or a negative control IgG antibody (Millipore, Billerica, MA, USA) for 4 h at 4°C with gentle rotation. Following washing, the immunoprecipitated RNA was isolated using TRIzol reagent (Thermo Fisher Scientific Inc., MA, USA).

Western blotting

DM1 myogenic cells were lysed in RIPA buffer supplemented with protease and phosphatase inhibitor cocktails (Roche Diagnostics GmbH). Protein concentrations were detected using a Pierce BCA Protein Assay Kit (Thermo Fisher Scientific). Equal amounts of protein (30 μg per sample) were heated to 95°C for 5 min in SDS loading buffer, separated on 10% SDS polyacrylamide gels and transferred to nitrocellulose membranes (Trans-Blot, Bio-Rad Laboratories, Hercules, CA, USA). Membranes were blocked for 1 h at room temperature in Tris-buffered saline with 0.1% Tween 20 (TBS-T) containing either 5% (w/v) non-fat dry milk or 5% (w/v) bovine serum albumin (BSA; Merck). Membranes were incubated overnight at 4°C with a mouse monoclonal anti-MBNL1 antibody (1:1,000; Sigma-Aldrich). Total protein normalization was performed using the REVERT Total Protein Stain Kit (LI-COR Biotechnology, Lincoln, NE, USA) according to the manufacturer's protocol. After washing, membranes were incubated for 1 h at room temperature with a peroxidase-conjugated anti-rabbit IgG secondary antibody (1:1,000; GE Healthcare Life Sciences). Band intensities were quantified using Image Studio Lite software (LI-COR Biotechnology).

RNA FISH and immunofluorescence staining

DM1 cells were plated on glass slides coated with 50 μg/mL collagen I (Gibco, Thermo Fisher Scientific, Waltham, MA, USA). After transfection, cells grown in GM or differentiated in DM for 48 h were fixed with 2% formaldehyde and subjected to FISH using a $(CAG)_6CA$ probe labeled with Texas Red at the 5′ end (IDT, Coralville, IA, USA), as previously described.[102,106] For combined FISH and immunofluorescence, cells were treated with the Endogenous Biotin-Blocking Kit (Molecular Probes Inc., Eugene, OR, USA), followed by incubation in blocking buffer (3% normal goat serum in PBS). Immunostaining was performed using a mouse monoclonal anti-MBNL1 antibody (clone 3A4, Santa Cruz Biotechnology Inc., TX, USA), a biotin-conjugated goat anti-mouse IgG1 secondary antibody (Jackson ImmunoResearch, PA, USA), and Alexa Fluor 488-conjugated streptavidin (Jackson ImmunoResearch, PA, USA). Nuclei were counterstained with Hoechst 33258. Fluorescence imaging was conducted using an Olympus AX70 microscope. Images were acquired in a blinded manner with an Olympus XM10 camera and processed using CellSens Standard software (v1.8.1; Olympus). Nuclear foci were quantified in a blinded fashion using ImageJ software, with a minimum of 300 nuclei analyzed per condition.

For analysis of foci size and brightness, local thresholding (function thresh() from package EBImage[107]) was applied on the blue channel to generate a binary mask of nuclei (parameters w = 250, h = 250, offset = 0.06), and on the red channel to generate a binary mask of foci (parameters w = 4, h = 4, offset = 0.02). Morphological operations were applied to refine the segmentation and filter out nuclei touching the image borders. A watershed algorithm was used to identify and label individual nuclei and foci, and segmentation success was inspected visually. Foci were associated with nuclei by determining the spatial overlap between their segmentations. Measurements of mean intensity per nuclear focus (arbitrary units ranging from 0 to 1) and percentage of nuclear area occupied by foci were recorded. The extracted data exhibited a nested structure, i.e., foci within nuclei, within samples (individual images), within experiments. To account for this while comparing si-circARHGAP10, si-linARHGAP10, and si-NTC, mixed-effects models were employed using lmerTest package.[108] The model formula for contrasts of nuclear area percentage difference was "area ∼ state + (1|experiment) + (1|experiment:sample)," while for intensity, the variability of nuclei was included (i.e., "variable ∼ state + (1|experiment) + (1|experiment:sample) + (1|experiment:sample:nucleus))."

For analysis of co-staining images, local thresholding was applied to generate a binary mask of nuclei (parameters w = 100, h = 100, and offset = 0.05), foci (parameters w = 4, h = 4, and offset = 0.02), and MBNL1 (the union of two threshold operations with parameters $w_1 = 4$, $h_1 = 4$, $offset_1 = 0.05$ and $w_2 = 2$, $h_2 = 2$, $offset_2 = 0.1$), again using watershed algorithm for identification of individual nuclei, foci, and MBNL1 areas. Spatial overlaps between nuclei, foci, and

MBNL1 areas were used to define MBNL1-containing nuclear foci and record their measurements. To account for the nested data structure while comparing si-circARHGAP10 against si-NTC, a mixed-effects linear model (lmerTest, formula: "area ~ state + (1| batch) + (1|batch:sample)") and a mixed-effects negative binomial model (glmmTMB package,[109] formula: "foci ~ state + (1| batch) + (1| batch:sample)") were employed for continuous and count data respectively.

The function emmeans() (by emmeans package[110]) was called to perform pairwise comparison of the three treatments, applying *p*-value adjustment using the Tukey's method, where applicable. Estimated marginal means (with SEM), were used to present differences between treatments. A minimum of 600 nuclei per condition were analyzed.

Statistical analysis

Statistical analyses were performed using GraphPad Prism version 7.01 (GraphPad Software, San Diego, CA, USA). Data are presented as mean ± standard error of the mean (SEM). The distribution of each dataset was assessed using the Shapiro-Wilk test. For comparisons between two groups, an unpaired two-tailed Student's t test was applied when data were normally distributed with equal variances, while Welch's t test was used in cases of unequal variances. Non-normally distributed data or data with unequal variances were analyzed using the Mann-Whitney *U* test. For comparisons involving more than two groups, one-way analysis of variance (ANOVA) followed by Tukey's or Dunnett's post hoc test was used for parametric data. Pearson's correlation coefficient was used to assess linear relationships between two continuous variables. *p* values used in this study to determine statistical significance are indicated as follows: *$p < 0.05$, **$p < 0.01$, ***$p < 0.001$, ****$p < 0.0001$.

DATA AND CODE AVAILABILITY

The authors declare that all data supporting the findings of this study are available within the paper and its supplemental information files. Raw imaging data are available upon reasonable request.

ACKNOWLEDGMENTS

F.M. is partially supported by Ricerca Corrente funding from Italian Ministry of Health to IRCCS Policlinico San Donato (#1.07.128; #1.07.125; #1.07.127; #1.07.129). F.M. is also supported by the Italian Ministry of Health (POS-T4 CAL.HUB.RIA T4-AN-09), by the European Union (Next Generation EU-NRRP M6C2 Inv. 2.1 PNRR-MAD 2022-12375790 and PNRR-MCNT2-2023-12377983, and Romania's PNRR-III-C9-2022-I8, CF 186/24.11.2022, contr. 760062/23.05.2023) and by Fondazione Malattie Miotoniche ETS-Fondo Monica Stupino. F.M. and G.F. were funded by AFM-Téléthon (no. 23054) and Telethon-Italy (no. GGP19035). D.B. was supported by the Italian Ministry of Health (GR-019-12370076). A.P. received funding from the Italian Ministry of Health (SG-2019-12368989). M.I. and D.B. were recipients of fellowships funded by Telethon-Italy.

We thank Dr. Denis Furling for providing DM1 myogenic cell lines and Sarah Placida (IRCCS Policlinico San Donato, Milan, Italy) for her support in clinical data collection.

AUTHOR CONTRIBUTIONS

F.M. and G.F. conceived and supervised the study and secured funding. D.B., A.P., C.P., S.T., M.I., M.L., S.F., and B.C. performed experiments, analyzed data, and prepared figures. A.S.T., S.T., and C.V. performed bioinformatics analyses. R.C. and G.M. recruited patients and collected muscle biopsies. D.B. and F.M. wrote the manuscript. All authors reviewed and approved the final version of the manuscript.

DECLARATION OF INTERESTS

The authors declare no competing interests.

DECLARATION OF GENERATIVE AI AND AI-ASSISTED TECHNOLOGIES IN THE WRITING PROCESS

During the preparation of this work the authors used ChatGPT (OpenAI, San Francisco, CA) in order to improve the grammar and clarity of the manuscript. After using this tool/service, the authors reviewed and edited the content as needed and take full responsibility for the content of the publication.

SUPPLEMENTAL INFORMATION

Supplemental information can be found online at https://doi.org/10.1016/j.omtn.2025.102646.

REFERENCES

1. Meola, G., and Cardani, R. (2015). Myotonic dystrophies: An update on clinical aspects, genetic, pathology, and molecular pathomechanisms. Biochim. Biophys. Acta *1852*, 594–606.
2. Harper, P.S., van Engelen, B.G.M., Eymard, B., Rogers, M., and Wilcox, D. (2002). 99th ENMC international workshop: myotonic dystrophy: present management, future therapy. 9-11 November 2001, Naarden, The Netherlands. Neuromuscul. Disord. *12*, 596–599.
3. Brook, J.D., McCurrach, M.E., Harley, H.G., Buckler, A.J., Church, D., Aburatani, H., Hunter, K., Stanton, V.P., Thirion, J.P., and Hudson, T. (1992). Molecular basis of myotonic dystrophy: expansion of a trinucleotide (CTG) repeat at the 3' end of a transcript encoding a protein kinase family member. Cell *69*, 385.
4. Mahadevan, M., Tsilfidis, C., Sabourin, L., Shutler, G., Amemiya, C., Jansen, G., Neville, C., Narang, M., Barceló, J., and O'Hoy, K. (1992). Myotonic dystrophy mutation: an unstable CTG repeat in the 3' untranslated region of the gene. Science *255*, 1253–1255.
5. Overend, G., Légaré, C., Mathieu, J., Bouchard, L., Gagnon, C., and Monckton, D. G. (2019). Allele length of the DMPK CTG repeat is a predictor of progressive myotonic dystrophy type 1 phenotypes. Hum. Mol. Genet. *28*, 2245–2254.
6. Konieczny, P., Stepniak-Konieczna, E., and Sobczak, K. (2014). MBNL proteins and their target RNAs, interaction and splicing regulation. Nucleic Acids Res. *42*, 10873–10887.
7. Pettersson, O.J., Aagaard, L., Jensen, T.G., and Damgaard, C.K. (2015). Molecular mechanisms in DM1 - a focus on foci. Nucleic Acids Res. *43*, 2433–2441.
8. Kalsotra, A., Xiao, X., Ward, A.J., Castle, J.C., Johnson, J.M., Burge, C.B., and Cooper, T.A. (2008). A postnatal switch of CELF and MBNL proteins reprograms alternative splicing in the developing heart. Proc. Natl. Acad. Sci. USA *105*, 20333–20338.
9. Lee, K.Y., Li, M., Manchanda, M., Batra, R., Charizanis, K., Mohan, A., Warren, S. A., Chamberlain, C.M., Finn, D., Hong, H., et al. (2013). Compound loss of muscleblind-like function in myotonic dystrophy. EMBO Mol. Med. *5*, 1887–1900.
10. Wang, E.T., Cody, N.A.L., Jog, S., Biancolella, M., Wang, T.T., Treacy, D.J., Luo, S., Schroth, G.P., Housman, D.E., Reddy, S., et al. (2012). Transcriptome-wide regulation of pre-mRNA splicing and mRNA localization by muscleblind proteins. Cell *150*, 710–724.
11. Imbriano, C., and Molinari, S. (2018). Alternative Splicing of Transcription Factors Genes in Muscle Physiology and Pathology. Genes *9*, 107.
12. Lopez-Martinez, A., Soblechero-Martin, P., de-la-Puente-Ovejero, L., Nogales-Gadea, G., and Arechavala-Gomeza, V. (2020). An Overview of Alternative Splicing Defects Implicated in Myotonic Dystrophy Type I. Genes *11*, 1109.
13. Nakamori, M., Sobczak, K., Puwanant, A., Welle, S., Eichinger, K., Pandya, S., Dekdebrun, J., Heatwole, C.R., McDermott, M.P., Chen, T., et al. (2013). Splicing biomarkers of disease severity in myotonic dystrophy. Ann. Neurol. *74*, 862–872.

14. Thomas, J.D., Oliveira, R., Sznajder, Ł.J., and Swanson, M.S. (2018). Myotonic Dystrophy and Developmental Regulation of RNA Processing. Compr. Physiol. *8*, 509–553.

15. Izzo, M., Battistini, J., Provenzano, C., Martelli, F., Cardinali, B., and Falcone, G. (2022). Molecular Therapies for Myotonic Dystrophy Type 1: From Small Drugs to Gene Editing. Int. J. Mol. Sci. *23*, 4622.

16. Perbellini, R., Greco, S., Sarra-Ferraris, G., Cardani, R., Capogrossi, M.C., Meola, G., and Martelli, F. (2011). Dysregulation and cellular mislocalization of specific miRNAs in myotonic dystrophy type 1. Neuromuscul. Disord. *21*, 81–88.

17. Garcia, S.M.D.A., Tabach, Y., Lourenço, G.F., Armakola, M., and Ruvkun, G. (2014). Identification of genes in toxicity pathways of trinucleotide-repeat RNA in C. elegans. Nat. Struct. Mol. Biol. *21*, 712–720.

18. Kristensen, L.S., Andersen, M.S., Stagsted, L.V.W., Ebbesen, K.K., Hansen, T.B., and Kjems, J. (2019). The biogenesis, biology and characterization of circular RNAs. Nat. Rev. Genet. *20*, 675–691.

19. Patop, I.L., Wüst, S., and Kadener, S. (2019). Past, present, and future of circRNAs. EMBO J. *38*, e100836.

20. Ebbesen, K.K., Hansen, T.B., and Kjems, J. (2017). Insights into circular RNA biology. RNA Biol. *14*, 1035–1045.

21. Carrara, M., Fuschi, P., Ivan, C., and Martelli, F. (2018). Circular RNAs: Methodological challenges and perspectives in cardiovascular diseases. J. Cell Mol. Med. *22*, 5176–5187.

22. Greco, S., Cardinali, B., Falcone, G., and Martelli, F. (2018). Circular RNAs in Muscle Function and Disease. Int. J. Mol. Sci. *19*, 3454.

23. Li, X., Yang, L., and Chen, L.L. (2018). The Biogenesis, Functions, and Challenges of Circular RNAs. Mol. Cell *71*, 428–442.

24. Han, B., Chao, J., and Yao, H. (2018). Circular RNA and its mechanisms in disease: From the bench to the clinic. Pharmacol. Ther. *187*, 31–44.

25. Hansen, T.B., Jensen, T.I., Clausen, B.H., Bramsen, J.B., Finsen, B., Damgaard, C.K., and Kjems, J. (2013). Natural RNA circles function as efficient microRNA sponges. Nature *495*, 384–388.

26. Voellenkle, C., Perfetti, A., Carrara, M., Fuschi, P., Renna, L.V., Longo, M., Sain, S. B., Cardani, R., Valaperta, R., Silvestri, G., et al. (2019). Dysregulation of Circular RNAs in Myotonic Dystrophy Type 1. Int. J. Mol. Sci. *20*, 1938.

27. Czubak, K., Sedehizadeh, S., Kozlowski, P., and Wojciechowska, M. (2019). An overview of circular RNAs and their implications in myotonic dystrophy. Int. J. Mol. Sci. *20*, 4385.

28. Srinivasan, A., Magner, D., Kozłowski, P., Philips, A., Kajdasz, A., Wojciechowski, P., and Wojciechowska, M. (2025). Global dysregulation of circular RNAs in frontal cortex and whole blood from DM1 and DM2. Hum. Genet. *144*, 417–432.

29. Czubak, K., Taylor, K., Piasecka, A., Sobczak, K., Kozlowska, K., Philips, A., Sedehizadeh, S., Brook, J.D., Wojciechowska, M., and Kozlowski, P. (2019). Global Increase in Circular RNA Levels in Myotonic Dystrophy. Front. Genet. *10*, 649.

30. Hale, M.A., Bates, K., Provenzano, M., and Johnson, N.E. (2023). Dynamics and variability of transcriptomic dysregulation in congenital myotonic dystrophy during pediatric development. Hum. Mol. Genet. *32*, 1413–1428.

31. Migliavacca, E., Tay, S.K.H., Patel, H.P., Sonntag, T., Civiletto, G., McFarlane, C., Forrester, T., Barton, S.J., Leow, M.K., Antoun, E., et al. (2019). Mitochondrial oxidative capacity and NAD(+) biosynthesis are reduced in human sarcopenia across ethnicities. Nat. Commun. *10*, 5808.

32. Depuydt, C.E., Goosens, V., Janky, R., D'Hondt, A., De Bleecker, J.L., Noppe, N., Derveaux, S., Thal, D.R., and Claeys, K.G. (2022). Unraveling the Molecular Basis of the Dystrophic Process in Limb-Girdle Muscular Dystrophy LGMD-R12 by Differential Gene Expression Profiles in Diseased and Healthy Muscles. Cells *11*, 1508.

33. Wang, E.T., Treacy, D., Eichinger, K., Struck, A., Estabrook, J., Olafson, H., Wang, T.T., Bhatt, K., Westbrook, T., Sedehizadeh, S., et al. (2019). Transcriptome alterations in myotonic dystrophy skeletal muscle and heart. Hum. Mol. Genet. *28*, 1312–1321.

34. Mathieu, J., Boivin, H., Meunier, D., Gaudreault, M., and Bégin, P. (2001). Assessment of a disease-specific muscular impairment rating scale in myotonic dystrophy. Neurology *56*, 336–340.

35. Bibi, A., Madè, A., Greco, S., Garcia-Manteiga, J.M., Tascini, A.S., Tastsoglou, S., Zaccagnini, G., Leszek, P., Gaetano, C., and Martelli, F. (2025). Circular PVT1 promotes cardiac fibroblast activation interacting with miR-30a-5p and miR-125b-5p. Cell Death Dis. *16*, 325.

36. Zheng, Q., Bao, C., Guo, W., Li, S., Chen, J., Chen, B., Luo, Y., Lyu, D., Li, Y., Shi, G., et al. (2016). Circular RNA profiling reveals an abundant circHIPK3 that regulates cell growth by sponging multiple miRNAs. Nat. Commun. *7*, 11215.

37. Miyagawa, R., Tano, K., Mizuno, R., Nakamura, Y., Ijiri, K., Rakwal, R., Shibato, J., Masuo, Y., Mayeda, A., Hirose, T., and Akimitsu, N. (2012). Identification of cis- and trans-acting factors involved in the localization of MALAT-1 noncoding RNA to nuclear speckles. RNA *18*, 738–751.

38. Carrell, S.T., Carrell, E.M., Auerbach, D., Pandey, S.K., Bennett, C.F., Dirksen, R.T., and Thornton, C.A. (2016). Dmpk gene deletion or antisense knockdown does not compromise cardiac or skeletal muscle function in mice. Hum. Mol. Genet. *25*, 4328–4338.

39. Thornton, C.A., Arnold, W.D., Ashizawa, T., Day, J.W., Dent, G., Tanner, M.K., Moxley, R.T., 3rd, Eichinger, K., Heatwole, C., Mignon, L., et al. (2023). Antisense oligonucleotide targeting DMPK in patients with myotonic dystrophy type 1: a multicentre, randomised, dose-escalation, placebo-controlled, phase 1/2a trial. Lancet Neurol. *22*, 218–228.

40. Davis, B.M., McCurrach, M.E., Taneja, K.L., Singer, R.H., and Housman, D.E. (1997). Expansion of a CUG trinucleotide repeat in the 3' untranslated region of myotonic dystrophy protein kinase transcripts results in nuclear retention of transcripts. Proc. Natl. Acad. Sci. USA *94*, 7388–7393.

41. Charizanis, K., Lee, K.Y., Batra, R., Goodwin, M., Zhang, C., Yuan, Y., Shiue, L., Cline, M., Scotti, M.M., Xia, G., et al. (2012). Muscleblind-like 2-mediated alternative splicing in the developing brain and dysregulation in myotonic dystrophy. Neuron *75*, 437–450.

42. Wagner, S.D., Struck, A.J., Gupta, R., Farnsworth, D.R., Mahady, A.E., Eichinger, K., Thornton, C.A., Wang, E.T., and Berglund, J.A. (2016). Dose-dependent regulation of alternative splicing by MBNL proteins reveals biomarkers for myotonic dystrophy. PLoS Genet. *12*, e1006316.

43. Sebestyén, E., Singh, B., Miñana, B., Pagès, A., Mateo, F., Pujana, M.A., Valcárcel, J., and Eyras, E. (2018). Corrigendum: Large-scale analysis of genome and transcriptome alterations in multiple tumors unveils novel cancer-relevant splicing networks. Genome Res. *28*, 1426.

44. Ellis, J.A., Hale, M.A., Cleary, J.D., Wang, E.T., and Andrew Berglund, J. (2023). Alternative splicing outcomes across an RNA-binding protein concentration gradient. J. Mol. Biol. *435*, 168156.

45. Ray, D., Yun, Y.C., Idris, M., Cheng, S., Boot, A., Iain, T.B.H., Rozen, S.G., Tan, P., and Epstein, D.M. (2020). A tumor-associated splice-isoform of MAP2K7 drives dedifferentiation in MBNL1-low cancers via JNK activation. Proc. Natl. Acad. Sci. USA *117*, 16391–16400.

46. Skoufos, G., Kakoulidis, P., Tastsoglou, S., Zacharopoulou, E., Kotsira, V., Miliotis, M., Mavromati, G., Grigoriadis, D., Zioga, M., Velli, A., et al. (2024). TarBase-v9.0 extends experimentally supported miRNA-gene interactions to cell-types and virally encoded miRNAs. Nucleic Acids Res. *52*, D304–D310.

47. Dudekula, D.B., Panda, A.C., Grammatikakis, I., De, S., Abdelmohsen, K., and Gorospe, M. (2016). CircInteractome: A web tool for exploring circular RNAs and their interacting proteins and microRNAs. RNA Biol. *13*, 34–42.

48. Meister, G., Landthaler, M., Patkaniowska, A., Dorsett, Y., Teng, G., and Tuschl, T. (2004). Human Argonaute2 mediates RNA cleavage targeted by miRNAs and siRNAs. Mol. Cell *15*, 185–197.

49. Bibi, A., Bartekova, M., Gandhi, S., Greco, S., Made, A., Sarkar, M., Stopa, V., Tastsoglou, S., de Gonzalo-Calvo, D., Devaux, Y., et al. (2024). Circular RNA regulatory role in pathological cardiac remodelling. Br. J. Pharmacol. *182*, 316–339.

50. Made, A., Bibi, A., Garcia-Manteiga, J.M., Tascini, A.S., Piella, S.N., Tikhomirov, R., Voellenkle, C., Gaetano, C., Leszek, P., Castelvecchio, S., et al. (2023). circRNA-miRNA-mRNA Deregulated Network in Ischemic Heart Failure Patients. Cells *12*, 2578.

51. Ashwal-Fluss, R., Meyer, M., Pamudurti, N.R., Ivanov, A., Bartok, O., Hanan, M., Evantal, N., Memczak, S., Rajewsky, N., and Kadener, S. (2014). circRNA biogenesis competes with pre-mRNA splicing. Mol. Cell *56*, 55–66.

52. Liang, D., Tatomer, D.C., Luo, Z., Wu, H., Yang, L., Chen, L.L., Cherry, S., and Wilusz, J.E. (2017). The Output of Protein-Coding Genes Shifts to Circular RNAs When the Pre-mRNA Processing Machinery Is Limiting. Mol. Cell *68*, 940–954.e3.

53. De Antonio, M., Dogan, C., Hamroun, D., Mati, M., Zerrouki, S., Eymard, B., Katsahian, S., and Bassez, G.; French Myotonic Dystrophy Clinical Network (2016). Unravelling the myotonic dystrophy type 1 clinical spectrum: A systematic registry-based study with implications for disease classification. Rev. Neurol. *172*, 572–580.

54. Gagnon, C., Chouinard, M.C., Laberge, L., Veillette, S., Bégin, P., Breton, R., Jean, S., Brisson, D., Gaudet, D., and Mathieu, J.; DMI Expert Panel (2010). Health supervision and anticipatory guidance in adult myotonic dystrophy type 1. Neuromuscul. Disord. *20*, 847–851.

55. Ebbesen, K.K., Kjems, J., and Hansen, T.B. (2016). Circular RNAs: Identification, biogenesis and function. Biochim. Biophys. Acta *1859*, 163–168.

56. Jauvin, D., Chrétien, J., Pandey, S.K., Martineau, L., Revillod, L., Bassez, G., Lachon, A., MacLeod, A.R., Gourdon, G., Wheeler, T.M., et al. (2017). Targeting DMPK with Antisense Oligonucleotide Improves Muscle Strength in Myotonic Dystrophy Type 1 Mice. Mol. Ther. Nucleic Acids *7*, 465–474.

57. Johnson, N., Day, J., Hamel, J., Thornton, C., Subramony, S., Soltanzadeh, P., Statland, J., Wicklund, M., Arnold, W., Freimer, M., et al. (2023). VP55 Topline data analysis of the phase 1/2 clinical trial evaluating AOC 1001 in adult patients with myotonic dystrophy type 1: MARINA. Neuromuscul. Disord. *33*, S72.

58. Wolf, D., Bassez, G., Diaz-Manera, J., Kools, J., Lilleker, J., Pane, M., Roxburgh, R., Schoser, B., Turner, C., Mix, C., et al. (2025). Safety and Efficacy of DYNE-101 in Adults with DM1: Phase 1/2 ACHIEVE Trial Data (S16. 003). Neurology *104*, 2675.

59. Nieuwenhuis, S., Okkersen, K., Widomska, J., Blom, P., t Hoen, P.A.C., van Engelen, B., and Glennon, J.C. (2019). Insulin Signaling as a Key Moderator in Myotonic Dystrophy Type 1. Front. Neurol. *10*, 1229.

60. Zhao, Y., Ogawa, H., Yonekura, S.I., Mitsuhashi, H., Mitsuhashi, S., Nishino, I., Toyoshima, C., and Ishiura, S. (2015). Functional analysis of SERCA1b, a highly expressed SERCA1 variant in myotonic dystrophy type 1 muscle. Biochim. Biophys. Acta *1852*, 2042–2047.

61. Tran, H., Gourrier, N., Lemercier-Neuillet, C., Dhaenens, C.-M., Vautrin, A., Fernandez-Gomez, F.J., Arandel, L., Carpentier, C., Obriot, H., Eddarkaoui, S., et al. (2011). Analysis of exonic regions involved in nuclear localization, splicing activity, and dimerization of Muscleblind-like-1 isoforms. J. Biol. Chem. *286*, 16435–16446.

62. Kanadia, R.N., Shin, J., Yuan, Y., Beattie, S.G., Wheeler, T.M., Thornton, C.A., and Swanson, M.S. (2006). Reversal of RNA missplicing and myotonia after muscleblind overexpression in a mouse poly (CUG) model for myotonic dystrophy. Proc. Natl. Acad. Sci. USA *103*, 11748–11753.

63. Gates, D.P., Coonrod, L.A., and Berglund, J.A. (2011). Autoregulated splicing of muscleblind-like 1 (MBNL1) Pre-mRNA. J. Biol. Chem. *286*, 34224–34233.

64. Aksu-Menges, E., Akkaya-Ulum, Y.Z., Dayangac-Erden, D., Balci-Peynircioglu, B., Yuzbasioglu, A., Topaloglu, H., Talim, B., and Balci-Hayta, B. (2020). The Common miRNA Signatures Associated with Mitochondrial Dysfunction in Different Muscular Dystrophies. Am. J. Pathol. *190*, 2136–2145.

65. Mousa, N.O., Sayed, A.A., Fahmy, N., Elzayat, M.G., Bakry, U., Abdellatif, A., Zahra, W.K., and Osman, A. (2021). miRNome profiling in Duchenne muscular dystrophy; identification of asymptomatic and manifesting female carriers. Biosci. Rep. *41*, BSR20211325.

66. Piwecka, M., Glazar, P., Hernandez-Miranda, L.R., Memczak, S., Wolf, S.A., Rybak-Wolf, A., Filipchyk, A., Klironomos, F., Cerda Jara, C.A., Fenske, P., et al. (2017). Loss of a mammalian circular RNA locus causes miRNA deregulation and affects brain function. Science *357*, eaam8526.

67. Memczak, S., Jens, M., Elefsinioti, A., Torti, F., Krueger, J., Rybak, A., Maier, L., Mackowiak, S.D., Gregersen, L.H., Munschauer, M., et al. (2013). Circular RNAs are a large class of animal RNAs with regulatory potency. Nature *495*, 333–338.

68. Bezzi, M., Guarnerio, J., and Pandolfi, P.P. (2017). A circular twist on microRNA regulation. Cell Res. *27*, 1401–1402.

69. Alharbi, A.B., Schmitz, U., Bailey, C.G., and Rasko, J.E.J. (2021). CTCF as a regulator of alternative splicing: new tricks for an old player. Nucleic Acids Res. *49*, 7825–7838.

70. Filippova, G.N., Thienes, C.P., Penn, B.H., Cho, D.H., Hu, Y.J., Moore, J.M., Klesert, T.R., Lobanenkov, V.V., and Tapscott, S.J. (2001). CTCF-binding sites flank CTG/CAG repeats and form a methylation-sensitive insulator at the DM1 locus. Nat. Genet. *28*, 335–343.

71. Morales, F., Corrales, E., Zhang, B., Vásquez, M., Santamaría-Ulloa, C., Quesada, H., Sirito, M., Estecio, M.R., Monckton, D.G., and Krahe, R. (2021). Myotonic dystrophy type 1 (DM1) clinical subtypes and CTCF site methylation status flanking the CTG expansion are mutant allele length-dependent. Hum. Mol. Genet. *31*, 262–274.

72. Cho, D.H., Thienes, C.P., Mahoney, S.E., Analau, E., Filippova, G.N., and Tapscott, S.J. (2005). Antisense transcription and heterochromatin at the DM1 CTG repeats are constrained by CTCF. Mol. Cell *20*, 483–489.

73. Lopez Castel, A., Nakamori, M., Tome, S., Chitayat, D., Gourdon, G., Thornton, C. A., and Pearson, C.E. (2011). Expanded CTG repeat demarcates a boundary for abnormal CpG methylation in myotonic dystrophy patient tissues. Hum. Mol. Genet. *20*, 1–15.

74. Dansithong, W., Jog, S.P., Paul, S., Mohammadzadeh, R., Tring, S., Kwok, Y., Fry, R.C., Marjoram, P., Comai, L., and Reddy, S. (2011). RNA steady-state defects in myotonic dystrophy are linked to nuclear exclusion of SHARP. EMBO Rep. *12*, 735–742.

75. Mutsuddi, M., Marshall, C.M., Benzow, K.A., Koob, M.D., and Rebay, I. (2004). The spinocerebellar ataxia 8 noncoding RNA causes neurodegeneration and associates with staufen in Drosophila. Curr. Biol. *14*, 302–308.

76. Wang, J., Qin, S., Li, F., Li, S., Zhang, W., Peng, J., Zhang, Z., Gong, Q., Wu, J., and Shi, Y. (2014). Crystal structure of human BS69 Bromo-ZnF-PWWP reveals its role in H3K36me3 nucleosome binding. Cell Res. *24*, 890–893.

77. Reischl, S., and Kramer, A. (2015). Fbxl11 Is a Novel Negative Element of the Mammalian Circadian Clock. J. Biol. Rhythms *30*, 291–301.

78. Liu, L., Liu, J., and Lin, Q. (2021). Histone demethylase KDM2A: Biological functions and clinical values. Exp. Ther. Med. *22*, 723.

79. Ballare, C., Lange, M., Lapinaite, A., Martin, G.M., Morey, L., Pascual, G., Liefke, R., Simon, B., Shi, Y., Gozani, O., et al. (2012). Phf19 links methylated Lys36 of histone H3 to regulation of Polycomb activity. Nat. Struct. Mol. Biol. *19*, 1257–1265.

80. Brien, G.L., Gambero, G., O'Connell, D.J., Jerman, E., Turner, S.A., Egan, C.M., Dunne, E.J., Jurgens, M.C., Wynne, K., Piao, L., et al. (2012). Polycomb PHF19 binds H3K36me3 and recruits PRC2 and demethylase NO66 to embryonic stem cell genes during differentiation. Nat. Struct. Mol. Biol. *19*, 1273–1281.

81. Chen, S., Jiao, L., Liu, X., Yang, X., and Liu, X. (2020). A Dimeric Structural Scaffold for PRC2-PCL Targeting to CpG Island Chromatin. Mol. Cell *77*, 1265–1278.e7.

82. Zhao, Z., Qing, Y., Dong, L., Han, L., Wu, D., Li, Y., Li, W., Xue, J., Zhou, K., Sun, M., et al. (2023). QKI shuttles internal m(7)G-modified transcripts into stress granules and modulates mRNA metabolism. Cell *186*, 3208–3226.e27.

83. Caines, R., Cochrane, A., Kelaini, S., Vila-Gonzalez, M., Yang, C., Eleftheriadou, M., Moez, A., Stitt, A.W., Zeng, L., Grieve, D.J., and Margariti, A. (2019). The RNA-binding protein QKI controls alternative splicing in vascular cells, producing an effective model for therapy. J. Cell Sci. *132*, jcs230276.

84. de Bruin, R.G., Shiue, L., Prins, J., de Boer, H.C., Singh, A., Fagg, W.S., van Gils, J. M., Duijs, J.M.G.J., Katzman, S., Kraaijeveld, A.O., et al. (2016). Quaking promotes monocyte differentiation into pro-atherogenic macrophages by controlling pre-mRNA splicing and gene expression. Nat. Commun. *7*, 10846.

85. Conn, S.J., Pillman, K.A., Toubia, J., Conn, V.M., Salmanidis, M., Phillips, C.A., Roslan, S., Schreiber, A.W., Gregory, P.A., and Goodall, G.J. (2015). The RNA binding protein quaking regulates formation of circRNAs. Cell *160*, 1125–1134.

86. Teplova, M., Hafner, M., Teplov, D., Essig, K., Tuschl, T., and Patel, D.J. (2013). Structure-function studies of STAR family Quaking proteins bound to their in vivo RNA target sites. Genes Dev. *27*, 928–940.

87. Fu, H., Yang, G., Wei, M., Liu, L., Jin, L., Lu, X., Wang, L., Shen, L., Zhang, J., Lu, H., et al. (2012). The RNA-binding protein QKI5 is a direct target of C/EBPalpha and delays macrophage differentiation. Mol. Biol. Cell *23*, 1628–1635.

88. Hollensen, A.K., Thomsen, H.S., Lloret-Llinares, M., Kamstrup, A.B., Jensen, J.M., Luckmann, M., Birkmose, N., Palmfeldt, J., Jensen, T.H., Hansen, T.B., and Damgaard, C.K. (2020). circZNF827 nucleates a transcription inhibitory complex to balance neuronal differentiation. eLife *9*, e58478.

89. Geuens, T., Bouhy, D., and Timmerman, V. (2016). The hnRNP family: insights into their role in health and disease. Hum. Genet. *135*, 851–867.

90. Kim, D.H., Langlois, M.A., Lee, K.B., Riggs, A.D., Puymirat, J., and Rossi, J.J. (2005). HnRNP H inhibits nuclear export of mRNA containing expanded CUG repeats and a distal branch point sequence. Nucleic Acids Res. *33*, 3866–3874.

91. Li, M., Zhuang, Y., Batra, R., Thomas, J.D., Li, M., Nutter, C.A., Scotti, M.M., Carter, H.A., Wang, Z.J., Huang, X.S., et al. (2020). HNRNPA1-induced spliceopathy in a transgenic mouse model of myotonic dystrophy. Proc. Natl. Acad. Sci. USA *117*, 5472–5477.

92. Chaston, J.J., Stewart, A.G., and Christie, M. (2017). Structural characterisation of TNRC6A nuclear localisation signal in complex with importin-alpha. PLoS One *12*, e0183587.

93. Hwang, E.J., Lee, J.M., Jeong, J., Park, J.H., Yang, Y., Lim, J.S., Kim, J.H., Baek, S.H., and Kim, K.I. (2009). SUMOylation of RORalpha potentiates transcriptional activation function. Biochem. Biophys. Res. Commun. *378*, 513–517.

94. Mansour, H.A., Talkowski, M.E., Wood, J., Chowdari, K.V., McClain, L., Prasad, K., Montrose, D., Fagiolini, A., Friedman, E.S., Allen, M.H., et al. (2009). Association study of 21 circadian genes with bipolar I disorder, schizoaffective disorder, and schizophrenia. Bipolar Disord. *11*, 701–710.

95. Liguori, C., Spanetta, M., Fernandes, M., Placidi, F., Massa, R., Romigi, A., Izzi, F., Mauro, L., Greco, G., Frezza, E., et al. (2021). The actigraphic documentation of circadian sleep-wake rhythm dysregulation in myotonic dystrophy type 1. Sleep Med. *88*, 134–139.

96. Udd, B., Meola, G., Krahe, R., Thornton, C., Ranum, L.P.W., Bassez, G., Kress, W., Schoser, B., and Moxley, R. (2006). 140th ENMC International Workshop: Myotonic Dystrophy DM2/PROMM and other myotonic dystrophies with guidelines on management. Neuromuscul. Disord. *16*, 403–413.

97. Gao, Y., Wang, J., and Zhao, F. (2015). CIRI: an efficient and unbiased algorithm for de novo circular RNA identification. Genome Biol. *16*, 4.

98. Zhang, J., Chen, S., Yang, J., and Zhao, F. (2020). Accurate quantification of circular RNAs identifies extensive circular isoform switching events. Nat. Commun. *11*, 90.

99. Xie, Z., Bailey, A., Kuleshov, M.V., Clarke, D.J.B., Evangelista, J.E., Jenkins, S.L., Lachmann, A., Wojciechowicz, M.L., Kropiwnicki, E., Jagodnik, K.M., et al. (2021). Gene Set Knowledge Discovery with Enrichr. Curr. Protoc. *1*, e90.

100. Han, H., Cho, J.W., Lee, S., Yun, A., Kim, H., Bae, D., Yang, S., Kim, C.Y., Lee, M., Kim, E., et al. (2018). TRRUST v2: an expanded reference database of human and mouse transcriptional regulatory interactions. Nucleic Acids Res. *46*, D380–D386.

101. Arandel, L., Polay Espinoza, M., Matloka, M., Bazinet, A., De Dea Diniz, D., Naouar, N., Rau, F., Jollet, A., Edom-Vovard, F., Mamchaoui, K., et al. (2017). Immortalized human myotonic dystrophy muscle cell lines to assess therapeutic compounds. Dis. Model. Mech. *10*, 487–497.

102. Cardinali, B., Provenzano, C., Izzo, M., Voellenkle, C., Battistini, J., Strimpakos, G., Golini, E., Mandillo, S., Scavizzi, F., Raspa, M., et al. (2022). Time-controlled and muscle-specific CRISPR/Cas9-mediated deletion of CTG-repeat expansion in the DMPK gene. Mol. Ther. Nucleic Acids *27*, 184–199.

103. Jenquin, J.R., O'Brien, A.P., Poukalov, K., Lu, Y., Frias, J.A., Shorrock, H.K., Richardson, J.I., Mazdiyasni, H., Yang, H., Huigens, R.W., 3rd, et al. (2022). Molecular characterization of myotonic dystrophy fibroblast cell lines for use in small molecule screening. iScience *25*, 104198.

104. Dash, S., Balasubramaniam, M., Dash, C., and Pandhare, J. (2018). Biotin-based Pulldown Assay to Validate mRNA Targets of Cellular miRNAs. J. Vis. Exp. *136*, 57786.

105. Das, D., Das, A., and Panda, A.C. (2021). Antisense Oligo Pulldown of Circular RNA for Downstream Analysis. Bio. Protoc. *11*, e4088.

106. Cardani, R., Mancinelli, E., Sansone, V., Rotondo, G., and Meola, G. (2004). Biomolecular identification of (CCTG)n mutation in myotonic dystrophy type 2 (DM2) by FISH on muscle biopsy. Eur. J. Histochem. *48*, 437–442.

107. Pau, G., Fuchs, F., Sklyar, O., Boutros, M., and Huber, W. (2010). EBImage—an R package for image processing with applications to cellular phenotypes. Bioinformatics *26*, 979–981.

108. Kuznetsova, A., Brockhoff, P.B., and Christensen, R.H.B. (2017). lmerTest package: tests in linear mixed effects models. J. Stat. Softw. *82*, 1–26.

109. Brooks, ME, Kristensen, K, Van Benthem, KJ, Magnusson, A, Berg, CW, Nielsen, A, Skaug, HJ, Mächler, M, Bolker, BM.(2017). glmmTMB balances speed and flexibility among packages for zero-inflated generalized linear mixed modeling.

110. Lenth, R., and Lenth, M.R. (2018). Package 'lsmeans'. Am. Statistician *34*, 216–221.

OMTN, Volume *36*

Supplemental information

circARHGAP10 as a candidate biomarker and therapeutic target in myotonic dystrophy type 1

Denisa Baci, Spyros Tastsoglou, Claudia Provenzano, Alessandra Perfetti, Mariapaola Izzo, Mario Lisanti, Svetlana Frolova, Christine Voellenkle, Anna Sofia Tascini, Rosanna Cardani, Beatrice Cardinali, Giovanni Meola, Germana Falcone, and Fabio Martelli

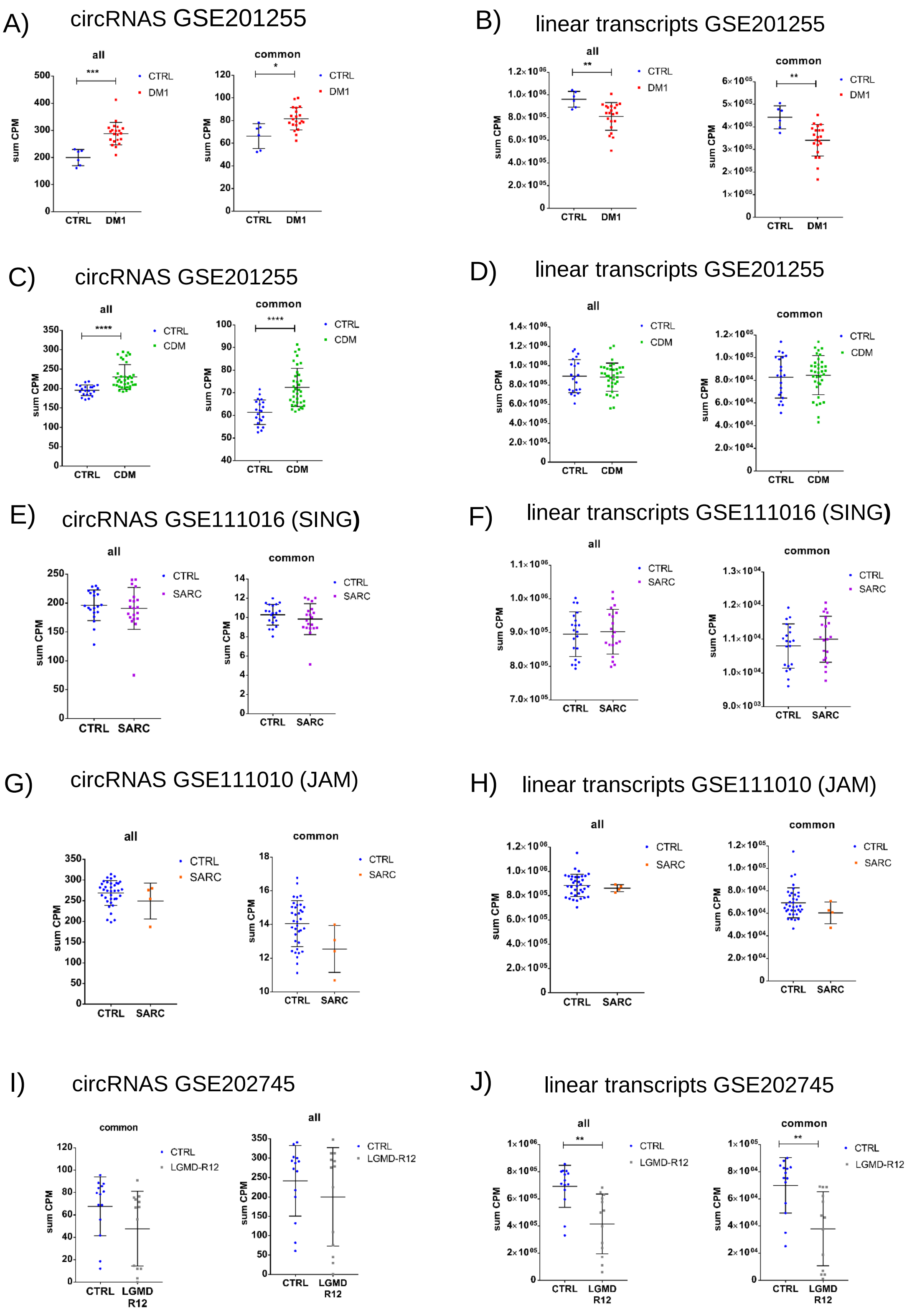
A) circRNAS GSE201255
B) linear transcripts GSE201255
C) circRNAS GSE201255
D) linear transcripts GSE201255
E) circRNAS GSE111016 (SING)
F) linear transcripts GSE111016 (SING)
G) circRNAS GSE111010 (JAM)
H) linear transcripts GSE111010 (JAM)
I) circRNAS GSE202745
J) linear transcripts GSE202745
all
common
sum CPM
CTRL
DM1
CDM
SARC
LGMD-R12
LGMD R12

Figure S1. Assessment of global circRNA levels in muscle tissues across various myopathies.

Barplots showing global circRNA levels (A, C, E, G, I) and their corresponding linear transcripts (B, D, F, H, J). (A-B) Contrast between adult DM1 (n=22) and control (CTRL, n=7) samples from the GSE201255 dataset. (C-D) Contrast between congenital DM1 (CDM, n=36) and pediatric controls (n=21) from the GSE201255 dataset. (E-F) Contrast between sarcopenia (SARC, n=20) and control samples (n=20) from the Singapore cohort ("SING") in the GSE111016 dataset. (G-H) Contrast between sarcopenia (n=9) and control samples (n=30) from the Jamaica cohort ("JAM") in the GSE111010 dataset. (I-J) Contrast between Limb Girdle Muscular Dystrophy R12 (LGMD-R12, n=13) and control samples (n=14) in the GSE202745 dataset. Each point represents the sum of counts-per-million (CPM) reads mapped to the transcriptome, normalized for library depth. Two contrasts are shown for each panel: on the left ("all"), values of all expressed circRNAs (or their linear transcripts) are summed for each sample and compared between disease and control; on the right ("common"), only circRNAs expressed in at least all-but-one sample in each cohort are included. For LGMD-R12 (I-J), a less stringent criterion was used due to library size limitations. DM1 vs. CTRL comparisons were performed using a two-sided Welch's t-test (*$p < 0.05$, **$p < 0.01$, ***$p < 0.001$, ****$p < 0.0001$).

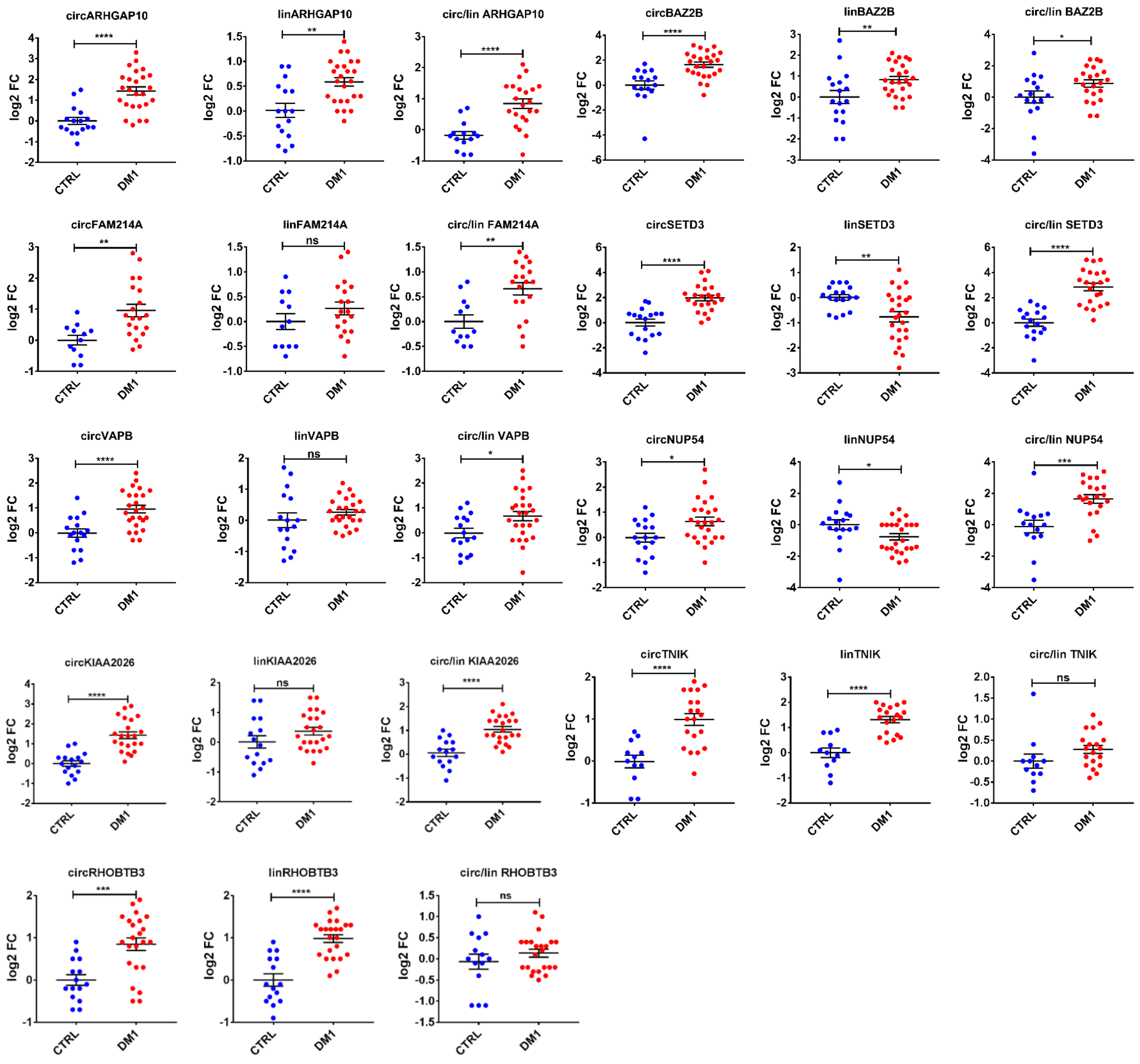

Figure S2. qPCR validation of DM1-circRNA candidates.

qPCR validation of DM1-circRNA expression levels and circ/lin ratios in biceps brachii samples from DM1 and control (CTRL) groups. Barplots show increased expression of nine DM1-circRNAs in DM1 samples compared to controls, along with significantly altered circ/lin ratios, except for RHOBTB3 and TNIK. Data are shown on a $\log_2$ scale. Differences between groups were evaluated using the Mann–Whitney U test. CTRL (blue dots, n = 16); DM1 (red dots, n = 24) (*$p < 0.05$, **$p < 0.01$, ***$p < 0.001$, ****$p < 0.0001$).

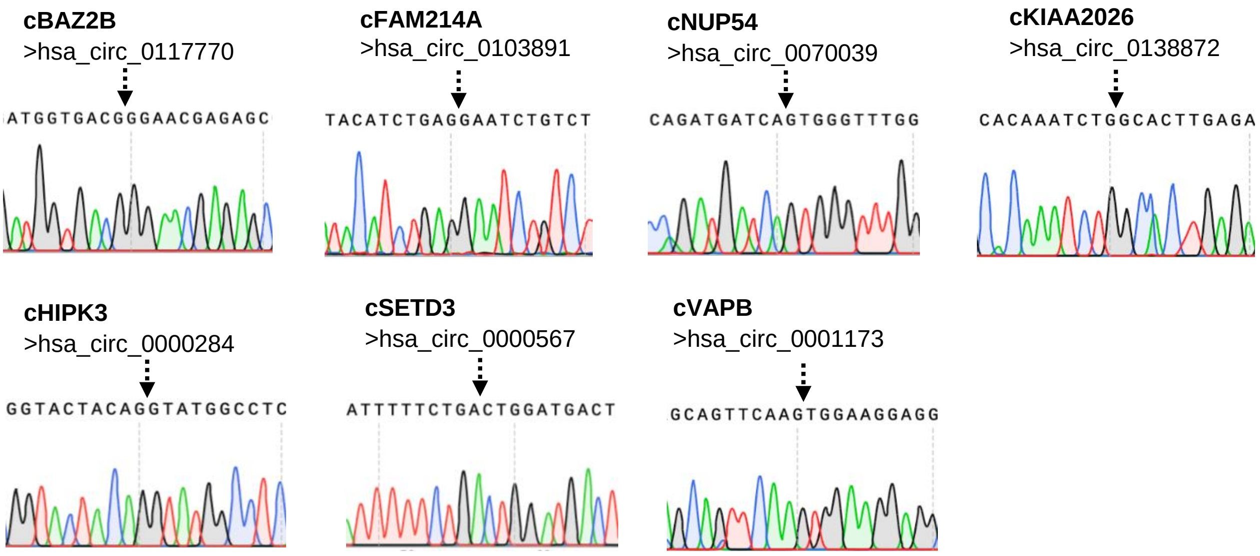

Figure S3. Sanger sequencing of DM1-circRNA candidates expressed in biceps brachii samples from DM1 patients.
For each circRNA, the expected back-splice junction sequence was confirmed. The arrow and chromatograms show the position of the back-splice junction, validating the circular structure of the transcripts.

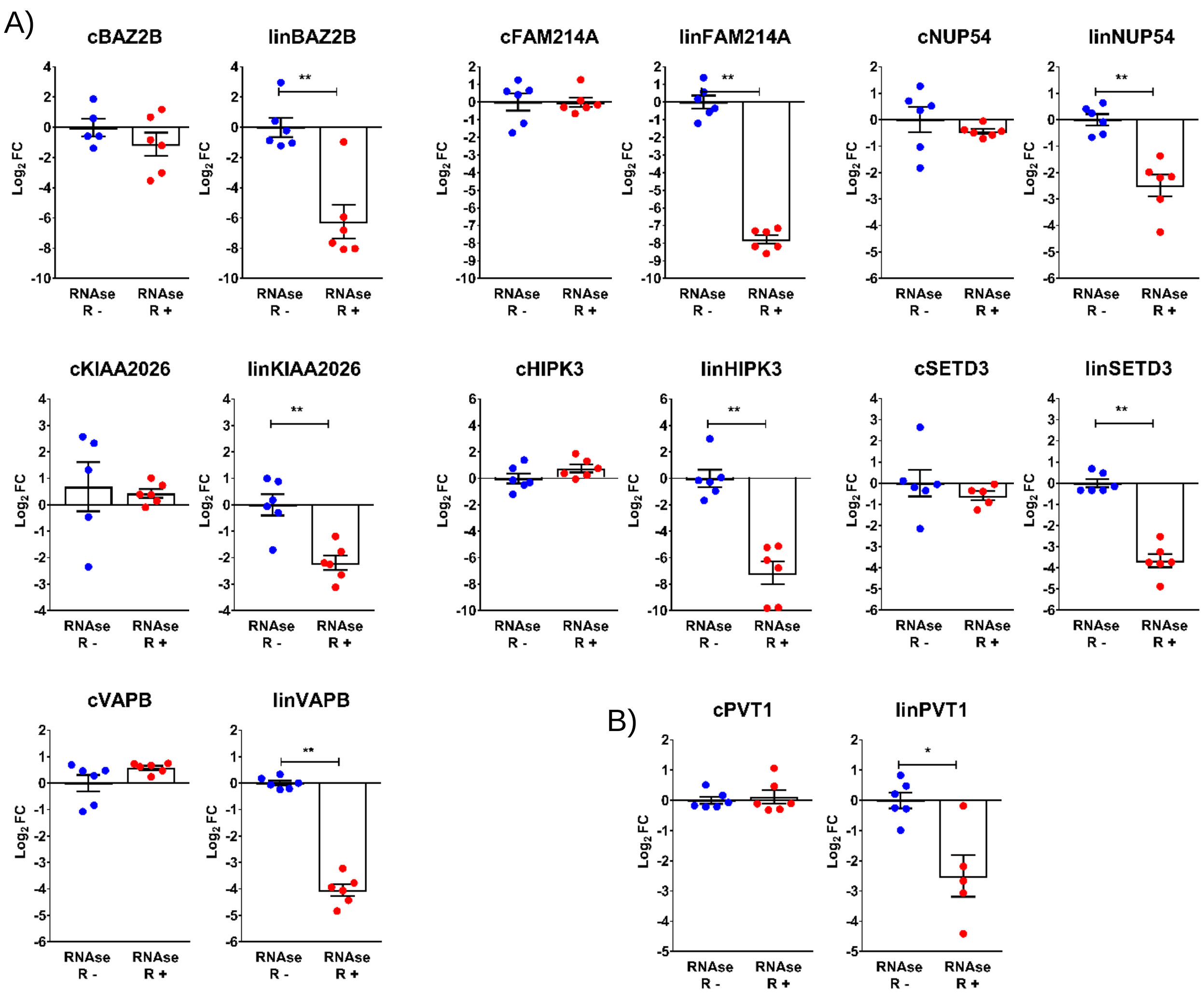

Figure S4. DM1-circRNA candidates are resistant to RNase R digestion. (A) qPCR analysis of selected DM1-associated circRNA candidates and their corresponding linear transcripts was performed on total RNA treated with (+RNase R) or without (−RNase R) RNase R exonuclease. CircRNAs exhibited resistance to RNase R treatment, while the corresponding linear transcripts were significantly degraded. (B) RNase R resistance of circPVT1 (cPVT1) and linear PVT1 (linPVT1) was assessed as a control. As expected, cPVT1 was resistant to RNase R digestion, whereas linPVT1 was significantly degraded, confirming effective exonuclease treatment. Data are presented as mean ± SEM on a $\log_2$ scale ($n = 5–6$). Statistical significance was determined using a two-tailed Mann–Whitney U test (*$p < 0.05$, **$p < 0.01$).

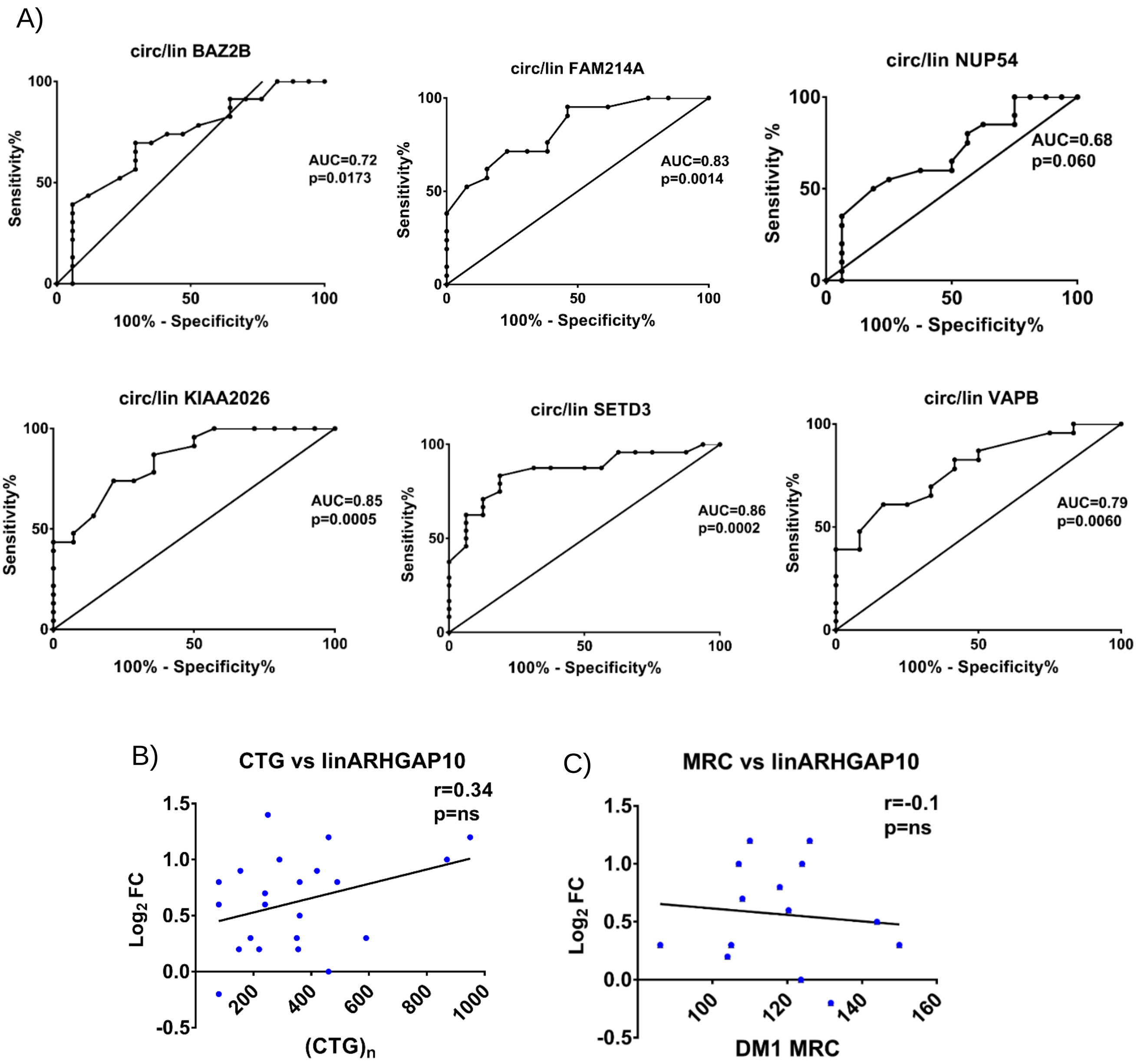

Figure S5. Discrimination of DM1 patients from healthy controls using circ/lin RNA expression ratios of DM1-circRNA candidates.

(A) Receiver operating characteristic (ROC) curve showing the sensitivity and specificity of the circRNA/linear RNA (circ/lin) expression ratios of selected DM1-circRNA candidates in distinguishing DM1 muscle biopsies from healthy controls.(B-C) Pearson correlation analysis between linARHGAP10 expression levels in biceps brachii muscle biopsies and (B) CTG repeat size, and (C) skeletal muscle strength measured by the Medical Research Council (MRC) megascore. Each data point represents an individual DM1 patient sample (n = 24, shown in blue).

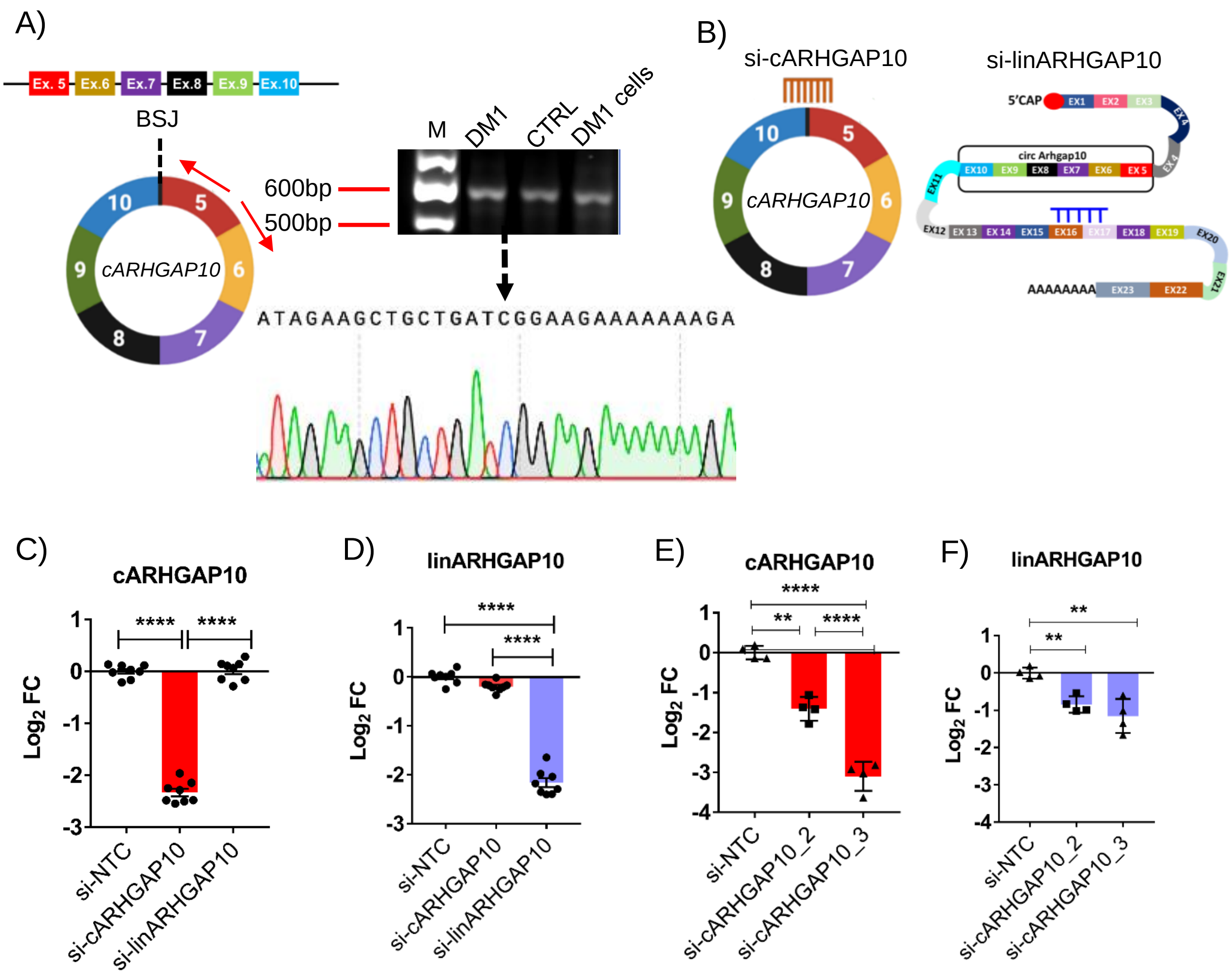

Figure S6. circARHGAP10 sequence validation and specific silencing.
(A) Diagram illustrating circARHGAP10 structure, showing the junction site between exon 10 and exon 5. The complete sequence was confirmed by Sanger sequencing of the amplicon generated using the divergent primers shown in red. (B) Design of siRNAs targeting the back-splice junction (si-cARHGAP10) to knock down circARHGAP10, and siRNAs targeting exons not involved in circularization (si-linARHGAP10) to knock down the linear isoform. (C-D) Barplots showing qPCR–measured expression levels of circARHGAP10 (C) and linARHGAP10 (D) in DM1 myogenic cells transfected with isoform-specific siRNAs or a non-targeting control (si-NTC). (E–F) Expression levels of circARHGAP10 (E) and linARHGAP10 (F) after transfection with additional siRNAs (si-cARHGAP10_2 and si-cARHGAP10_3) targeting the back-splice junction. These siRNAs showed strong knockdown efficiency but reduced isoform specificity compared to those in C–D. Data are presented as mean ± SEM on a $\log_2$ scale ($n = 8$ for C–D; $n = 4$ for E–F;), analyzed by one-way ANOVA with Tukey's post hoc test ($^{**}p < 0.01$, $^{****}p < 0.0001$).

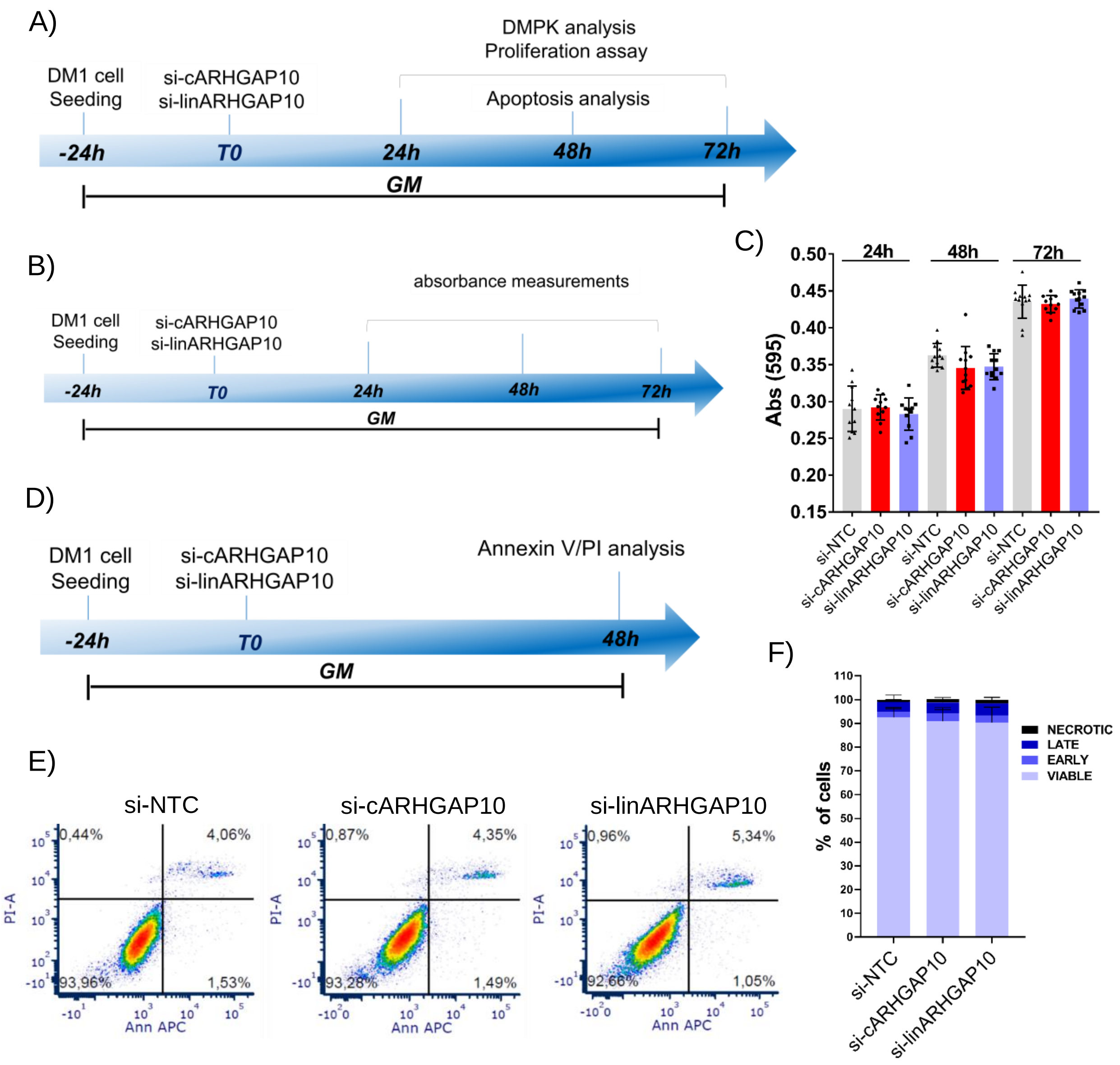

Figure S7. Silencing of circARHGAP10 and linARHGAP10 does not affect DM1 cell proliferation and apoptosis.

(A) Schematic of the siRNA transfection protocol in DM1 myogenic cells cultured in growth medium (GM). (B) Proliferation timeline using crystal violet assay. Cells were transfected with siRNAs targeting circARHGAP10 (si-cARHGAP10), linARHGAP10 (si-linARHGAP10), or a non-targeting control (si-NTC), and cultured in GM. (C) Cell proliferation was assessed at 24, 48, and 72 hours post-transfection by measuring absorbance at 595 nm. No statistically significant differences were observed. Data are presented as mean ± SEM (n = 11), analyzed by one-way ANOVA with Tukey's post hoc test, comparing each group to si-NTC at each time point. (D-E) Apoptosis timeline assessed 48 hours post-transfection using annexin V-APC/PI staining and representative FACS plots. (F) Bar graphs showing the percentages of viable, early apoptotic, late apoptotic, and necrotic cells. No significant differences were observed between groups. Data are presented as mean ± SEM (n = 6), analyzed by two-way ANOVA with Dunnett's post hoc test, comparing each group to si-NTC.

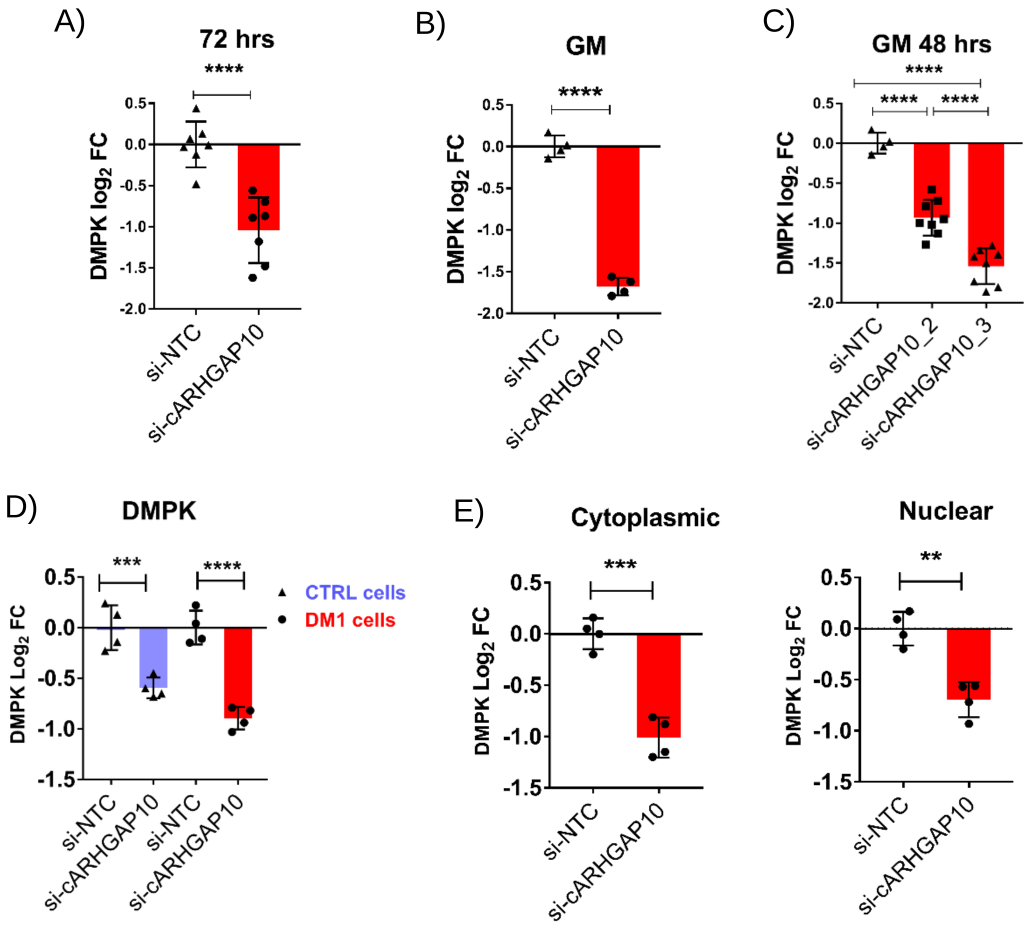

Figure S8. circARHGAP10 silencing decreases DMPK expression.

(A–E) Barplots showing DMPK expression levels measured by qPCR in DM1 myogenic cells transfected with siRNAs targeting circARHGAP10 (si-cARHGAP10) or non-targeting control siRNAs (si-NTC). (A) DMPK levels measured after 72 h in differentiation medium ($n = 7$; two-tailed unpaired t-test). (B) DMPK levels measured in growth medium (GM) ($n = 4$; two-tailed unpaired t-test). (C) DMPK expression in DM1 myogenic cells transfected with two independent siRNAs targeting circARHGAP10 (si-cARHGAP10_2 and si-cARHGAP10_3; $n = 8$; one-way ANOVA with Tukey's post hoc test). (D) DMPK expression in differentiated control (CTRL, blue) and DM1 (red) myogenic cells transfected with si-cARHGAP10 or si-NTC ($n = 4$; one-way ANOVA with Tukey's post hoc test). (E) DMPK expression in cytoplasmic and nuclear fractions of DM1 myogenic cells transfected with si-cARHGAP10 or si-NTC ($n = 4$; two-tailed unpaired t-test) ,($^{**}p < 0.01$, $^{***}p < 0.001$, $^{****}p < 0.0001$).

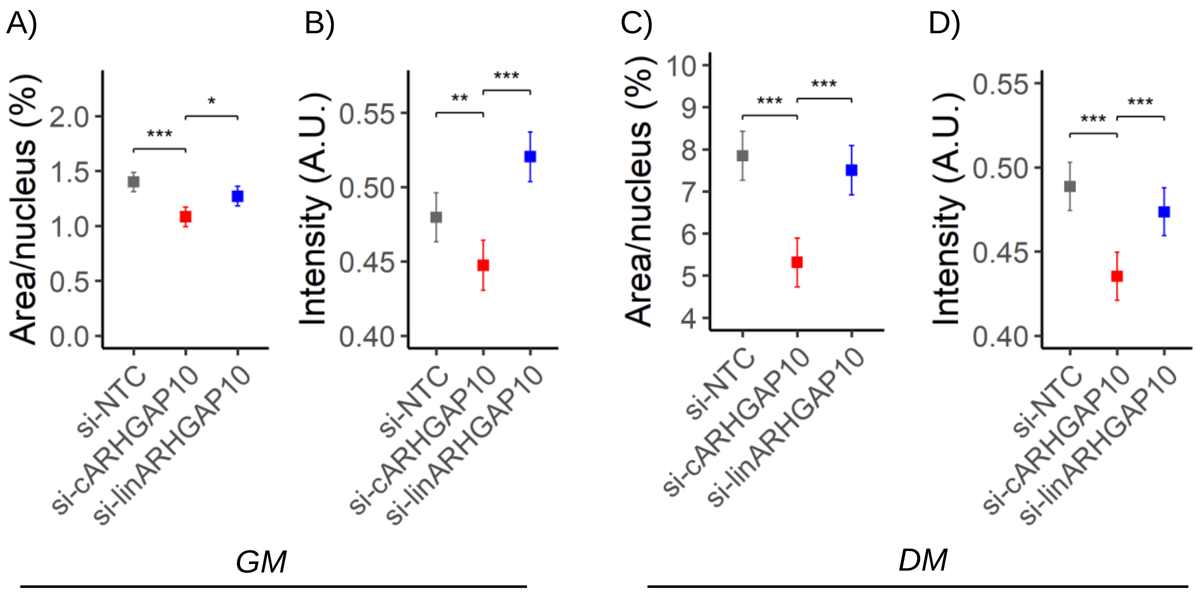

Figure S9. Silencing of circARHGAP10 reduces both the size and intensity of CUG foci. DM1 cells were transfected with siRNAs targeting circARHGAP10 (si-cARHGAP10_1), linARHGAP10 (si-linARHGAP10), or non-targeting control siRNAs (si-NTC), and cultured in either growth medium (GM) or differentiation medium (DM) for 48 h prior to RNA FISH analysis. Linear mixed-effects models were used to compare the percentage of nuclear area occupied by CUG foci (A, C) and the mean fluorescence intensity per focus (arbitrary units, A.U.) (B, D) across treatment conditions. Quantification was performed in DM1 cells cultured in GM (A–B; n = 5) and DM (C–D; n = 4) from independent experiments. Data points represent model-derived estimated marginal means. Error bars indicate SEM. Statistical significance was determined using linear mixed-effects models with Satterthwaite's method for degrees of freedom and Tukey-adjusted post hoc pairwise comparisons (*$p < 0.05$, **$p < 0.01$, ***$p < 0.001$).

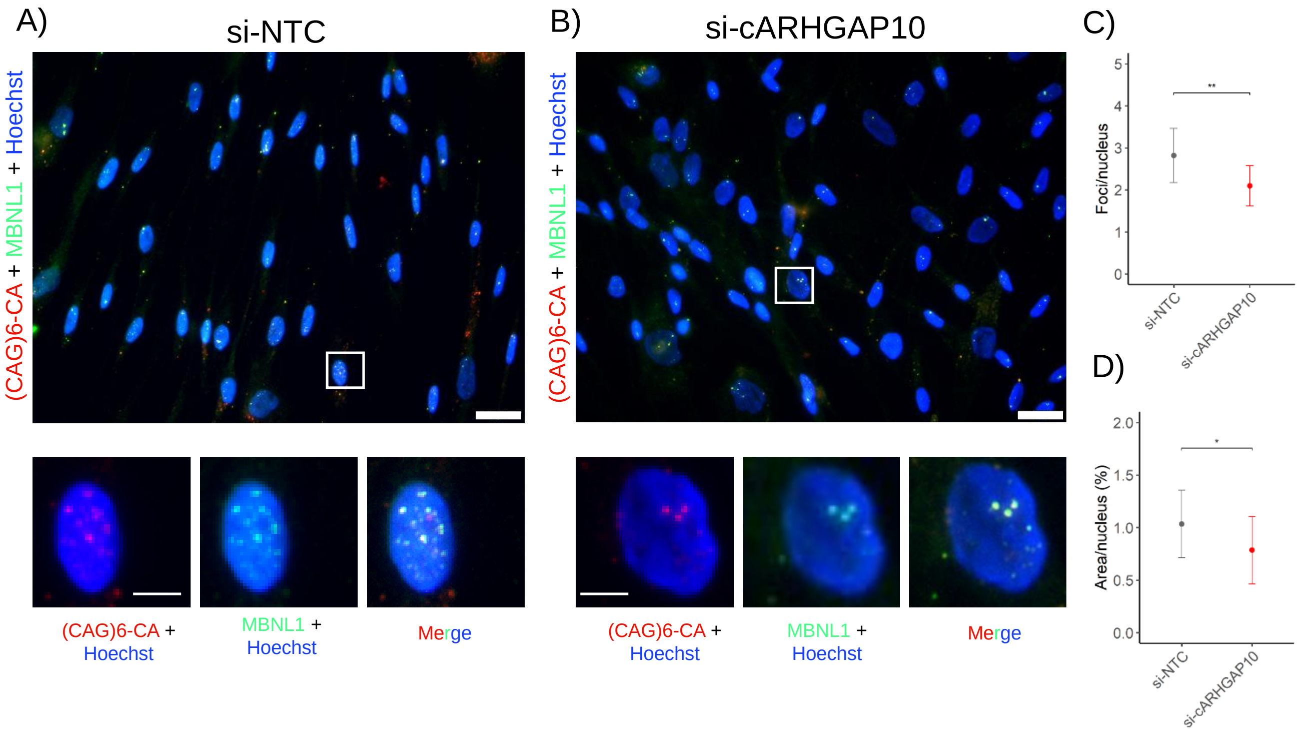

Figure S10. Silencing of circARHGAP10 reduces MBNL1 sequestration in differentiated DM1 myogenic cells.

(A–B) Representative images of RNA FISH combined with MBNL1 immunofluorescence showing nuclear CTG-CUG RNA foci and MBNL1 localization in DM1 cells cultured in differentiation medium for 48 hours and transfected with non-targeting siRNAs (si-NTC, A) or siRNAs targeting circARHGAP10 (si-cARHGAP10, B). CTG-CUG RNA foci are shown in red, MBNL1 in green, and nuclei in blue (Hoechst). Top panels show full optical fields; bottom panels display magnified views of the boxed regions highlighting individual nuclei (red-blue, green-blue, red-green-blue channels). Scale bars: 20 µm (overview), 5 µm (magnified views). (C–D) Quantification of MBNL1 sequestration into foci: (C) average number of MBNL1-positive foci per nucleus; (D) average percentage of nuclear area occupied by MBNL1-positive foci. Data are shown as mean ± SEM. Statistical significance was assessed using (C) generalized linear mixed-effects models (negative binomial) with Wald z-tests for foci per nucleus, and (D) with t-test with Satterthwaite's method for degrees of freedom estimation for area percentage ($*p < 0.05$, $**p < 0.01$).

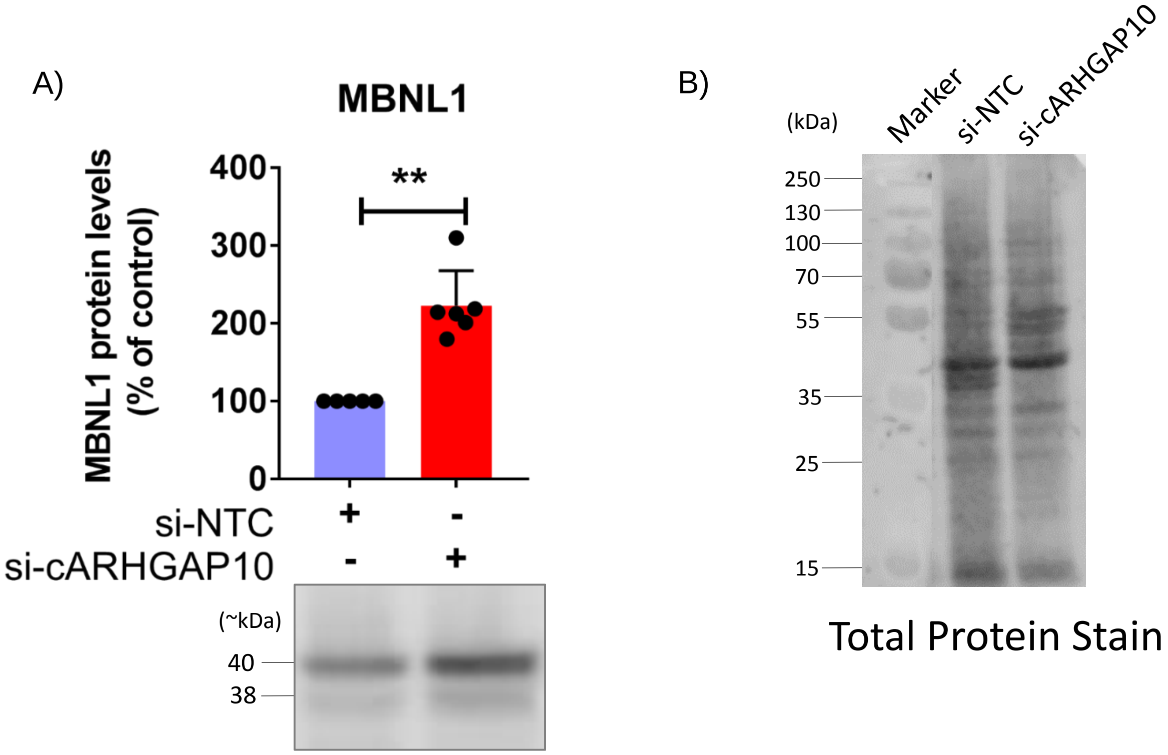

Figure S11. Increased MBNL1 protein levels in DM1 myogenic cells following circARHGAP10 silencing.

DM1 myogenic cells were transfected with siRNAs targeting circARHGAP10 (si-cARHGAP10) or non-targeting control siRNA (si-NTC) and cultured in differentiation medium (DM) for 48 hours. (A) Western blot analysis of MBNL1 protein expression. (B) Representative image of total protein staining used for normalization, performed using Revert™ Total Protein Stain (LI-COR) prior to immunodetection. Densitometric quantification of MBNL1 levels was normalized to total protein and expressed as a percentage relative to si-NTC-treated cells. Results are presented as mean ± SEM (n = 5). Statistical significance was determined using the Mann–Whitney U test (**$p < 0.01$)

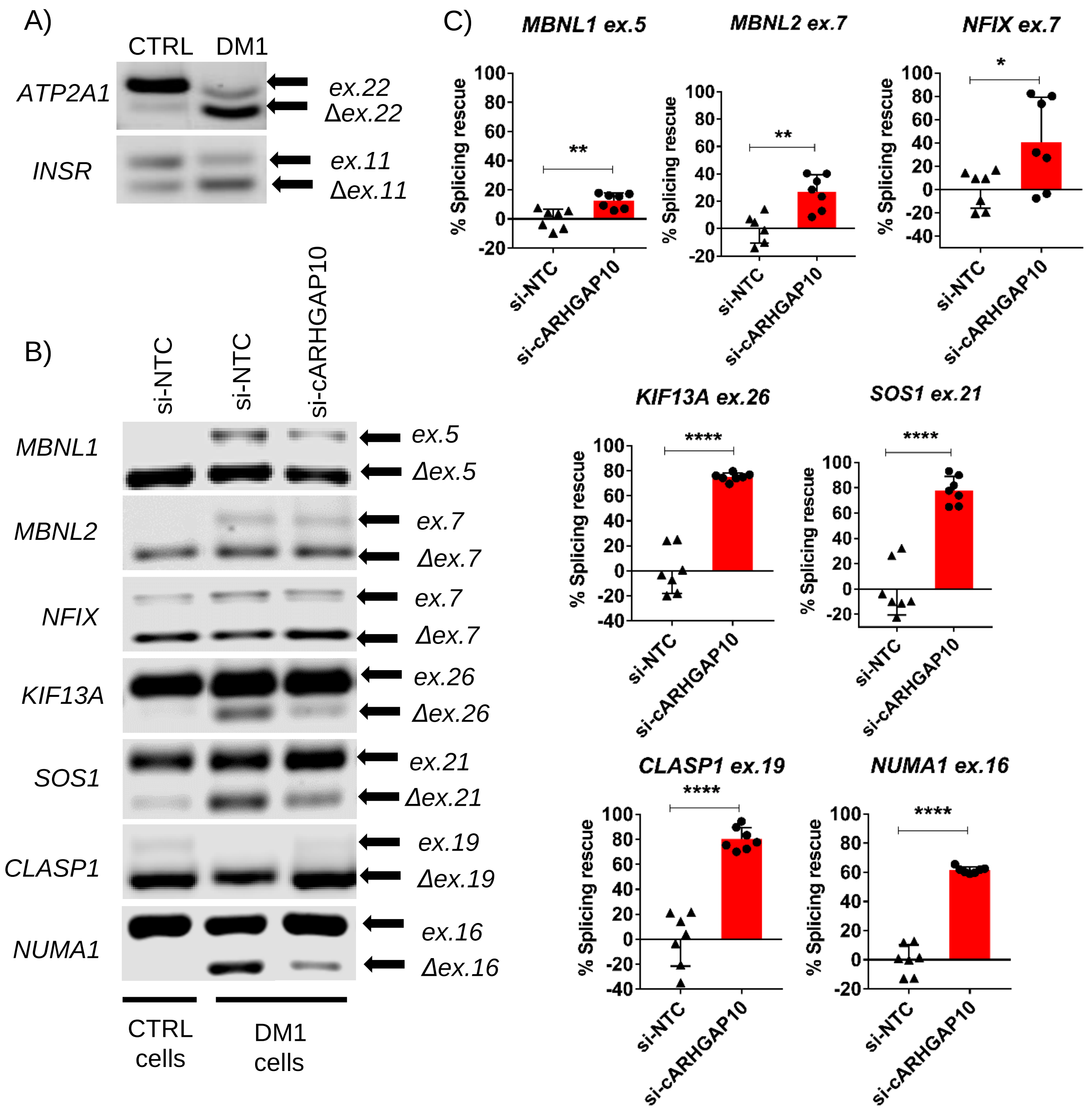

Figure S12. Partial rescue of MBNL1-regulated alternative splicing targets following circARHGAP10 silencing

(A) Representative agarose gel electrophoresis of RT-PCR products showing the alternative splicing of ATP2A1 exon 22 and INSR exon 11 in differentated control and DM1 myogenic cell cultures. (B) Agarose gel electrophoresis of RT-PCR products illustrating the splicing patterns of known MBNL1-regulated targets (MBNL1, MBNL2, NFIX, KIF13A, SOS1, CLASP1, and NUMA1) in differentiated control and DM1 myogenic cells transfected with siRNAs targeting circARHGAP10 (si-cARHGAP10) or non-targeting control siRNA (si-NTC). (C) Barplots showing quantification of splicing rescue (% Splicing Rescue) in DM1 cells treated with si-cARHGAP10 compared to si-NTC (n = 7). Percentage of splicing rescue was assessed after 36 hours of differentiation. Data are presented as mean ± SEM. Statistical significance was assessed using a two-tailed unpaired t-test (*$p < 0.05$, **$p < 0.01$, ****$p < 0.0001$).

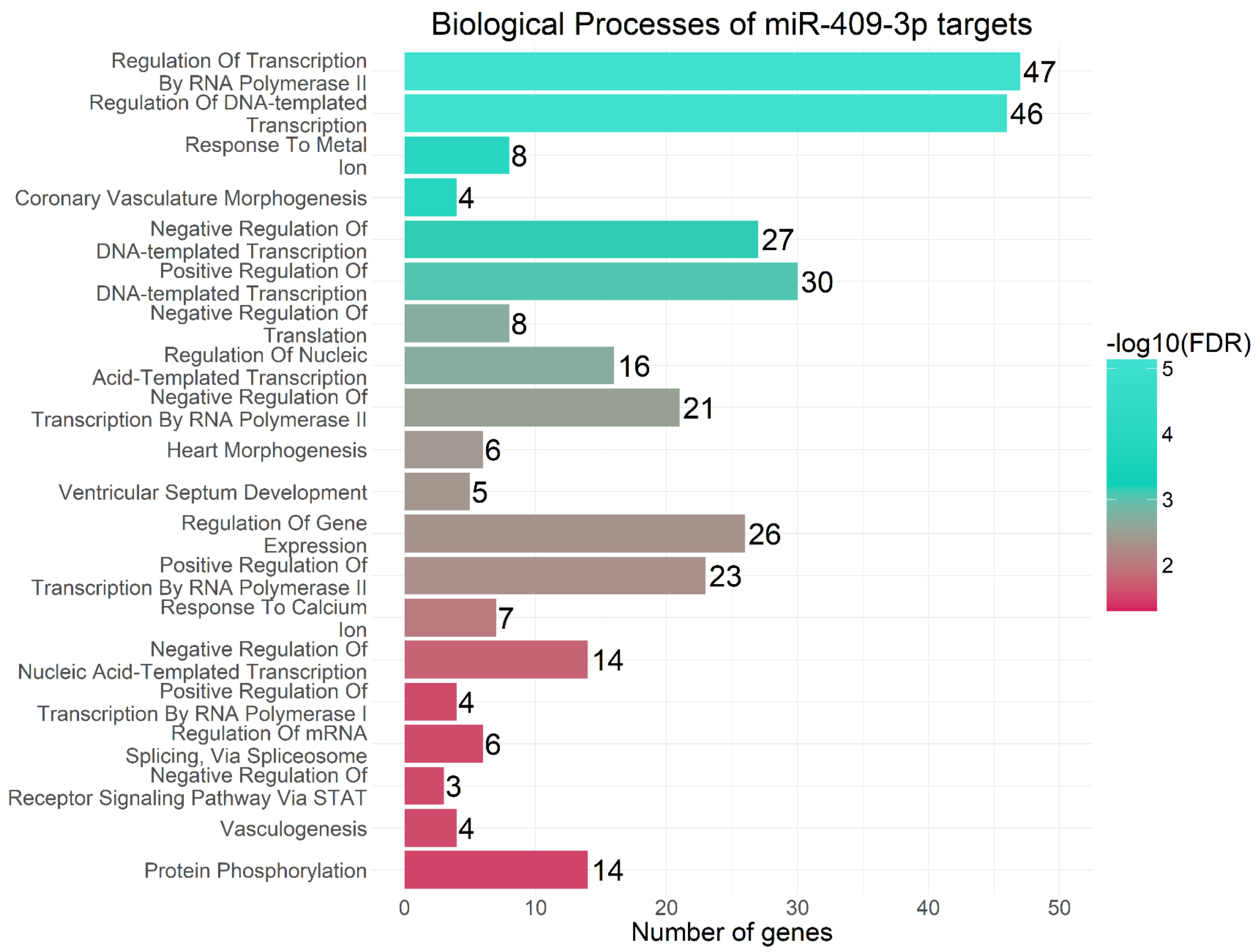

Figure S13. Analysis of Enriched Gene Ontology Biological Processes by miR-409-3p targets.

Results of enriched Gene Ontology Biological Processes analysis (top 20 significant terms) using EnrichR, based on miR-409-3p targets (TarBase v9.0, n=194). Color intensity indicates FDR values (range: [7.46×10^{-6}, 0.027]), with numbers on the bars representing the number of targets within each term.

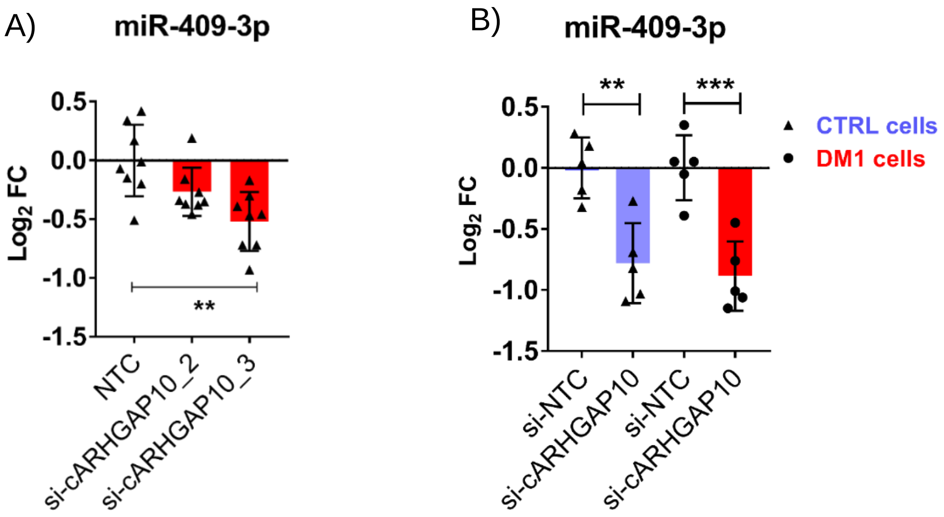

Figure S14. miR-409-3p is downregulated upon circARHGAP10 silencing.
(A) circARHGAP10 silencing downregulates miR-409-3p expression in DM1 myogenic cells. Barplots showing miR-409-3p expression levels (log_2 scale) measured by qPCR in DM1 myogenic cells transfected with two independent siRNAs targeting circARHGAP10 (si-cARHGAP10_2 and si-cARHGAP10_3). Data are presented as mean ± SEM (n = 8). (B) miR-409-3p expression in non-DM1 control (CTRL, blue) and DM1 (red) myogenic cells transfected with si-cARHGAP10 or non-targeting control (si-NTC). Data are presented in a log_2 scale as mean ± SEM (n = 4). Statistical analysis was performed using one-way ANOVA followed by Tukey's post hoc test, (**$p < 0.01$, ***$p < 0.001$).

Supplemental Tables:

Table S1. circRNA DM1 signature.

Table S2. Clinical characteristics of the patients recruited for the study.

Table S3. Sequences of qPCR primers and siRNAs.

Table S4. Annotation of TarBase targets of 409-3p regulating transcription.
